# Spatial lipidomic and neuron-specific transcriptomic signatures in the nucleus accumbens reveal phospholipid dyshomeostasis in depression-related maladaptations

**DOI:** 10.1038/s41398-026-04063-w

**Published:** 2026-05-14

**Authors:** Anderson Camargo, Ibrahim Kaya, Andrea Sturchio, Marcus Saarinen, Anna Kurgina, Ting Liang, Per E. Andrén, Per Svenningsson

**Affiliations:** 1 Department of Clinical Neuroscience, Translational Research on Neuropharmacology and Movement Disorders, Karolinska Institutet, Stockholm, Sweden; 2 Department of Pharmaceutical Biosciences, Spatial Mass Spectrometry, Science for Life Laboratory, Uppsala University, Uppsala, Sweden

**Keywords:** Molecular neuroscience, Depression

## Abstract

Lipid systems play a substantial role in the pathophysiological mechanisms underlying several brain disorders. However, the mechanisms underlying the lipid profile in stress-induced maladaptations and how these changes can impact distinct neuronal circuits and behavioral states remain to be fully elucidated. Here, we investigated the effects of p11 deficiency in combination with stress by using p11KO mice and chronic stress mouse models of depression to study spatial lipidomic and transcriptomic signatures in the mouse nucleus accumbens. Our results show that p11 deficiency and stress induce depression-related maladaptive phenotypes and provide novel evidence that these responses are associated with phospholipid dyshomeostasis in the nucleus accumbens. Phospholipid disturbances were predominantly related to phosphatidylethanolamine (PE) and ether PE metabolism, along with targets involved in the PE biosynthetic pathway. Moreover, chelerythrine administration, a compound reported to disrupt phospholipid balance, induces PE changes and depression-like behaviors. Altogether, the present study provides evidence that alterations in phospholipid-related pathways may alter reward/anti-reward circuits and how these changes might be implicated in stress-related disorders.

## Introduction

Stress-related psychiatric disorders, such as major depressive disorder and anxiety, are highly prevalent, relapsing, and among the leading contributors to the global burden of disease [1–3]. The treatment of these clinical manifestations remains a challenge, as the currently available gold-standard pharmacotherapy fails to adequately address all symptoms and carries the risk of severe side effects [3, 4]. Therefore, elucidating the underlying causal biological factors of stress-related conditions is crucial for identifying novel molecular therapy targets and, ultimately, developing more effective treatment strategies [5]. Lipid systems have been identified to play a substantial role in the pathophysiological mechanisms underlying brain disorders [6, 7]. They are a core component of brain tissue and, therefore essential to cell membrane structure, axonal myelination, neurotransmission, and signal transduction [8]. Homeostasis disturbances in the most abundant lipid class in the brain, phospholipids, have been described to alter brain function, potentially contributing to psychiatric symptoms [8–10]. Specifically, experimental studies have demonstrated alterations in phospholipid levels, including phosphatidylcholines (PC), phosphatidylserines (PS), and phosphatidylethanolamines (PE), in patients with depression and rodents with depression-like phenotype [11–14].

Although these findings have provided important insights into how lipid homeostasis is involved in the pathophysiology of depression, the mechanisms underlying the lipid profile in stress-induced maladaptations and how these changes can impact distinct neuronal circuits and behavioral states remain to be fully elucidated. Given its pivotal role in reward processing, stress regulation, and mood modulation, the nucleus accumbens emerges as a particularly compelling target for investigation [15–17]. For instance, studies have demonstrated that stress elicits a depression-like phenotype associated with synaptic changes in the nucleus accumbens of mice [18, 19]. In addition, nucleus accumbens deep-brain stimulation has been reported to induce the antidepressant effect in both mice and humans [20, 21]. In terms of functional and anatomic complexity, the nucleus accumbens is divided into core and shell segments, in which most of the neurons comprise GABAergic medium spiny neurons and a small percentage of cholinergic and GABAergic interneurons [22]. Of special interest, stress has been reported to disturb phosphatidylcholine turnover in the nucleus accumbens of mice with depression-like behavior, although the mechanisms and molecular targets associated with this response remain to be fully investigated [23].

The protein p11 (also named S100A10) has been shown to regulate depression-like states and mediate antidepressant responses in rodents [24–26]. p11 is enriched in the nucleus accumbens, particularly in cholinergic neurons [27], and genetic ablation of p11 in this brain region results in behavioral traits resembling depression in mice [28]. More importantly, patients diagnosed with depression present reduced p11 levels in the nucleus accumbens [28]. These findings suggest that p11 may serve as a key regulator of depression-like states [24], with its function in the nucleus accumbens potentially driving maladaptive behavioral outcomes. Given the established involvement of both p11 and stress in depression, we took advantage of global p11 knockout (p11KO) combined with a well-established restraint stress protocol [29, 30] to investigate the effects of these mouse models on lipid profile. Based on the hypothesis that p11 loss and stress could elicit maladaptations in the lipid system, which could underlie depression-related phenotypes, we evaluated global and targeted transcriptomic signatures and their association with lipidome alterations in the mouse nucleus accumbens tissue.

## Material and methods

Detailed information regarding the experimental design, including behavioral testing, matrix-assisted laser desorption/ionization-mass spectrometry imaging (MALDI-MSI), NanoString GeoMx® Digital Spatial Profiler (DSP), and fluorescent in situ hybridization (RNAscope), is included in the Supplementary Information.

### Animals

Wild-type (WT) and constitutive global p11KO mice were generated as previously described [31] on a C57BL/6J background. Genotypes were confirmed by PCR. The Karolinska Institutet Ethical Committee approved all experiments (3218–2022) according to Swedish guidelines in full compliance with European requirements.

### Repeated restraint stress protocol

To perform the restraint stress protocol, mice were subjected to immobilization (2 h/day, for 7 days) using a 50 ml Falcon tube [29].

### Drugs

Chelerythrine was purchased from Merk (Darmstadt, Germany), dissolved in sterile saline (0.9% NaCl with 2% DMSO), and administered via intraperitoneal (i.p.) route at a dose of 2 mg/kg [31, 32]. Chelerythrine was freshly prepared before administration and administered in a volume of 10 ml/kg body weight for 7 days.

### Behavioral tests

Animals were habituated to experimental conditions prior to behavioral tests and underwent behavioral testing as follows: social affective preference test (SAPT), sucrose preference test (SPT), open-field test (OFT), and tail suspension test (TST), 24 h apart [30, 33]. Mice were randomly assigned to the treatment groups, and observers were blinded to treatments and genotypes during the experiments and behavioral analysis. After the behavioral tests (24 h), mice were euthanized by decapitation, and the brains were collected and snap-frozen in isopentane, cooled in dry ice, and subsequently stored at −80 °C.

### Matrix-assisted laser desorption/ionization-mass spectrometry imaging (MALDI-MSI)

MALDI-MSI analysis of lipids in the nucleus accumbens was performed as previously reported [34]. Fresh-frozen sections were desiccated at room temperature for 15 min before spray coating of norharmane matrix solution. All MALDI-MSI experiments for lipid imaging were performed in both negative and positive ionization modes on the same tissue sections using a MALDI-FTICR (Solarix XR 7T-2ω, Bruker Daltonics) mass spectrometer equipped with a Smartbeam II 2 kHz laser. The average peak areas of the list of annotated-lipid species from each brain region in the mass range *m/z* 400–2000 were exported in both polarities from SCiLS for statistical analysis.

### NanoString GeoMx® Digital Spatial Profiler (DSP)

Consecutive fresh frozen sections were assessed using DSP, which was performed based on the barcoding technology (NanoString Technologies, Seattle, WA). This platform allows high-plex profiling at the RNA level, providing spatial assessment of tissue samples. Samples were assessed using the GeoMx mouse whole transcriptome atlas.

### Fluorescent in situ hybridization (RNA Scope)

Fluorescent in situ hybridization was performed using the RNAscope Multiplex Fluorescent Assay (Advanced Cell Diagnostics, Abingdon, Oxford) [33]. Consecutive fresh-frozen sections (12 μm thick) were hybridized with the probes: p11 (Mm-S100a10, #410901), NeuN (Ms-RBFOX3, #313311), Olig2 (Ms-Olig2, #447091), Iba1 (Ms-AIF1-C, #319141), SLC1A3 (Mm-SLC1A3, #430781), Gnpat (Mm-Gnpat, #1755391), and Phospho1 (Mm-Phospho1, #443631) for 2 h at 40 °C. Sections were imaged on a Carl Zeiss LSM 880 confocal microscope (Carl Zeiss AB, Stockholm, Sweden) using a 20× objective.

### Statistical analysis

The D’Agostino-Pearson test was used to assess data normality. The differences among experimental groups were determined by two-tailed unpaired Student’s t-test or two-way analysis of variance (ANOVA) followed by Tukey’s post hoc test, when appropriate. A value of *p* < 0.05 was considered significant. Differential abundance analysis of lipids and transcripts (shown in volcano plots) was performed using independent t-tests or linear mixed models, and the Benjamini-Hochberg procedure to control the false discovery rate (FDR < 0.05). Nonparametric data were analyzed using the Kruskal-Wallis test followed by Dunn’s multiple comparisons test. Data are presented as mean ± standard error of the mean (SEM). A value of *p* < 0.05 was considered significant. The details of statistical tests and their outcomes are presented in the Supplementary Information.

## Results

### p11 deficiency and stress induce depression-related maladaptive phenotypes

**Fig. 1:**
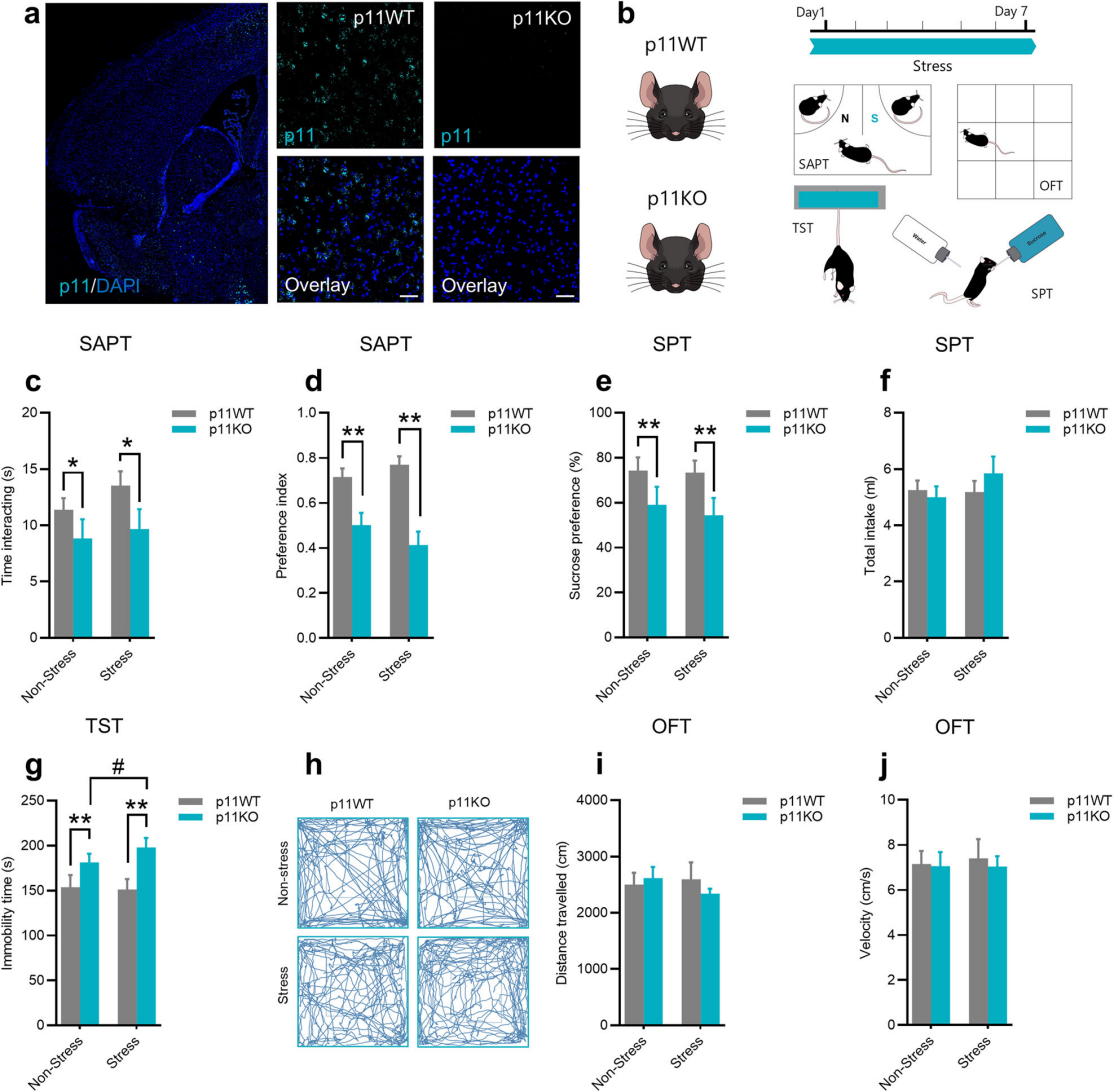
p11 deficiency and stress induce depression-related maladaptive phenotypes. **a** Representative fluorescent in situ hybridization image in the nucleus accumbens showing p11 (cyan) and DAPI (blue) staining in p11WT or p11KO mice. Scale bars: 100 µm. **b** Experimental time plan and behavioral tests in which p11WT or p11KO mice were subjected. **c** p11KO mice displayed reduced social interaction time in the social affective preference test (SAPT). **d** p11KO mice presented reduced social preference index in the SAPT. **e** p11KO mice exhibited an anhedonic-like phenotype in the sucrose preference test (SPT). **f** All experimental groups presented a similar total liquid (water + sucrose) intake. **g** p11KO mice presented a susceptible phenotype in the tail suspension test (TST) under stress conditions. **h** Representative track plots across all the experimental groups. **i** and **j** All experimental groups presented a similar distance travelled and velocity, respectively, in the open-field test (OFT). Values are expressed as means±S.E.M. (*n* = 8). *p < 0.05 and **p < 0.01 compared with the p11WT groups; #*p* < 0.05 compared with the p11KO group (two-way ANOVA followed by Tukey’s post hoc test).

Stress is the main environmental risk factor underlying the occurrence of depression [35, 36] and p11 deficiency has provided a valuable genetic model for investigating the molecular and physiological changes associated with depression [24]. For this reason, we took advantage of p11KO mice and the restraint stress protocol to explore the effects of p11 and stress combination on behavioral manifestations of depression-like phenotypes. To address this experimental approach, p11WT or p11KO mice (Fig. 1a) were exposed to the restraint stress procedure, which involved the placement in a 50 ml Falcon tube for 2 h per day over a 7-day period. This stress procedure is insufficient to induce a susceptible phenotype in naïve mice but effective in eliciting such a phenotype in mice with underlying genetically related susceptibility mechanisms [33].

Following this, mice were subjected to a behavioral battery (Fig. 1b), which included the SAPT (a marker of social affective state-related behavior) [37], SPT (an index of hedonic or reward-related responses) [38], TST (a measure of behavioral correlate of despair or hopelessness) [39], and OFT (a marker of ambulatory behavior) [40]. It is important to mention that nucleus accumbens modulation has been reported to regulate social preference, sucrose preference, and stress-coping phenotypes [41–43]. Our results showed that p11KO mice displayed a significant reduction in the time interacting (*p* < 0.05; Fig. 1c) and social preference index (*p* < 0.01; Fig. 1d) when compared to the p11WT in the SAPT, regardless of stress challenge. Additionally, p11KO mice displayed a marked decrease in sucrose preference in the SPT (*p* < 0.01, Fig. 1e) without changing the total intake (Fig. 1f). A significant increase in immobility in the TST (*p* < 0.01, Fig. 1g) was observed in p11KO compared to p11WT mice. However, no significant change was observed in the distance traveled (Fig. 1h, i), velocity (Fig. 1j), and time spent in the center (Suppl. Fig. 1) in the OFT. Notably, p11KO mice exhibited an exacerbated depression-like phenotype in response to stress exposure in the tail suspension test.

### Spatial lipidomics unveils that depression-like states are associated with phospholipid dyshomeostasis in the nucleus accumbens

**Fig. 2:**
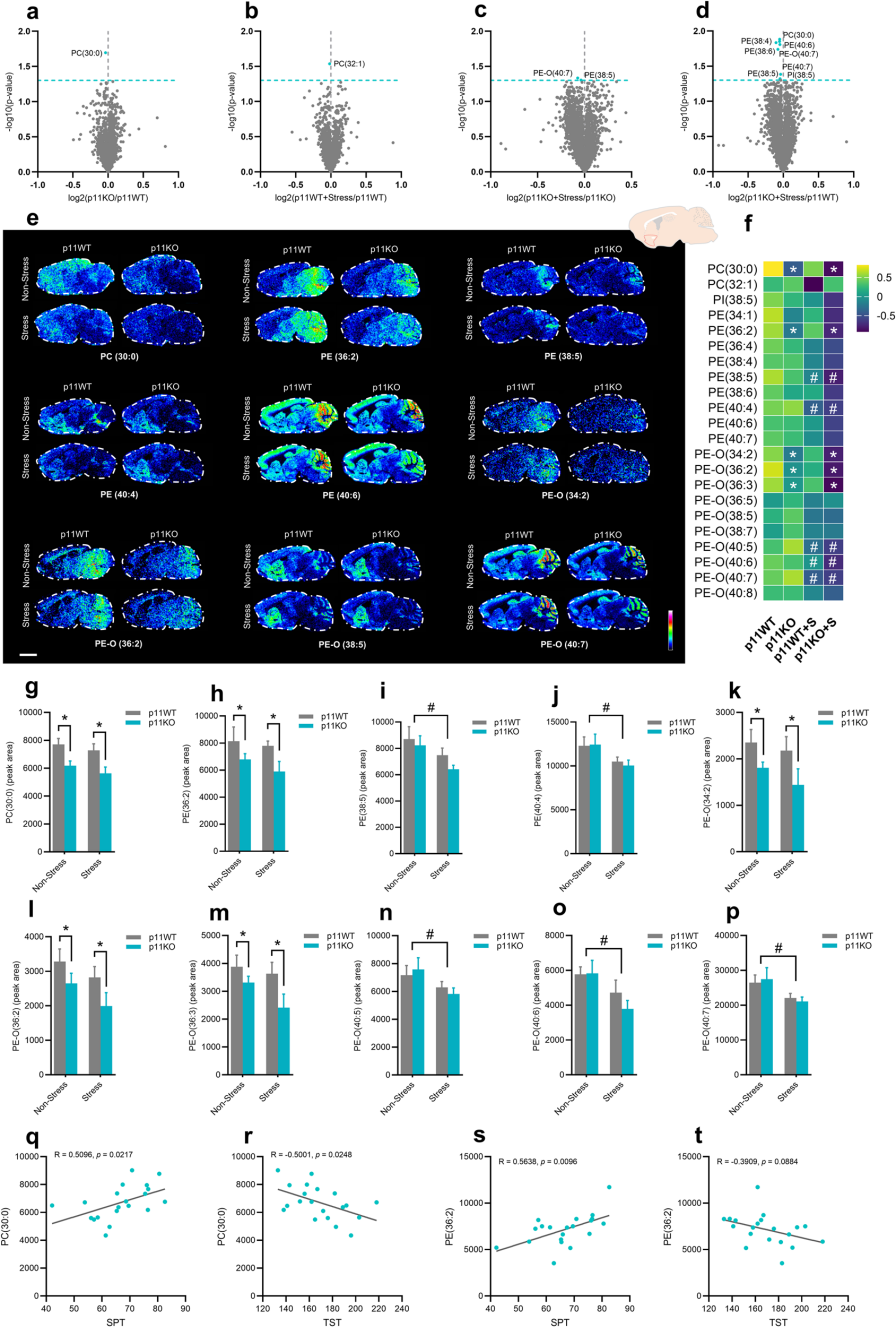
p11 and stress susceptibility are associated with phospholipid dyshomeostasis in the nucleus accumbens. Volcano plots showing the indicated fold differences and -log*10* (non-adjusted *p* value) of all detected lipids in the nucleus accumbens of **a** p11KO mice compared to p11WT mice, **b** p11WT+stress mice compared to p11WT mice, **c** p11KO+stress mice compared to p11KO mice, and **d** p11KO+stress mice compared to p11WT mice. **e** MALDI-MSI ion images of phospholipids, in particular phosphatidylcholine (PC), phosphatidylethanolamine (PE), and ether phosphatidylethanolamine (PE-O), sorted by number of double bonds or chain length. **f** Heat map of phospholipids and ether phospholipids in the nucleus accumbens of non-stressed or stressed p11WT or p11KO mice. Each square represents the z-score mean of the corresponding analyte with the value normalized across the groups. Bar graph showing the quantification of the peaks of PC(30:0) **g**, PE(36:2) **h**, PE(38:5) **i**, PE(40:4) **j**, PE-O(34:2) **k**, PE-O(36:2) **l**, PE-O(36:3) **m**, PE-O(40:5) **n**, PE-O(40:6) **o**, PE-O(40:7) **p**. MALDI-MSI ion images were presented as RMS normalized and acquired at a lateral resolution of 150 µm. Data are shown using a rainbow scale (representing ion intensity scale) for best visualization. Pearson´s correlation coefficient between PC(30:0) levels and **q** sucrose preference or **r** immobility time. Pearson´s correlation coefficient between PE(36:2) levels and **s** sucrose preference or **t** immobility time. Values are expressed as means±S.E.M. (*n* = 5). *p < 0.05 compared with the p11WT group (i.e., a significant main effect of genotype, two-way ANOVA); #*p* < 0.05 compared with non-stressed mice (i.e., a significant main effect of stress protocol, two-way ANOVA).

Given the role of lipid metabolism underlying the pathophysiology of depression [10, 44] and the involvement of the nucleus accumbens circuit in regulating affective states [16, 45], we sought to investigate how lipid metabolites were affected in the nucleus accumbens of p11WT and p11KO mice exposed or not to stress stimuli. Importantly, p11 is enriched in cholinergic neurons in the nucleus accumbens, where it regulates depression-like behaviors and responses to rewarding stimuli [28, 46]. We initially performed an untargeted spatial analysis of lipids in the nucleus accumbens using a high-throughput dual polarity MALDI-MSI method (Fig. 2a–d). In particular, we found significant alterations in phospholipid species with an evident depletion pattern, especially in p11KO mice under stress conditions (Fig. 2a–d). Given the unbiased and exploratory nature of this study, volcano plots display unadjusted p-values. We then conducted a targeted spatial lipidome analysis in p11WT and p11KO mouse brains. Differential abundance analysis across the measured groups revealed significant reductions of different phospholipid species, in particular phosphatidylcholine (PC)-, phosphatidylinositol (PI)-, phosphatidylethanolamine (PE)-, and ether phosphatidylethanolamine (PE-O)-related species (Fig. 2e; Suppl. Fig. 2).

Thus, we performed multiple *t*-tests to examine the phospholipid changes in the nucleus accumbens. Our results showed a significant reduction of PC(30:0) in p11KO compared to p11WT mice (*p* < 0.05), a significant decrease of PC(32:1) levels in p11WT+stress compared to p11WT group (*p* < 0.05), as well as a substantial reduction of PC(30:0) and PI(38:5) content in p11KO+stress compared to p11WT mice (*p* < 0.05; Suppl. Fig. 3; Suppl. Fig. 4). We also performed two-way ANOVA to examine those changes in the nucleus accumbens and found a significant main effect of p11 deficiency in reducing PC(30:0) levels (*p* < 0.05, Fig. 2f, g). Additionally, there was a trend of stress component in decreasing PI(38:5) levels (Fig. 2f; Suppl. Fig. 5). Furthermore, multiple *t*-tests analyses unveiled a significant reduction of PE(38:4), PE(38:5), PE(38:6), PE(40:6), and PE(40:7) levels in the nucleus accumbens of p11KO+stress compared to p11WT mice (*p* < 0.05, Suppl. Fig. 6; Suppl. Fig. 7). We also conducted two-way ANOVA analysis and found a significant main effect of genotype in decreasing PE(36:2) levels in the nucleus accumbens (*p* < 0.05, Fig. 2f, h; Suppl. Fig. 8), regardless of the stress stimulus. A significant main effect of stress on PE(38:5) and PE(40:4) content was observed (*p* < 0.05, Fig. 2f, i, and j; Suppl. Fig. 8), where stressed mice presented a significant reduction in these phospholipid levels in the nucleus accumbens. The results also demonstrated a significant reduction of PE-O species in the nucleus accumbens elicited by p11 deficiency or stress (Suppl. Fig. 9). In particular, we found reduced levels of PE-O(40:7) in p11KO+stress compared to p11KO group (*p* < 0.05) and PE-O(40:6) in p11KO+stress compared to p11WT group (*p* < 0.05, Suppl. Fig. 10). Two-way ANOVA revealed a significant main effect of p11 loss in depleting the levels of PE-O(34:2) (*p* < 0.05, Fig. 2f, k), PE-O(36:2) (*p* < 0.05, Fig. 2f, l), and PE-O(36:3) (*p* < 0.05, Fig. 2f, m) in the nucleus accumbens (Suppl. Fig. 11). A significant main effect of stress in reducing PE-O(40:5) (*p* < 0.05, Fig. 2f, n), PE-O(40:6) (*p* < 0.05, Fig. 2f, o), and PE-O(40:7) (*p* < 0.05, Fig. 2f, p) was also detected (Suppl. Fig. 11).

Interestingly, altered levels of phospholipid species in the nucleus accumbens are associated with the depression-like phenotype induced by p11 deficiency and stress. Of note, PC(30:0) and PE(36:2) levels positively correlate with the sucrose preference (*p* = 0.0217 and *p* = 0.0096, respectively; Fig. 2q, s), whereas PC(30:0) levels show a negative correlation with immobility time (*p* = 0.0248; Fig. 2r) (Suppl. Fig. 12). We also found that PE-O(34:2) positively correlates with social affective preference (*p* = 0.0461) and negatively with despair behavior (*p* = 0.0382). Similarly, PE-O(36:2) levels are negatively correlated with immobility time (*p* = 0.0329). In addition, PE-O(36:3) shows a positive correlation with sucrose preference (*p* = 0.0415) and a negative correlation with immobility time (*p* = 0.0437; Suppl. Fig. 13). These results suggest that phospholipid dyshomeostasis in the nucleus accumbens evoked by p11 loss and stress might contribute to depression-related maladaptive phenotypes.

### Neuron-specific transcriptome in the nucleus accumbens unveils alterations in targets related to phosphatidylethanolamine (PE) metabolism in p11 deficiency and stress susceptibility

**Fig. 3:**
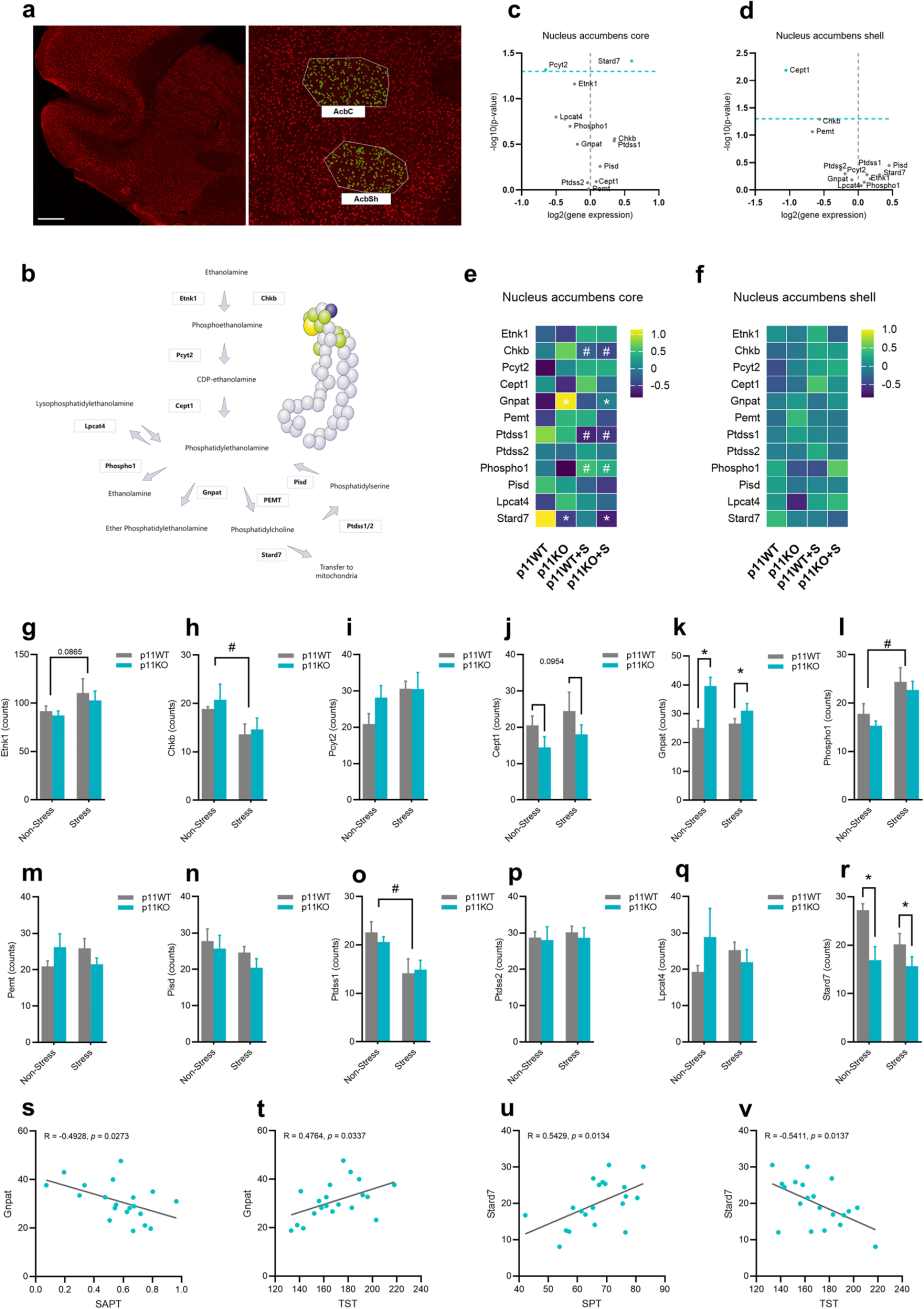
Neuron-specific transcriptome signature in the nucleus accumbens reveals alterations in molecular targets related to phosphatidylethanolamine (PE) metabolism in depression-like phenotypes. **a** Representative NeuN (red) staining image showing cell segmentation (green) in the nucleus accumbens core and shell. Scale bars: 500 µm. **b** Illustrative pathway related to phosphatidylethanolamine (PE) metabolism. The spheres represent different atoms present in the phosphatidylethanolamine structure, where *grey*: carbon, *green*: oxygen, *yellow*: phosphate, and *purple*: nitrogen (hydrogen atoms are not represented). Volcano plots showing the indicated fold differences and -log*10* (non-adjusted *p* value) of PE-related targets in the nucleus accumbens core **c** and shell **d** using linear mixed model. Heat map of targets involved in PE metabolism in the nucleus accumbens core **e** and shell **f** of non-stressed or stressed p11WT or p11KO mice. Each square represents the z-score mean of the corresponding protein with the value normalized across the groups. Bar graph showing the quantification of Etnk1 **g**, Chkb **h**, Pcyt2 **i**, Cept1 **j**, Gnpat **k**, Phospho1 **l**, Pemt **m**, Pisd **n**, Ptdss1 **o**, Ptdss2 **p**, Lpcat4 **q**, and Stard7 **r** in the nucleus accumbens core. Pearson´s correlation coefficient between Gnpat levels and social preference index **s** or immobility time **t**. Pearson´s correlation coefficient between stard7 levels and sucrose preference **u** or immobility time **v**. Values are expressed as means±S.E.M. (*n* = 5). *p < 0.05 compared with the p11WT group (i.e., a significant main effect of genotype, two-way ANOVA); #*p* < 0.05 compared with non-stressed mice (i.e., a significant main effect of stress protocol, two-way ANOVA).

To better understand the effects of phospholipid dyshomeostasis in the nucleus accumbens elicited by p11 loss and stress protocol, we next sought to spatially characterize the neuron-specific transcriptome using NanoString GeoMx DSP technology, which allows high-plex profiling of RNA abundance. We targeted the transcriptomic profile of neuronal cells since p11 is mostly enriched in this cell type (Suppl. Fig. 14), and neurons are central to responding and driving stress effects [47]. Thus, NeuN⁺ cells from the nucleus accumbens core and shell were segmented (Fig. 3a) and assessed using the mouse whole transcriptome atlas. The successful isolation of the neuronal cell population of nucleus accumbens was validated using selected transcript counts of genes enriched in neurons, oligodendrocytes, astrocytes, and microglia according to AGEA Fine Structure Search of the Allen brain atlas (Suppl. Fig. 15). Additionally, nucleus accumbens-enriched genes counts were also validated using AGEA (Suppl. Fig. 15). We initially performed an untargeted spatial analysis of transcripts in the nucleus accumbens core and shell of non-stressed and stressed p11WT and p11KO mice (Suppl. Fig. 16) and found significant alterations in several transcripts involved in the metabolism of lipids, including the PEs-related pathway (Suppl. Fig. 17).

Thereafter, we conducted a targeted spatial analysis in p11WT and p11KO mouse brains focusing on the PEs-linked metabolic pathway (Fig. 3b). The biosynthetic pathways related to PE are complex and dependent on different targets [48]. For instance, ethanolamine is phosphorylated to phosphoethanolamine by ethanolamine kinase (Etnk1) or choline/ethanolamine kinase (Chkb), which is subsequently converted to CDP-ethanolamine by phosphoethanolamine cytidylyltransferase 2 (Pcyt2). CDP-ethanolamine is then converted to phosphatidylethanolamine (PE) by choline/ethanolamine phosphotransferase (Cept1). It is important to mention that PE can enter remodeling pathways, such as: (i) conversion to/from lysophosphatidylethanolamine by action of lysophospholipid acyltransferase (LPCAT4), and (ii) breakdown of PE to ethanolamine and phosphate by phosphatase 1 (Phospho1). In addition, PE can also be targeted to ether-linked PE synthesis, involving the action of glyceronephosphate O-acyltransferase (Gnpat). PE can be further converted into phosphatidylcholine (PC) by phosphatidylethanolamine N-methyltransferase (PEMT) or into phosphatidylserine (PS) by phosphatidylserine synthase 1/2 (Ptdss1/2). Importantly, PS can also be converted back into PE by phosphatidylserine decarboxylase (Pisd), comprising a de novo pathway for PE synthesis. Finally, PEs can be transported to different cellular compartments, including mitochondria, with mitochondrial transport mediated by StAR-related lipid transfer protein 7 (Stard7).

Here, differential abundance analysis using a mixed linear model across the measured groups revealed significant alterations in targets involved in PE-related metabolism. Remarkably, a significant reduction in Pcyt2 levels (*p* < 0.05) and an increase in Stard7 content (*p* < 0.05) was observed in the nucleus accumbens core (Fig. 3c), whereas Cept1 levels (*p* < 0.05) were significantly decreased in the nucleus accumbens shell (Fig. 3d). Additionally, independent *t*-tests in the nucleus accumbens core revealed that Stard7 was significantly downregulated in p11KO, p11WT+stress, and p11KO+stress compared to p11WT (*p* < 0.05, Suppl. Fig. 18). We also found a significant increase in Gnpat (*p* < 0.05) levels in p11KO compared to p11WT, along with a significant elevation in Lpcat4/Pcyt2 (*p* < 0.05) and Phospho1 (*p* < 0.05) in p11WT+stress and p11KO+stress, respectively, compared to the p11WT group. A significant reduction in Ptdss1 (*p* < 0.05) levels was detected in p11WT+stress compared to p11KO and p11WT. Furthermore, no significant changes were found in the nucleus accumbens shell (Suppl. Fig. 18). We also performed two-way ANOVA to further assess those changes in the nucleus accumbens core (Fig. 3g–r) and found a significant main effect of p11 deficiency characterized by increased Gnpat (*p* < 0.05) and reduced Stard7 levels (*p* < 0.05). We also detected a significant main effect of stress in decreasing Chkb and Ptdss1 levels (*p* < 0.05), while increasing Phospho1 levels (*p* < 0.05). A marked trend, although not statistically significant, was observed for p11 deficiency in reducing Cept1 levels or stress in increasing Etnk1 content. No significant alterations were found in the nucleus accumbens shell (Suppl. Fig. 19).

Our results also demonstrated that PE biosynthetic pathway-related enzymes in the nucleus accumbens core correlate with the depression-like phenotype induced by p11 deficiency and stress (Suppl. Fig. 20). We found that Gnpat levels negatively correlate with the social affective preference (*p* = 0.0273, Fig. 3s) and positively with immobility time (*p* = 0.0337, Fig. 3t). In addition, Stard7 levels show a positive correlation with sucrose preference (*p* = 0.0134, Fig. 3u) and a negative correlation with immobility time (p = 0.0137, Fig. 3v). Taken together, these results suggest that targets involved in the PE synthesis pathway may contribute to PE lipid dyshomeostasis and play a role in stress-induced maladaptive responses associated with depression-like behaviors.

**Fig. 4:**
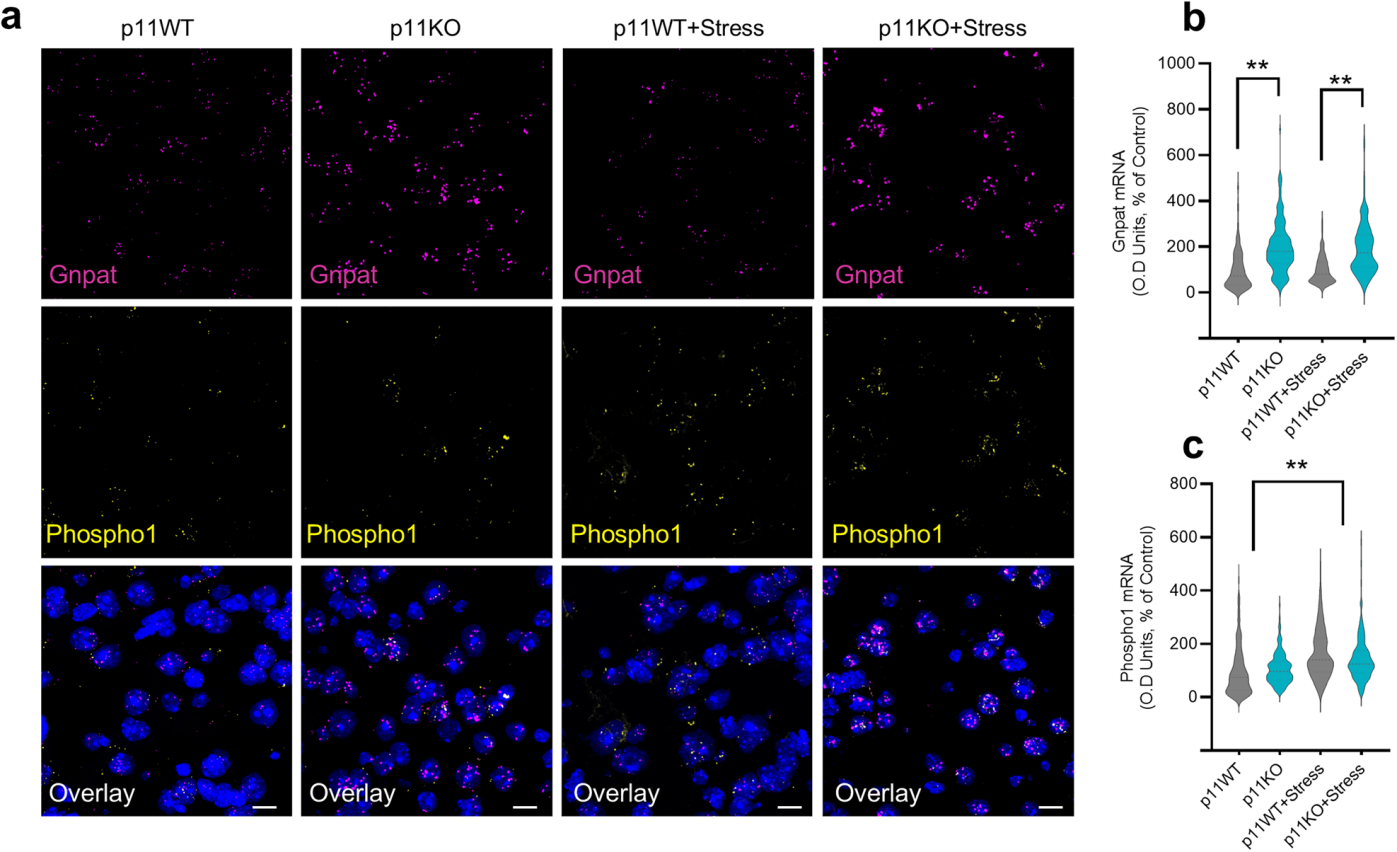
Altered levels of phosphatidylethanolamine (PE) pathway-related targets Gnpat and phospho-1 induced by p11 and stress. **a** Representative fluorescent in situ hybridization image in the nucleus accumbens core showing Gnpat (magenta), Phospho1 (yellow), and DAPI (blue) staining in p11WT or p11KO mice. Scale bars: 20 µm. **b** p11KO mice exhibited increased mRNA levels of Gnapt. **c** p11WT and p11KO mice exposed to stress presented increased mRNA levels of Phospho1. Quantification of fluorescence in individual Gnpat- or Phospho1-containing cells in the nucleus accumbens core (*n* = 150 positive cells from 5 mice). Values are expressed as means±S.E.M. (*n* = 5). **p < 0.01 compared with p11WT mice; ##*p* < 0.01 compared with non-stressed mice (Kruskal-Wallis test followed by Dunn’s multiple comparisons test).

We further measured the levels of these targets of the PE synthesis pathway in the nucleus accumbens core using fluorescent in situ hybridization (RNAscope). Kruskal-Wallis analysis confirmed the data from the transcriptomic analysis that p11KO mice displayed increased mRNA levels of Gnpat in the nucleus accumbens core (*p* < 0.01, Fig. 4a, b) compared to p11WT mice, regardless of stress protocol. Additionally, our results from the RNAscope experiments confirmed a significant increase in phospho1 mRNA levels in the nucleus accumbens core of stressed p11WT and p11KO mice (*p* < 0.01, Fig. 4a, c), compared to non-stressed counterparts.

### Behavioral effects of chelerythrine, an inhibitor of Cept1-dependent synthesis of choline- and ethanolamine-containing phospholipids

**Fig. 5:**
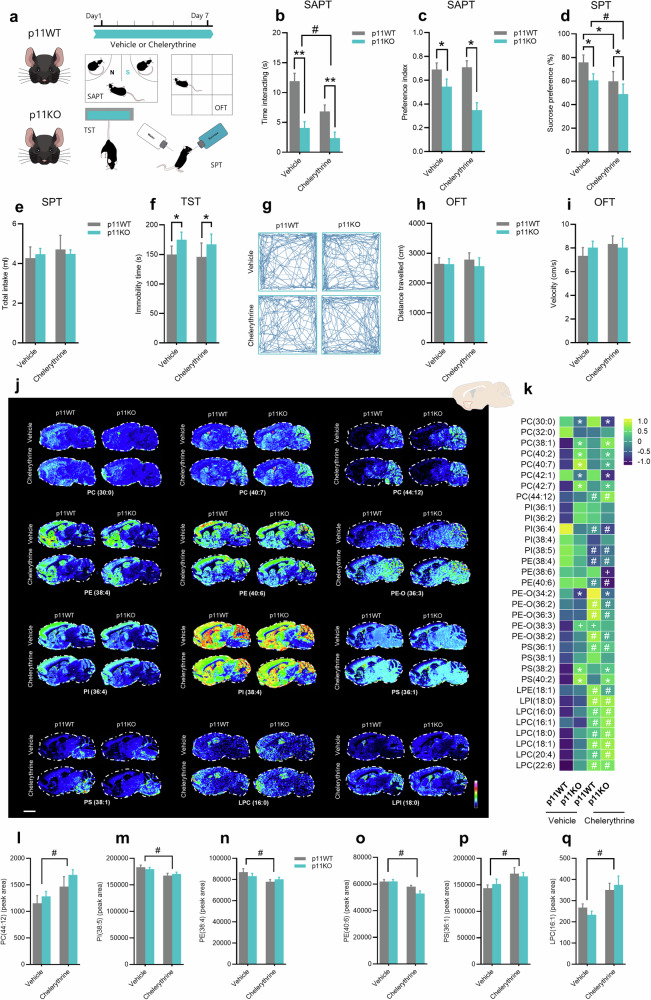
Behavioral effects of chelerythrine, an inhibitor of biosynthesis of choline-/ethanolamine-containing phospholipids. **a** Experimental time plan and behavioral tests in which p11WT or p11KO mice received vehicle or chelerythrine (2 mg/kg, i.p.) for 7 days. **b** p11WT and p11KO mice treated with chelerythrine displayed reduced social interaction time in the social affective preference test (SAPT). **c** p11KO mice presented reduced social preference index in the SAPT. **d** Chelerythrine-treated p11WT and p11KO mice exhibited an amplified anhedonic-like phenotype in the sucrose preference test (SPT). **e** All experimental groups presented a similar total liquid (water + sucrose) intake. **f** p11KO mice presented a passive stress-coping phenotype in the tail suspension test (TST). **g** Representative track plots across all the experimental groups. **h** and **i** All experimental groups presented a similar distance travelled and velocity, respectively, in the open-field test (OFT). Values are expressed as means±S.E.M. (*n* = 8). *p < 0.05 and **p < 0.01 compared with the p11WT groups; #*p* < 0.05 compared with the p11KO group (two-way ANOVA followed by Tukey’s post hoc test). **j** MALDI-MSI ion images of phospholipids related to phosphatidylcholine (PC), phosphatidylethanolamine (PE), ether phosphatidylethanolamine (PE-O), phosphatidylinositol (PI), and phosphatidylserine (PS) species, sorted by number of double bonds or chain length. **k** Heat map of phospholipids in the nucleus accumbens of chelerythrine-treated p11WT and p11KO. Each square represents the z-score mean of the corresponding analyte with the value normalized across the groups. Bar graph shows the quantification of the peaks of PC(44:12) **l**, PI(38:5) **m**, PE(38:4) **n**, PE(40:6) **o**, PS(36:1) **p**, and LPC(16:1) **q**. MALDI-MSI ion images were presented as RMS normalized and acquired at a lateral resolution of 150 µm. Data are shown using a rainbow scale (representing ion intensity scale) for best visualization. Values are expressed as means±S.E.M. (*n* = 8). *p < 0.05 compared with the p11WT group (i.e., a significant main effect of genotype, two-way ANOVA); #*p* < 0.05 compared with vehicle-treated mice (i.e., a significant main effect of chelerythrine, two-way ANOVA); +*p* < 0.05 compared with the p11WT group (two-way ANOVA followed by Tukey’s post hoc test).

To further assess the role of the phospholipid dyshomeostasis underlying depression-related maladaptive behaviors, a functional experiment was performed to manipulate the phospholipid-related biosynthetic pathway. In particular, we tested whether chelerythrine, a multitarget compound previously reported as an inhibitor of Cept1-dependent synthesis of choline-/ethanolamine-containing phospholipids [49, 50], could modulate depression-related stress-coping responses. To address this hypothesis, p11WT and p11KO received vehicle or chelerythrine (2 mg/kg, i.p.) for 7 days and were then tested in the depression-related behavioral paradigms (Fig. 5a). Our results showed that p11KO mice displayed a significant reduction in the time interacting (*p* < 0.01; Fig. 5b) and social preference index (*p* < 0.01; Fig. 5c) when compared to the p11WT in the SAPT, regardless of treatment. Moreover, chelerythrine administration caused a significant reduction in time interacting (*p* < 0.05) compared to the vehicle-treated groups (Fig. 5b, c). Additionally, p11KO mice showed a marked decrease in sucrose preference in the SPT (*p* < 0.05, Fig. 5d), regardless of the treatment, without changing the total intake (Fig. 5e). Chelerythrine administration also induced a significant reduction in sucrose preference (*p* < 0.05) compared to the vehicle-treated mice in the SPT (Fig. 5d). Notably, p11KO mice exhibited an exacerbated anhedonia-like phenotype in the SPT in response to chelerythrine administration (*p* < 0.05, Fig. 5d). There were no significant effects of chelerythrine administration on immobility time in the TST (Fig. 5f) and locomotion, velocity, and time spent in the center in the OFT (Fig. 5g–I, Suppl. Fig. 21).

Using MALDI-MSI method, we showed that chelerythrine-treated p11WT and p11KO mice exhibited phospholipid alterations in the nucleus accumbens (Suppl. Fig. 22), particularly species related to PC, PE, PS, and PI (Suppl. Figs. 23–28). Two-way ANOVA revealed a significant main effect of chelerythrine administration characterized by reduced levels of PI(36:4), PI(38:5), PE(38:4), and PE(40:6) in p11WT and p11KO mice (*p* < 0.05, Fig. 5j–q). Moreover, chelerythrine-treated p11WT and p11KO mice displayed increased levels of PC(44:12), PE-O(36:2), PE-O(36:3), and PE-O(38:2) (*p* < 0.05, Fig. 5j–q). Interestingly, we also found that chelerythrine administration elicited alterations in several different species of lysophospholipids, such as lysophosphatidylcholine (LPC), lysophosphatidylethanolamine (LPE), and lysophosphatidylinositol (LPI) in the nucleus accumbens (*p* < 0.05, Suppl. Figs. 29, 30). Our results also demonstrated that chelerythrine-induced alterations in phospholipids metabolism correlate with the depression-like phenotype (Suppl. Figs. 29, 30). We found that PE-O(40:6) and PI(36:4) levels positively correlate with the social affective preference (*p* = 0.0449 and *p* = 0.0371) and sucrose preference (*p* = 0.0011 and *p* = 0.0027). Also, PC(44:12) levels negatively correlate with the sucrose preference (*p* = 0.0257, Suppl. Fig. 31). In addition, we observed that the levels of LPI(18:0) (*p* = 0.0080), LPC(16:0) (*p* = 0.0210), LPC(16:1) (*p* = 0.0053), LPC(18:0) (*p* = 0.0312), LPC(18:1) (*p* = 0.0133), LPC(20:4) (*p* = 0.0032), and LPC(22:6) (*p* = 0.0024) negatively correlate with the sucrose preference, whereas LPC(20:4) levels positively correlate with social affective preference (*p* = 0.0366, Suppl. Fig. 32). These results further reinforce the notion that phospholipid dyshomeostasis in the nucleus accumbens might contribute to depression-related maladaptive phenotypes.

## Discussion

This study unveils that p11 deficiency and stress mouse models induce depression-related maladaptive phenotypes and provides novel evidence that these responses are associated with phospholipid dyshomeostasis in the nucleus accumbens, a central component of the limbic system involved in rewarding responses and stress-related disorders. In particular, our data showed that the disturbances in phospholipid levels are predominantly related to PE and its ether-linked counterpart PE-O species. Accordingly, our transcriptomic analysis also identifies alterations in molecular targets related to the PE biosynthetic pathway in the nucleus accumbens in p11 and stress susceptibility. Finally, our experiments also uncovered that chelerythrine, a multitarget and phospholipid-disrupting drug, elicits depression-like maladaptive behaviors.

The link between stressful life events and depressive episodes represents one of the most substantial findings in terms of the pathophysiology of this psychiatric disorder [3]. However, the mechanisms that mediate this association at the biological level have yet to be fully determined [51, 52]. Growing evidence underscores that p11 plays a significant role in stress-susceptibility to depression and mediates antidepressant responses [24–26]. Interestingly, patients diagnosed with depression present reduced p11 levels in the nucleus accumbens, whereas genetic ablation of p11 in the mouse nucleus accumbens results in behavioral traits resembling depression [28]. These findings suggest that p11 may serve as a key regulator of stress response, with its function in the nucleus accumbens potentially contributing to the development of stress-related disorders. Therefore, to better understand the effects of p11- and/or stress-driven mechanisms that contribute to behavioral correlates of depression-like phenotypes, we took advantage of p11KO mice and the restraint stress protocol. We employed a battery of behavioral paradigms relevant for nucleus accumbens function and stress/depression-like outcomes, since the modulation of this brain region has been implicated in the regulation of social preference, anhedonia-related behaviors such as sucrose preference, and stress-coping responses phenotypes [41–43]. Here, our experiments revealed that p11KO mice displayed a significant reduction in the social preference index in the SAPT and a marked decrease in sucrose preference in the SPT. Additionally, p11KO mice exhibited increased immobility in the TST without altering the locomotor activity and time in the center in the OFT. Notably, p11KO loss induced an exacerbated depression-like phenotype in response to stress exposure in the TST and a marked trend, though not statistically significant, in the SAPT and SPT. These results reinforce and expand previous findings from our group showing that p11 deficiency regulates stress-induced maladaptive behaviors [31, 39].

Emerging data have reported the role of lipid homeostasis underlying the pathophysiology of depression [10, 44]. To comprehensively shed light on the possible role of lipid systems underpinning depression-like phenotype induced by p11 loss and stress, we spatially characterized the lipidome profile in the nucleus accumbens. Using a high-throughput dual polarity MALDI-MSI method, we found significant alterations in phospholipid species with an evident depletion pattern, especially in p11KO mice under stress conditions. In particular, our results demonstrate that p11 loss induces a significant downregulation of PC(30:0) levels as well as PE and PE-O species, including PE(36:2), PE-O(34:2), PE-O(36:2), and PE-O(36:3) levels, in the nucleus accumbens. Moreover, we also observed that stress significantly reduced the levels of PE(38:5) and PE(40:4), and ether-linked species PE-O(40:5), PE-O(40:6), and PE-O(40:7). Our findings also suggest that p11 loss primarily reduced phospholipids with fewer double bonds (≤3 unsaturations), whereas the stress component led to a decrease in those with higher unsaturation levels (≥5 double bonds). Notably, those alterations in phospholipid levels in the nucleus accumbens correlate with the depression-like phenotype induced by p11 deficiency and stress. Accordingly, previous studies have revealed alterations in phospholipid levels in the plasma of patients with depression, particularly a significant reduction in total PE-O levels, which were negatively correlated with depression severity [13, 14]. In line, chronic unpredictable stress has also been reported to increase PI levels in the hippocampus and decrease PE levels in the prefrontal cortex of rodents, whereas no effects were observed in the amygdala or cerebellum [11]. Additionally, emerging evidence also indicates that the therapeutic effects of antidepressants extend beyond classical neurotransmitter targets and involve modulation of lipid-related pathways [53, 54]. Therefore, our results suggest that p11 deficiency and stress induce maladaptations to depression-like phenotypes associated with phospholipid alterations, predominantly PE metabolism, in the nucleus accumbens. Indeed, p11 is enriched in cholinergic interneurons, and while they comprise a small portion of neurons in the nucleus accumbens, compelling evidence has demonstrated that these neurons exert a disproportionately large influence on local circuits and neuromodulatory balance related to their widespread axonal arborizations [55]. Therefore, at the tissue level, one may suppose that p11 loss can elicit widespread changes in gene expression and lipid metabolism even though the primary genetic manipulation occurs in a small population.

To gain deeper insights into the effects of the p11 loss and stress mouse models on PE metabolism, we next sought to spatially characterize the neuron-specific transcriptome in the nucleus accumbens. Our untargeted spatial analysis in the nucleus accumbens core and shell uncovered significant alterations in several transcripts involved in the metabolism of lipids, including the PEs-related pathway. Indeed, PE species are the second most abundant phospholipid in the mammalian brain (~40% of total phospholipids), and they are required for a plethora of cellular processes [56]. In light of this, the biosynthetic pathways regulating PE levels are intricate and involve multiple targets [48] (see Fig. 3b). Our data show that p11 loss leads to a significant increase in Gnpat levels and a decrease in Stard7 levels in the nucleus accumbens core, but not in the shell. A decreasing trend in Cept1 levels associated with p11 deficiency was also observed, although not statistically significant. Additionally, stress was effective in reducing Chkb and Ptdss1 levels, while elevating Phospho1 levels specifically in the nucleus accumbens core. Based on these findings, reduced levels of PE/PE-O species caused by stress might be attributed to a downregulation of Chkb and upregulation of phospho1. Indeed, Chkb catalyzes the phosphorylation of ethanolamine, producing phosphoethanolamine, a precursor in the synthesis of CDP-ethanolamine and PE. Conversely, Phospho1 contributes to the degradation of PE, catalyzing its breakdown into ethanolamine and phosphate [48]. These findings suggest that stress may disrupt PE homeostasis by simultaneously limiting its synthesis precursors and promoting its degradation. In line with this assumption, the decreased levels of PE-related species elicited by p11 loss might also be linked to a potential reduction of Cept1 levels. Since Cept1 catalyzes the conversion of CDP-ethanolamine to PE, p11 deficiency might disrupt PE species by directly reducing their synthesis [48]. Furthermore, reduced levels of PE-O species in p11KO mice might be associated with an increase in Gnpat levels. PE can be directed toward the synthesis of PE-O, a Gnpat-dependent process that may reflect a compensatory mechanism [57]. More importantly, our results also demonstrated that Gnpat levels in the nucleus accumbens core correlate with the depression-like phenotype. A previous study has also reported that Gnpat-KO mice show deficient social interaction and passive stress-coping phenotypes [58, 59], further reinforcing the current results and the assumptions that phospholipid deficiency in mice induces behavioral alterations relevant to stress-related psychiatric disorders.

To further investigate how phospholipid dyshomeostasis contributes to depression-related maladaptive behaviors, we conducted a functional experiment using chelerythrine, to target the phospholipid biosynthetic pathway. While being a multitarget compound, previous reports identified that chelerythrine acts as an inhibitor of Cept1 at mid- to high micromolar concentrations [49, 50]. Remarkably, experimental animal models have disclosed that repeated administration of chelerythrine mimics depression symptoms like social avoidance, anhedonia, and hopelessness behaviors [56, 57]. Here, our results showed that chelerythrine administration caused a significant reduction in social interaction in both p11WT and p11KO mice. Interestingly, chelerythrine also induced a significant reduction in sucrose preference in p11WT mice, whereas p11KO mice exhibited an exacerbated anhedonia-like phenotype in the SPT in response to chelerythrine administration. These findings suggest that alterations in phospholipid-related pathways could be sufficient to make mice more prone to depression-related maladaptive behaviors. In line with our results, previous reports showed that repeated administration with chelerythrine effectively induced depression-like behavior in the forced swim test, social interaction test, and sucrose preference paradigm [56, 57]. Finally, using MALDI-MSI method, we confirmed that chelerythrine administration was effective in causing an imbalance in the phospholipid levels, predominantly PE- and PC-related species. Interestingly, we found that chelerythrine administration significantly led to a decrease in PE phospholipids with higher unsaturation levels, resembling those observed in chronically stressed mice. Our results also demonstrated that chelerythrine-induced alterations in phospholipid metabolism correlate with the depression-like phenotype. Thus, these findings further suggest that phospholipid dyshomeostasis might result in allostatic load and depression-related maladaptive phenotypes.

Altogether, our results suggest that the depression-like behavior induced by p11 deficiency and stress, in combination, is associated with phospholipid dyshomeostasis in the nucleus accumbens. Our data also unveils that phospholipid disturbances were predominantly related to PE and PE-O metabolism, as well as molecular targets involved in the PE biosynthetic pathway within the nucleus accumbens. Consistent with this, our findings demonstrate that chelerythrine, a compound known to disrupt phospholipid balance, induces depression-like behaviors. However, a limitation of our study is that chelerythrine is not a selective inhibitor of phospholipids, which may limit its specificity in targeting phospholipid-related pathways. Nevertheless, because phospholipid signaling pathways are complex, and in our study, both p11 deficiency and stress appeared to affect different targets within these pathways, we chose to use chelerythrine to comprehensively analyze the involvement of these pathways in our experimental model. Another caveat is that we did not directly assess whether chelerythrine crosses the blood–brain barrier at the dose used, nor did we specifically control for potential peripheral or nonspecific effects of chronic systemic administration. However, using a high throughput MALDI-MSI approach, we confirmed that chelerythrine administration was effective in causing an imbalance in the phospholipid levels in the nucleus accumbens of mice. Furthermore, future studies reintroducing p11 specifically into p11-expressing cells in the nucleus accumbens to assess the relationship between phospholipid alterations and p11-dependent behavioral outcomes would provide valuable mechanistic insights. Overall, the present provides novel insights into how phospholipid-related pathways and targets alter reward and anti-reward circuits and how these changes are implicated in stress-related disorders.

## Supplementary information

SUPPLEMENTAL MATERIAL

## Data Availability

The data that support the findings of this study are available from the corresponding author upon reasonable request.

## References

[1] James SL, Abate D, Abate KH, Abay SM, Abbafati C, Abbasi N, et al. Global, regional, and national incidence, prevalence, and years lived with disability for 354 diseases and injuries for 195 countries and territories, 1990–2017: a systematic analysis for the Global Burden of Disease Study 2017. The Lancet. 2018;392:1789–858. 10.1016/S0140-6736(18)32279-7 PMC6227754 30496104

[2] Radley J, Morilak D, Viau V, Campeau S. Chronic stress and brain plasticity: mechanisms underlying adaptive and maladaptive changes and implications for stress-related CNS disorders. Neurosci Biobehav Rev. 2015;58:79–91. 26116544 10.1016/j.neubiorev.2015.06.018 PMC4684432

[3] Cui L, Li S, Wang S, Wu X, Liu Y, Yu W, et al. Major depressive disorder: hypothesis, mechanism, prevention and treatment. Signal Transduct Target Ther. 2024;9:1–32. 38331979 10.1038/s41392-024-01738-y PMC10853571

[4] Otte C, Gold S, Penninx B, Pariante C, Etkin A, Fava M, et al. Major depressive disorder. Nat Rev Dis Primers. 2016;2:1–20. 10.1038/nrdp.2016.65 27629598

[5] Marwaha S, Palmer E, Suppes T, Cons E, Young AH, Upthegrove R. Novel and emerging treatments for major depression. The Lancet. 2023;401:141–53. 10.1016/S0140-6736(22)02080-3 36535295

[6] Di Donato I, Dotti MT, Federico A. Update on several/certain adult-onset genetic leukoencephalopathies: clinical signs and molecular confirmation. J Alzheimer’s Dis. 2014;42:S27–S35. 24958462 10.3233/JAD-141026

[7] Bremova-Ertl T, Schneider S. Current advancements in therapy for Niemann-Pick disease: progress and pitfalls. Expert Opin Pharmacother. 2023;24:1229–47. 37211769 10.1080/14656566.2023.2215386

[8] Osetrova M, Tkachev A, Mair W, Guijarro Larraz P, Efimova O, Kurochkin I, et al. Lipidome atlas of the adult human brain. Nat Commun. 2024;15:1–18. 38796479 10.1038/s41467-024-48734-y PMC11127996

[9] Yoon JH, Seo Y, Jo YS, Lee S, Cho E, Cazenave-Gassiot A, et al. Brain lipidomics: from functional landscape to clinical significance. Sci Adv. 2022;8:9317. 10.1126/sciadv.adc9317 PMC9481132 36112688

[10] Müller CP, Reichel M, Mühle C, Rhein C, Gulbins E, Kornhuber J. Brain membrane lipids in major depression and anxiety disorders. Biochim Biophys Acta. 2015;1851:1052–65. 25542508 10.1016/j.bbalip.2014.12.014

[11] Oliveira TG, Chan RB, Bravo FV, Miranda A, Silva RR, Zhou B, et al. The impact of chronic stress on the rat brain lipidome. Mol Psychiatry. 2016;21:80–8. 25754084 10.1038/mp.2015.14 PMC4565780

[12] Faria R, Santana MM, Aveleira CA, Simões C, Maciel E, Melo T, et al. Alterations in phospholipidomic profile in the brain of mouse model of depression induced by chronic unpredictable stress. Neuroscience. 2014;273:1–11. 24814727 10.1016/j.neuroscience.2014.04.042

[13] Liu X, Li J, Zheng P, Zhao X, Zhou C, Hu C, et al. Plasma lipidomics reveals potential lipid markers of major depressive disorder. Anal Bioanal Chem. 2016;408:6497–507. 27457104 10.1007/s00216-016-9768-5

[14] Liu X, Zheng P, Zhao X, Zhang Y, Hu C, Li J, et al. Discovery and validation of plasma biomarkers for major depressive disorder classification based on liquid chromatography-mass spectrometry. J Proteome Res. 2015;14:2322–30. 25784130 10.1021/acs.jproteome.5b00144

[15] Ding YD, Chen X, Chen ZB, Li L, Li XY, Castellanos FX, et al. Reduced nucleus accumbens functional connectivity in reward network and default mode network in patients with recurrent major depressive disorder. Transl Psychiatry. 2022;12:1–9. 35668086 10.1038/s41398-022-01995-x PMC9170720

[16] Jiang Y, Zou M, Wang Y, Wang Y. Nucleus accumbens in the pathogenesis of major depressive disorder: a brief review. Brain Res Bull. 2023;196:68–75. 36889362 10.1016/j.brainresbull.2023.03.004

[17] Francis TC, Lobo MK. Emerging role for nucleus accumbens medium spiny neuron subtypes in depression. Biol Psychiatry. 2017;81:645–53. 27871668 10.1016/j.biopsych.2016.09.007 PMC5352537

[18] Dudek KA, Paton SEJ, Binder LB, Collignon A, Dion-Albert L, Cadoret A, et al. Astrocytic cannabinoid receptor 1 promotes resilience by dampening stress-induced blood–brain barrier alterations. Nat Neurosci. 2025;28:766–82. 40016352 10.1038/s41593-025-01891-9 PMC11976283

[19] Heshmati M, Christoffel DJ, LeClair K, Cathomas F, Golden SA, Aleyasin H, et al. Depression and social defeat stress are associated with inhibitory synaptic changes in the nucleus accumbens. J Neurosci. 2020;40:6228–33. 32561672 10.1523/JNEUROSCI.2568-19.2020 PMC7406280

[20] Bewernick BH, Kayser S, Sturm V, Schlaepfer TE. Long-term effects of nucleus accumbens deep brain stimulation in treatment-resistant depression: evidence for sustained efficacy. Neuropsychopharmacology. 2012;37:1975–85. 22473055 10.1038/npp.2012.44 PMC3398749

[21] Zhou H, Zhu J, Jia J, Xiang W, Peng H, Zhang Y, et al. The antidepressant effect of nucleus accumbens deep brain stimulation is mediated by parvalbumin-positive interneurons in the dorsal dentate gyrus. Neurobiol Stress. 2022;21:100492. 36532368 10.1016/j.ynstr.2022.100492 PMC9755020

[22] Chen R, Blosser TR, Djekidel MN, Hao J, Bhattacherjee A, Chen W, et al. Decoding molecular and cellular heterogeneity of mouse nucleus accumbens. Nat Neurosci. 2021;24:1757–71. 34663959 10.1038/s41593-021-00938-x PMC8639815

[23] Cherix A, Larrieu T, Grosse J, Rodrigues J, McEwen B, Nasca C, et al. Metabolic signature in nucleus accumbens for anti-depressant-like effects of acetyl-l-carnitine. Elife. 2020;9:e50631. 31922486 10.7554/eLife.50631 PMC6970538

[24] Svenningsson P, Kim Y, Warner-Schmidt J, Oh YS, Greengard P. P11 and its role in depression and therapeutic responses to antidepressants. Nat Rev Neurosci. 2013;14:673–80. 24002251 10.1038/nrn3564 PMC3933996

[25] Seo JS, Svenningsson P. Modulation of Ion channels and receptors by p11 (S100A10). Trends Pharmacol Sci. 2020;41:487–97. 32418644 10.1016/j.tips.2020.04.004

[26] Svenningsson P, Chergui K, Rachleff I, Flajolet M, Zhang X, El Yacoubi M, et al. Alterations in 5-HT1B receptor function by p11 in depression-like states. Science. 2006;311:77–80. 16400147 10.1126/science.1117571

[27] Milosevic A, Liebmann T, Knudsen M, Schintu N, Svenningsson P, Greengard P. Cell- and region-specific expression of depression-related protein p11 (S100a10) in the brain. Journal of Comparative Neurology. 2017;525:955–75. 27616678 10.1002/cne.24113 PMC5222728

[28] Alexander B, Warner-Schmidt J, Eriksson TM, Tamminga C, Arango-Lievano M, Ghose S, et al. Reversal of depressed behaviors in mice by p11 gene therapy in the nucleus accumbens. Sci Transl Med. 2010;2:54–76. 10.1126/scitranslmed.3001079 PMC3026098 20962330

[29] Seo J, Wei J, Qin L, Kim Y, Yan Z, Greengard P. Cellular and molecular basis for stress-induced depression. Mol Psychiatry. 2017;22:1440–7. 27457815 10.1038/mp.2016.118 PMC5269558

[30] Dautan D, Camargo A, Branzell N, Brioschi VI, Doyon D, Covatta E, et al. Altered acetylcholine modulations and corticoaccumbal pathway in P11-linked social dysfunction. Mol Psychiatry. 2025;2025:1–14. 10.1038/s41380-025-03324-2 PMC12916318 41184570

[31] Ito T, Hiramatsu Y, Uchida M, Yoshimi A, Mamiya T, Mouri A, et al. Involvement of protein kinase C beta1-serotonin transporter system dysfunction in emotional behaviors in stressed mice. Neurochem Int. 2020;140:104826. 32818536 10.1016/j.neuint.2020.104826

[32] Einat H. Partial effects of the protein kinase C inhibitor chelerythrine in a battery of tests for manic-like behavior in black Swiss mice. Pharmacol Reports. 2014;66:722–5. 10.1016/j.pharep.2014.03.013 24948079

[33] Camargo A, Nilsson A, Shariatgorji R, Appleton E, Branzell N, Doyon D, et al. Enduring modulation of dorsal raphe nuclei regulates (R,S)-ketamine-mediated resilient stress-coping behavior. Mol Psychiatry. 2024;2024:1–13. 10.1038/s41380-024-02853-6 PMC12092261 39592824

[34] Kaya I, Nilsson A, Luptáková D, He Y, Vallianatou T, Bjärterot P, et al. Spatial lipidomics reveals brain region-specific changes of sulfatides in an experimental MPTP Parkinson’s disease primate model. NPJ Parkinsons Dis. 2023;9:1–10. 37495571 10.1038/s41531-023-00558-1 PMC10372136

[35] McEwen BS, Akil H. Revisiting the stress concept: implications for affective disorders. J Neurosci. 2020;40:12–21. 31896560 10.1523/JNEUROSCI.0733-19.2019 PMC6939488

[36] McEwen BS, Bowles NP, Gray JD, Hill MN, Hunter RG, Karatsoreos IN, et al. Mechanisms of stress in the brain. Nat Neurosci. 2015;18:1353–63. 26404710 10.1038/nn.4086 PMC4933289

[37] Rogers-Carter MM, Varela JA, Gribbons KB, Pierce AF, McGoey MT, Ritchey M, et al. Insular cortex mediates approach and avoidance responses to social affective stimuli. Nat Neurosci. 2018;21:404–14. 29379116 10.1038/s41593-018-0071-y PMC6051351

[38] Liu M-Y, Yin C-Y, Zhu L-J, Zhu X-H, Xu C, Luo C-X, et al. Sucrose preference test for measurement of stress-induced anhedonia in mice. Nat Protoc. 2018;13:1686–98. 29988104 10.1038/s41596-018-0011-z

[39] Steru L, Chermat R, Thierry B, Simon P. The tail suspension test: a new method for screening antidepressants in mice. Psychopharmacology. 1985;85:367–70. 3923523 10.1007/BF00428203

[40] Sousa VC, Mantas I, Stroth N, Hager T, Pereira M, Jiang H, et al. P11 deficiency increases stress reactivity along with HPA axis and autonomic hyperresponsiveness. Mol Psychiatry. 2020;26:3253–65. 33005029 10.1038/s41380-020-00887-0 PMC8505237

[41] Rogers-Carter MM, Djerdjaj A, Gribbons KB, Varela JA, Christianson JP. Insular cortex projections to nucleus accumbens core mediate social approach to stressed juvenile rats. J Neurosci. 2019;39:8717–29. 31591155 10.1523/JNEUROSCI.0316-19.2019 PMC6820210

[42] Martínez-Hernández J, Lanuza E, Martínez-García F. Lesions of the dopaminergic innervation of the nucleus accumbens medial shell delay the generation of preference for sucrose, but not of sexual pheromones. Behav Brain Res. 2012;226:538–47. 22019343 10.1016/j.bbr.2011.10.013

[43] Aizawa H, Cui W, Aida T, Ito H, Kobayashi K, Wada Y, et al. Dopaminergic signaling in the nucleus accumbens modulates stress-coping strategies during inescapable stress. Journal of Neuroscience. 2020;40:7241–54. 32847967 10.1523/JNEUROSCI.0444-20.2020 PMC7534921

[44] Schneider M, Levant B, Reichel M, Gulbins E, Kornhuber J, Müller CP. Lipids in psychiatric disorders and preventive medicine. Neurosci Biobehav Rev. 2017;76:336–62. 27317860 10.1016/j.neubiorev.2016.06.002

[45] Berridge KC, Kringelbach ML. Pleasure systems in the brain. Neuron. 2015;86:646–64. 25950633 10.1016/j.neuron.2015.02.018 PMC4425246

[46] Hanada Y, Kawahara Y, Ohnishi YN, Shuto T, Kuroiwa M, Sotogaku N, et al. P11 in cholinergic interneurons of the nucleus accumbens is essential for dopamine responses to rewarding stimuli. ENeuro. 2018;5:ENEURO.0332-18.2018. 30417079 10.1523/ENEURO.0332-18.2018 PMC6223111

[47] McEwen BS, Nasca C, Gray JD. Stress effects on neuronal structure: hippocampus, amygdala, and prefrontal cortex. Neuropsychopharmacology. 2015;41:3–23. 26076834 10.1038/npp.2015.171 PMC4677120

[48] Van der Veen JN, Kennelly JP, Wan S, Vance JE, Vance DE, Jacobs RL. The critical role of phosphatidylcholine and phosphatidylethanolamine metabolism in health and disease. Biochim Biophys Acta. 2017;1859:1558–72. 10.1016/j.bbamem.2017.04.006 28411170

[49] Wright MM, Henneberry AL, Lagace TA, Ridgway ND, McMaster CR. Uncoupling farnesol-induced apoptosis from its inhibition of phosphatidylcholine synthesis. J Biol Chem. 2001;276:25254–61. 11306571 10.1074/jbc.M011552200

[50] Wright MM, McMaster CR. PC and PE synthesis: mixed micellar analysis of the cholinephosphotransferase and ethanolaminephosphotransferase activities of human choline/ethanolamine phosphotransferase 1 (CEPT1). Lipids. 2002;37:663–72. 12216837 10.1007/s11745-002-0947-6

[51] Kalisch R, Russo SJ, Muller MB. Neurobiology and systems biology of stress resilience. Physiol Rev. 2024;104:1205–63. 38483288 10.1152/physrev.00042.2023 PMC11381009

[52] Nestler EJ, Russo SJ. Neurobiological basis of stress resilience. Neuron. 2024;112:1911–29. 38795707 10.1016/j.neuron.2024.05.001 PMC11189737

[53] Gulbins A, Schumacher F, Becker KA, Wilker B, Soddemann M, Boldrin F, et al. Antidepressants act by inducing autophagy controlled by sphingomyelin–ceramide. Mol Psychiatry. 2018;23:2324–46. 30038230 10.1038/s41380-018-0090-9 PMC6294742

[54] Gulbins E, Palmada M, Reichel M, Lüth A, Böhmer C, Amato D, et al. Acid sphingomyelinase–ceramide system mediates effects of antidepressant drugs. Nat Med. 2013;19:934–8. 23770692 10.1038/nm.3214

[55] Gonzales KK, Smith Y. Cholinergic interneurons in the dorsal and ventral striatum: Anatomical and functional considerations in normal and diseased conditions. Ann N Y Acad Sci. 2015;1349:1–45. 25876458 10.1111/nyas.12762 PMC4564338

[56] Wang B, Tontonoz P. Phospholipid remodeling in physiology and disease. Annu Rev Physiol. 2019;81:165–88. 30379616 10.1146/annurev-physiol-020518-114444 PMC7008953

[57] Da Silva TF, Sousa VF, Malheiro AR, Brites P. The importance of ether-phospholipids: a view from the perspective of mouse models. Biochim Biophys Acta. 2012;1822:1501–8. 22659211 10.1016/j.bbadis.2012.05.014

[58] Dorninger F, Gundacker A, Zeitler G, Pollak DD, Berger J. Ether lipid deficiency in mice produces a complex behavioral phenotype mimicking aspects of human psychiatric disorders. Int J Mol Sci. 2019;20:3929. 31412538 10.3390/ijms20163929 PMC6720005

[59] Dorninger F, König T, Scholze P, Berger ML, Zeitler G, Wiesinger C, et al. Disturbed neurotransmitter homeostasis in ether lipid deficiency. Hum Mol Genet. 2019;28:2046–61. 30759250 10.1093/hmg/ddz040 PMC6548223

