# Supplementary material for: Spatial lipidomic and neuron-specific transcriptomic signatures in the nucleus accumbens reveal phospholipid dyshomeostasis in depression-related maladaptations
Source: Transl Psychiatry. 2026 May 14;16:243. doi: 10.1038/s41398-026-04063-w (PMC13172476; doi:10.1038/s41398-026-04063-w)
Supplement: Supplementary file 1 — SUPPLEMENTAL MATERIAL [file 41398_2026_4063_MOESM1_ESM.docx]

Supplementary Information

**Spatial lipidomic and neuron-specific transcriptomic signatures in the nucleus accumbens reveal phospholipid dyshomeostasis in depression-related maladaptations**

Anderson Camargo^1^ *, Ibrahim Kaya^2^, Andrea Sturchio^1^, Marcus Saarinen^1^, Anna Kurgina^1^, Ting Liang^1^, Per E. Andrén^2^, Per Svenningsson^1^ *

1. Department of Clinical Neuroscience, Translational Research on Neuropharmacology and Movement Disorders, Karolinska Institutet, Stockholm, Sweden.

2. Department of Pharmaceutical Biosciences, Spatial Mass Spectrometry, Science for Life Laboratory, Uppsala University, Uppsala, Sweden.

* Corresponding authors

Dr. Anderson Camargo

Department of Clinical Neuroscience, Karolinska Institutet, Stockholm, Sweden.

E-mail address: anderson.camargo@ki.se

Dr. Per Svenningsson

Department of Clinical Neuroscience, Karolinska Institutet, Stockholm, Sweden.

E-mail address: per.svenningsson@ki.se

**Supplementary** **Material and Methods**

*Animals*

Rodents used in the current study included adult (8–12-week-old) female and male mice. Wild-type (WT) and constitutive global p11 knockout (p11KO) [1] on a C57BL/6J background. Mice were bred at the animal facility of Karolinska Institutet. Genotypes were confirmed by PCR. Mice were housed in groups of 4-5 in Type III Macrolon cages, under controlled temperature (21 ± 1 °C) and humidity (50 ± 20%) with a 12:12 h light/dark cycle (lights on at 7:00 a.m.), and with free access to food and water. All animals/samples were randomized. All experiments were approved by the Karolinska Institutet Ethical Committee (3218-2022) according to Swedish guidelines in full compliance with European requirements.

*Repeated restraint stress protocol*

To perform the chronic restraint stress protocol, mice were subjected to immobilization (2 h/day, for 7 days) using a 50 ml falcon tube. The stress protocol restrained all physical movement without submitting the animal to pain [2]. This procedure has been reported to be a submaximal restraint stress procedure insufficient for producing a susceptible phenotype in naïve mice [3]. On the testing day, 24 h after the last restraint stress episode, mice were subjected to behavioral tests.

*Drugs*

Chelerythrine was purchased from Merk (Darmstadt, Germany), dissolved in sterile saline (0.9% NaCl with 2% DMSO), and administered via intraperitoneal (i.p.) route at a dose of 2 mg/kg [4, 5]. Chelerythrine was freshly prepared before administration and administered in a volume of 10 ml/kg body weight for 7 days.

*Behavioral tests*

Animals were habituated to experimental conditions prior to behavioral tests and underwent behavioral testing as follows: social affective preference test, sucrose preference test, open-field test, and tail suspension test, 24 h apart. Mice were randomly assigned to the treatment groups and observers were blinded to treatments and genotypes during the experiments and behavioral analysis. After the behavioral tests (24 h), mice were euthanized by decapitation and the brains were collected and snap-frozen in isopentane, cooled in dry ice, and subsequently stored at -80°C.

*Social affective preference test (SAPT)*

The social affective preference test was performed as described previously [6]. Briefly, testing mice were habituated (6 min) inside a custom-made 3 chambers box equipped with a dark separator between two cylindrical cups that hosted the stimulus mice [7]. On the day of the experiments, the testing mouse was placed in the box for 6 minutes for acclimatization, then 2 stimulus mice were placed in a 5 cm radius plexiglass cup with holes drilled to allow contact and habituation. Next, both stimulus and cups were replaced with 2 fresh stimuli that either presented a neutral state or a stressed state (mice that underwent restraint stress using an individual rodent restraint device made of Plexiglas fenestrate for 15 min before the beginning of the trial), and the trial was recorded for 6 minutes [8]. The side for each stimulus was randomly assigned across experiments. The chambers were wiped with 75% ethanol and allowed to air dry between tests. Habituation and test videos were collected using a camera located above the apparatus, connected to Ethovision XT tracking software (Noldus). The videos were then scored offline by an experimenter blind using custom keyboard software. The time spent interacting was used to calculate the preference index, which was taken as a measure of social preference. The preference index was calculated as follows: index ([time spent sniffing the stressed state − time sniffing the neutral state] / [total time spent with both states]).

*Sucrose preference test (SPT)*

The sucrose preference index was measured as previously proposed [9]. Mice were individually housed and had ad libitum access to two bottles (one containing water and the other containing a 1,5 % sucrose solution) for a period of 24 h. The consumption of sucrose was calculated as a percentage of sucrose solution consumed relative to the total amount of liquid drunk. The sucrose preference was used as a measurement of stress-induced anhedonia-like behavior (hyposensitivity to pleasure marker) in mice.

*Open-field test (OFT)*

The locomotor activity was measured in the open field of a Plexiglas chamber (46 × 46 × 46 cm) with a grey floor and walls. The arena was illuminated by reflected light, providing an intensity of 30 Lux on the floor of the arena. Each mouse was placed in the corner of the open field, and locomotion was recorded for the indicated period of 6 min [1]. The arena was cleaned with 70% ethanol after each test session to eliminate olfactory cues. Video tracking was performed using a video camera mounted in the ceiling and analyzed by EthoVision XT11.5 (Noldus) software.

*Tail suspension test (TST)*

The total immobility time of mice suspended by the tail was measured as previously proposed [10]. Visually isolated mice were suspended 50 cm above the floor by adhesive tape placed approximately 1 cm from the tip of the tail. Immobility time was hand-scored for 6 min by an experienced observer blind to the experimental groups. Mice were considered immobile only when they hung passively and were completely motionless. The immobility time in the tail suspension test was taken as indicative of antidepressant-like or depressogenic-like effects.

*Matrix-assisted laser desorption/ionization-mass spectrometry imaging (MALDI-MSI)*

MALDI-MSI analysis of lipids in the nucleus accumbens was performed as previously reported [11]. Consecutive fresh frozen sections were desiccated at room temperature for 15 min before spray coating of norharmane matrix solution. Prior to matrix coating, the slide was scanned on a flatbed scanner (Epson perfection V500). The matrix solutions were prepared by dissolving the norharmane matrix powder in 80% MeOH (7.5 mg/ml) solution. An automated pneumatic sprayer (HTX-Technologies LLC, Chapel Hill, NC, USA) was used, which was combined with a pump (AKTA FPLC P-905 pump, Cytiva, Uppsala, Sweden) to spray heated matrix solution over the tissue sections. The pump was kept running at 100 μL/min using a 50% acetonitrile pushing solvent before the experiments to ensure a stable flow of the solvent with isocratic pressure. The matrix solution was sprayed using instrumental parameters of a solvent flow rate of 70 μL/min at isocratic pressure, a nitrogen flow of 6 psi, spray temperature of 60 °C, 15 passes with offsets and rotations, a nozzle head velocity of 1200 mm/min, and track spacing of 2.0 mm.

All MALDI-MSI experiments for lipid imaging were performed in both negative and positive ionization modes on the same tissue sections using a MALDI-FTICR (Solarix XR 7T-2ω, Bruker Daltonics) mass spectrometer equipped with a Smartbeam II 2 kHz laser. The size of the laser was chosen to give a lateral resolution of 150 μm in both polarities with an offset value of 75 μm to ensure no laser ablation overlaps between the polarity switch of the analysis. The instrument was tuned for optimal detection of lipid molecules (*m/z* 200–2000) in both polarities using the quadrature phase detection (QPD) (2ω) mode. The time-of-flight (TOF) values were set at 0.8 ms for positive and 1.0 ms for negative ion mode analysis and the transfer optics frequency was kept at 4 MHz for both polarity analyses. The quadrupole isolation *m/z* value (Q1 mass) was set at *m/z* 220.00 for both polarity analysis. Spectra were collected by summing 100 laser shots per pixel in both polarities. Both methods were calibrated externally with red phosphorus over an appropriate mass range. Ion signals of *m/z* 885.549853 (monoisotopic peak of [PI(38:4)-H]^-^) and *m/z* 798.540963 (monoisotopic peak of [PC(34:1) + K]^+^) were used for internal calibration for negative and positive polarity analysis, respectively. The laser power was optimized at the start of each analysis and then held constant during the MALDI-MSI experiment. Any possible bias due to factors such as matrix degradation or variation in mass spectrometer response was minimized by randomized analysis of the tissue sections.

The lipid ions were primarily identified by database searches (LIPID MAPS, Nature Lipidomics Gateway, www.lipidmaps.org) based on the high mass accuracy, MS/MS, and/or by comparing the observed distributions of the ion images of different adducts of the same molecule across the sagittal mouse brain tissue sections provided by the Fourier-transform ion cyclotron resonance (FTICR)-MS analysis (see Supplementary Table 1 for a detailed report of lipid annotations). MSI data were visualized in FlexImaging (v.5.0, Bruker Daltonics). For further analysis, data were imported to SCiLS Lab (v.2019a Pro, Bruker Daltonics), and brain regions were annotated according to Paxinos and Franklin’s stereotaxic atlas (see Supplementary Figure 31). All individual spectra were normalized to the root-mean-square (RMS) of all data points. The average peak areas of the list of annotated-lipid species from each brain region in the mass range *m/z* 400–2000 were exported in both polarities from SCiLS for statistical analysis.

*NanoString GeoMx® Digital Spatial Profiler (DSP)*

Fresh frozen mouse brains were cut at a thickness of 12 μm using a cryostat, thaw-mounted onto superfrost glass slides and stored at −80 °C. DSP was performed based on the barcoding technology (NanoString Technologies, Seattle, WA), which allows high-plex profiling at the RNA level, providing spatial assessment of tissue samples. Samples were assessed using GeoMx mouse whole transcriptome atlas and then immunostained with a NeuN primary antibody (1:500, #ABN91, Merck, Solna, Sweden). After 24 h incubation, sections were washed and incubated with Alexa Fluor 647-conjugated secondary antibodies (1:500, #A21449, Invitrogen, Stockholm, Sweden). After staining, the slides were scanned using the GeoMx DSP instrument (NanoString Technologies) and individual regions of interest (ROIs) covering the nucleus accumbens core and shell were generated and segmented using image analysis software embedded in the DSP device. Oligos from these compartments were released upon exposure to ultraviolet light sequentially, collected by microcapillary aspiration, dispensed into a 96-well plate, hybridized to fluorescent optical barcodes, and finally digitally counted using next-generation sequencing according to the manufacturer’s instructions (NanoString, Seattle, WA). Digital counts from barcodes corresponding to RNA probes underwent quality control (QC) analysis, where they were first normalized to internal spike-in controls and then to the area of their compartment. The third quartile (Q3) normalization was performed according to NanoString guidelines [12].

*Fluorescent in situ hybridization (RNA Scope)*

Fluorescent *in situ* hybridization was performed using the RNAscope Multiplex Fluorescent Assay (Advanced Cell Diagnostics, Abingdon, Oxford). Consecutive fresh-frozen sections (12 μm thick) were post-fixed in 4% paraformaldehyde (PFA) for 15 min at 4°C and dehydrated in graded alcohols. Next, Protease IV (Advanced Cell Diagnostics) was applied for 30 min at room temperature. Sections were then hybridized with the probes: p11 (Mm-S100a10, #410901), NeuN (Ms-RBFOX3, #313311), Olig2 (Ms-Olig2, #447091), Iba1 (Ms-AIF1-C, #319141), and SLC1A3 (Mm-SLC1A3, #430781) for 2 h at 40 °C [1]. In another batch of experiments, sections were then hybridized with the probes: Gnpat (Mm-Gnpat, #1755391) and Phospho1 (Mm-Phospho1, #443631) for 2 h at 40 °C. The hybridization step was followed by standardized steps of amplification (Amp 1-FL 30 min at 40 °C, Amp 2-FL 15 min at 40 °C, Amp 3-FL 30 min at 40 °C, Amp 4C-FL 15 min at 40 °C). After the last amplification step, sections were counterstained with DAPI (Advanced Cell Diagnostics), then mounted with Dako fluorescent mounting medium (Agilent Technologies, Kista, Sweden). Sections were imaged on a Carl Zeiss LSM 880 confocal microscope (Carl Zeiss AB, Stockholm, Sweden) using a 20x objective.

*Statistical analysis*

The statistical analyses were done using GraphPad Prism (GraphPad, San Diego, CA, USA). The D’Agostino-Pearson test was used to assess data normality. The differences among experimental groups were determined by Student's t-test or two-way analysis of variance (ANOVA) followed by Tukey’s post hoc test, when appropriate. Differential abundance analysis of lipids and transcripts (shown in volcano plots) was performed using independent t-tests or linear-mixed model, and the Benjamini-Hochberg multiple-testing correction to control the false discovery rate (FDR<0.05). Nonparametric data were analyzed using the Kruskal-Wallis test followed by Dunn's multiple comparisons test. All experiments were performed in a randomized manner, the experimenters were blinded, and the number of animals was chosen based on previous studies from our group. We did not assume sphericity and we used Geisser-Greenhouse correction, as recommended by Prism. Data are presented as mean ± standard error of the mean (SEM). A value of *p* < 0.05 was considered significant.

**Supplementary References**

1. Sousa VC, Mantas I, Stroth N, Hager T, Pereira M, Jiang H, et al. P11 deficiency increases stress reactivity along with HPA axis and autonomic hyperresponsiveness. Mol Psychiatry. 2020;26:3253–3265.

2. Seo JS, Wei J, Qin L, Kim Y, Yan Z, Greengard P. Cellular and molecular basis for stress-induced depression. Mol Psychiatry. 2016:1–8.

3. Shui Y, Wang L, Luo X, Uchiumi O, Yamamoto R, Sugai T, et al. Homer1a disruption increases vulnerability to predictable subtle stress normally sub-threshold for behavioral changes. Brain Res. 2015;1605:70–75.

4. Ito T, Hiramatsu Y, Uchida M, Yoshimi A, Mamiya T, Mouri A, et al. Involvement of protein kinase C beta1-serotonin transporter system dysfunction in emotional behaviors in stressed mice. Neurochem Int. 2020;140:104826.

5. Einat H. Partial effects of the protein kinase C inhibitor chelerythrine in a battery of tests for manic-like behavior in black Swiss mice. Pharmacol Rep. 2014;66:722–725.

6. Ng AJ, Vincelette LK, Li J, Brady BH, Christianson JP. Serotonin modulates social responses to stressed conspecifics via insular 5-HT2C receptors in rat. Neuropharmacology. 2023;236:109598.

7. Scheggia D, Managò F, Maltese F, Bruni S, Nigro M, Dautan D, et al. Somatostatin interneurons in the prefrontal cortex control affective state discrimination in mice. Nat Neurosci. 2020;23:47–60.

8. Ferretti V, Maltese F, Contarini G, Nigro M, Bonavia A, Huang H, et al. Oxytocin Signaling in the Central Amygdala Modulates Emotion Discrimination in Mice. Current Biology. 2019;29:1938-1953.e6.

9. Liu M-Y, Yin C-Y, Zhu L-J, Zhu X-H, Xu C, Luo C-X, et al. Sucrose preference test for measurement of stress-induced anhedonia in mice. Nat Protoc. 2018;13:1686–1698.

10. Steru L, Chermat R, Thierry B, Simon P. The tail suspension test: A new method for screening antidepressants in mice. Psychopharmacology. 1985;85:367–370.

11. He Y, Kaya I, Shariatgorji R, Lundkvist J, Wahlberg LU, Nilsson A, et al. Prosaposin maintains lipid homeostasis in dopamine neurons and counteracts experimental parkinsonism in rodents. Nat Comm. 2023;14:1–22.

12. Van Hijfte L, Geurts M, Vallentgoed WR, Eilers PHC, Sillevis Smitt PAE, Debets R, et al. Alternative normalization and analysis pipeline to address systematic bias in NanoString GeoMx Digital Spatial Profiling data. IScience. 2022;26:105760.

**Supplementary figures**


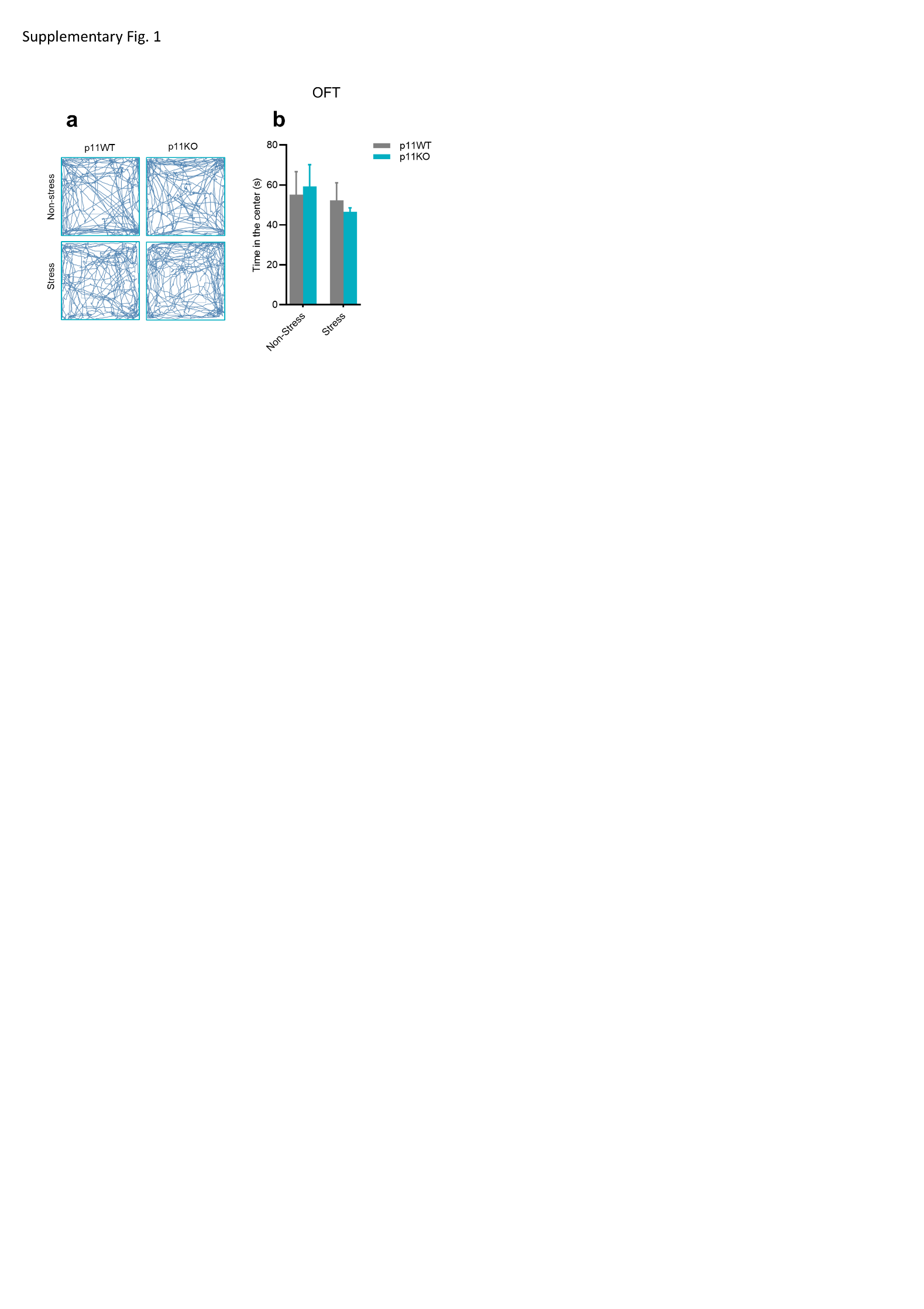


**Supplementary Fig. 1.** Behavioral effects of p11 deficiency and stress in the open-field test. **a** Representative track plots across all the experimental groups. **b** All experimental groups presented a similar distance time spent in the center in the open-field test (OFT). Values are expressed as means ± S.E.M. (*n* = 8). Two-way ANOVA analysis.


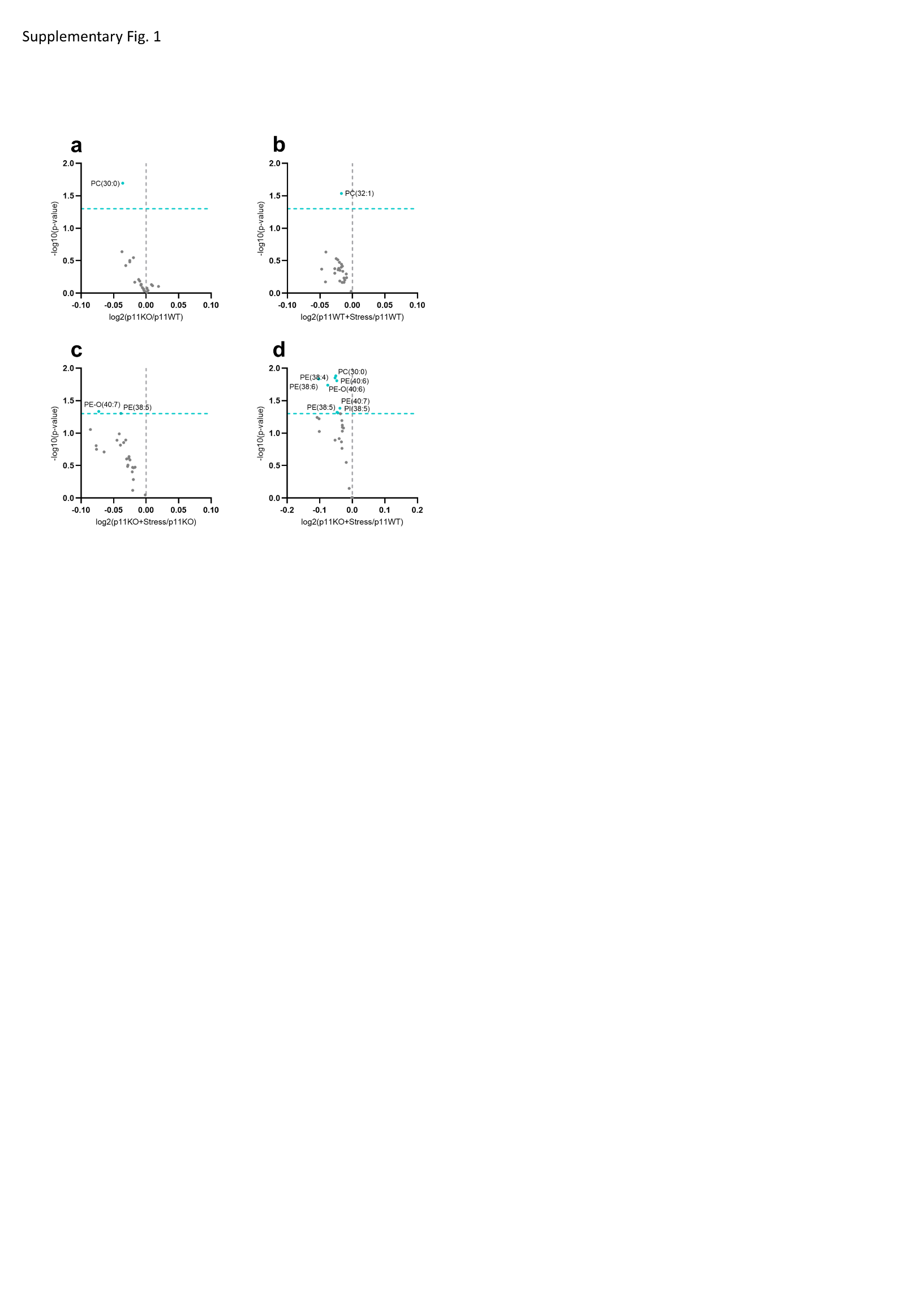


**Supplementary Fig. 2.** p11 deficiency and stress induce phospholipid dyshomeostasis in the nucleus accumbens. Volcano plots showing the indicated fold differences and -log*10* (non-adjusted *p* value) of phospholipids, in particular phosphatidylinositol (PC), phosphatidylethanolamine (PE), and ether phosphatidylethanolamine (PE-O) in the nucleus accumbens of (**a**) p11KO mice compared to p11WT mice, (**b**) p11WT+stress mice compared to p11WT mice, (**c**) p11KO+stress mice compared to p11KO mice, and (**d**) p11KO+stress mice compared to p11WT mice. The green dashed line represents FDR (*p*) = 5%. Each dot depicts one lipid.


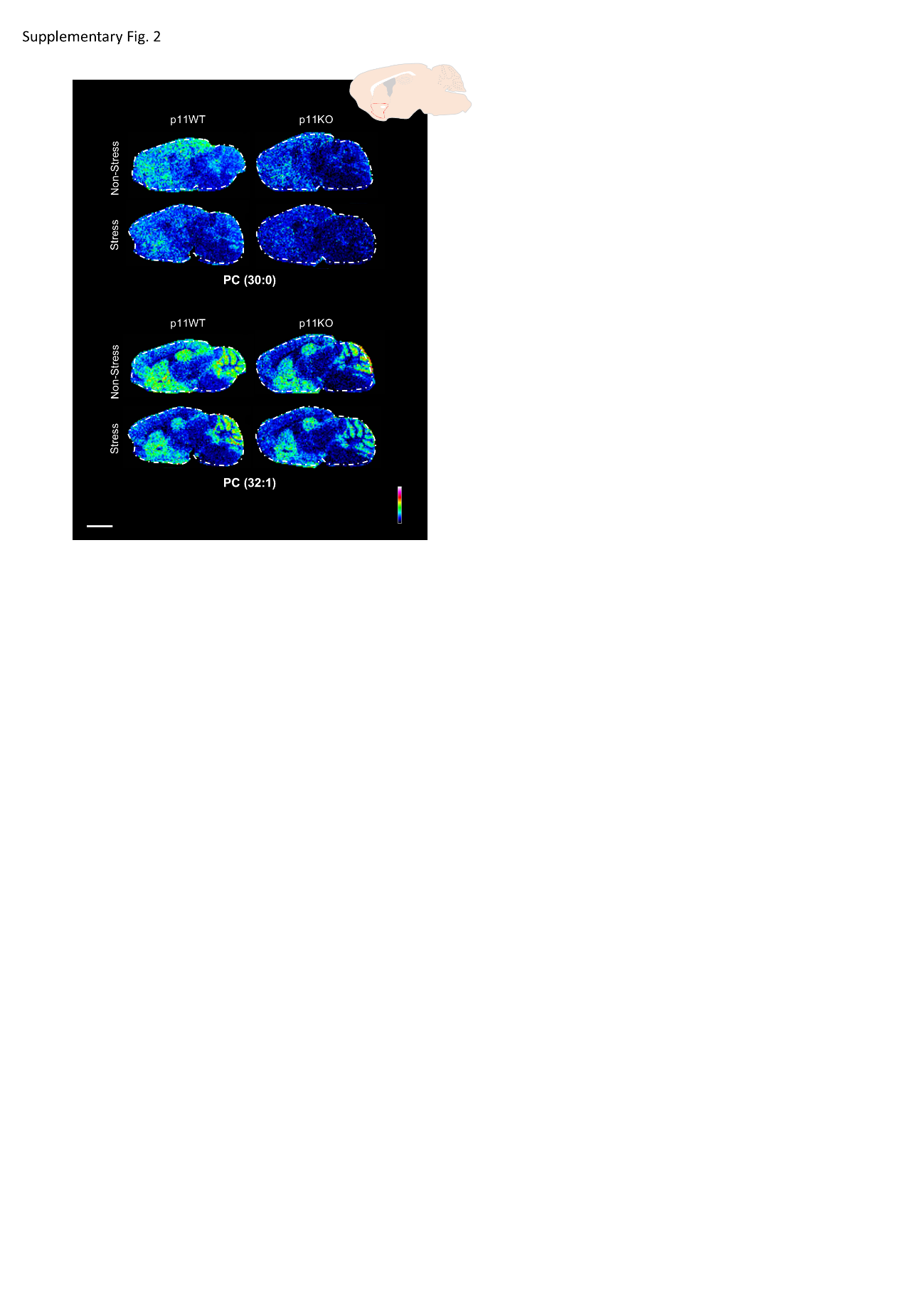


**Supplementary Fig. 3.** Effects of p11 deficiency and stress on phosphatidylcholine (PC) levels in the nucleus accumbens. Representative images of PC(30:0) and PC(32:1) in the nucleus accumbens of stressed or non-stressed p11WT or p11KO mice. MALDI-MSI ion images were presented as RMS normalized and acquired at a lateral resolution of 150 µm. Data are shown using a rainbow scale (representing ion intensity scale) for best visualization.


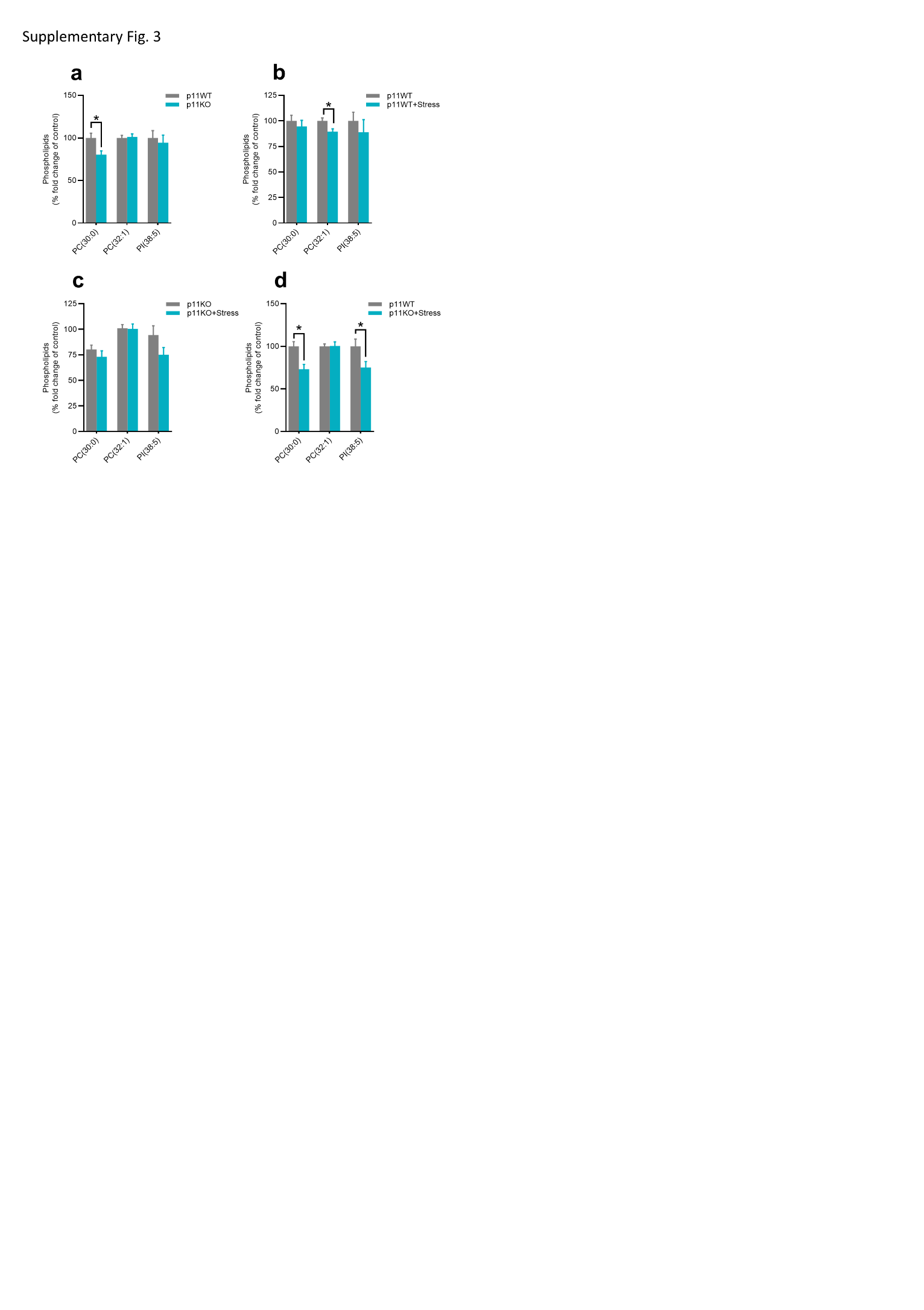


**Supplementary Fig. 4.** Effects of p11 deficiency and stress on phosphatidylcholine (PC) and phosphatidylinositol (PI) levels in the nucleus accumbens. Bar graph showing the quantification of the peaks of PC(30:0), PC(32:1), and PI(38:5) in the nucleus accumbens of (**a**) p11KO mice compared to p11WT mice, (**b**) p11WT+stress mice compared to p11WT mice, (**c**) p11KO+stress mice compared to p11KO mice, and (**d**) p11KO+stress mice compared to p11WT mice. Values are expressed as means ± S.E.M. (*n* = 5). **p* < 0.05 (Student’s *t* test).


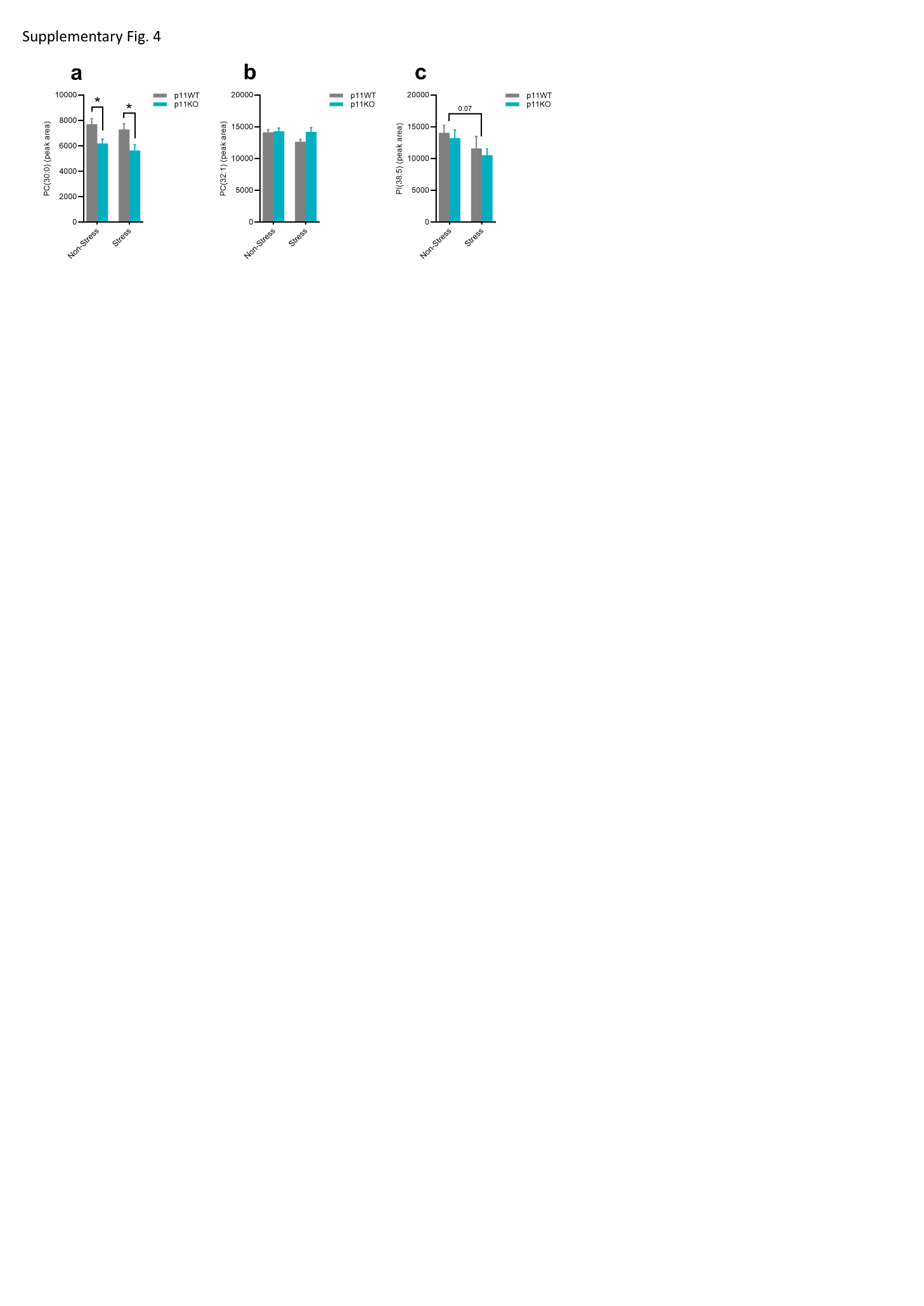


**Supplementary Fig. 5.** Altered levels of phosphatidylcholine (PC) and phosphatidylinositol (PI) in the nucleus accumbens induced by p11 deficiency and stress. Bar graph showing the quantification of the peaks of PC(30:0), PC(32:1), and PI(38:5) in the nucleus accumbens. Values are expressed as means ± S.E.M. (*n* = 5). **p* < 0.05 compared with the p11WT group (i.e., a significant main effect of genotype, two-way ANOVA).


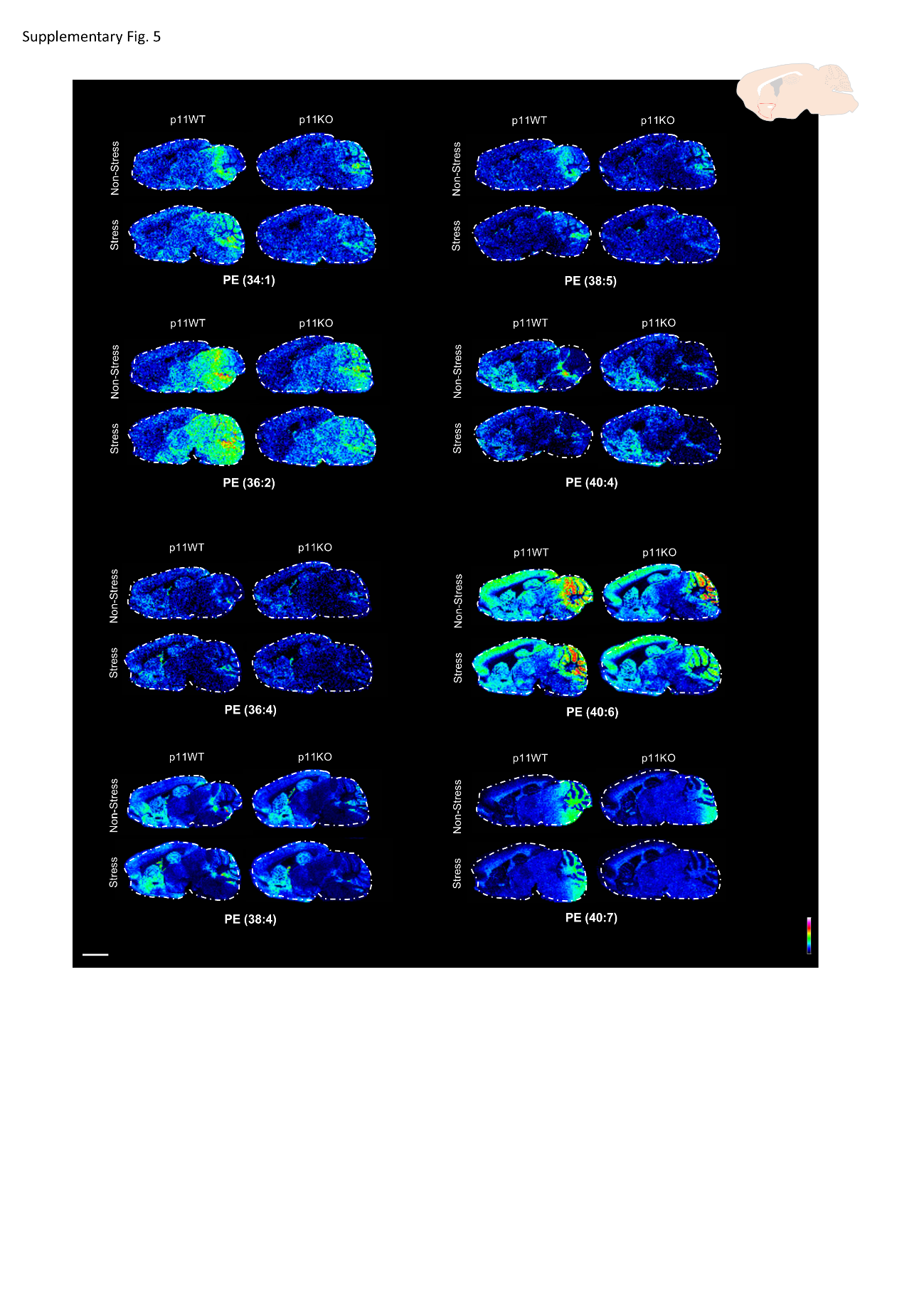


**Supplementary Fig. 6.** Effects of p11 deficiency and stress on phosphatidylethanolamine (PE) species levels in the nucleus accumbens. Representative ion images of PE(34:1), PE(36:2), PE(36:4), PE(38:4), PE(38:5), PE(40:4), PE(40:6), and PE(40:7) in the nucleus accumbens of stressed or non-stressed p11WT or p11KO mice. MALDI-MSI ion images were presented as RMS normalized and acquired at a lateral resolution of 150 µm. Data are shown using a rainbow scale (representing ion intensity scale) for best visualization.


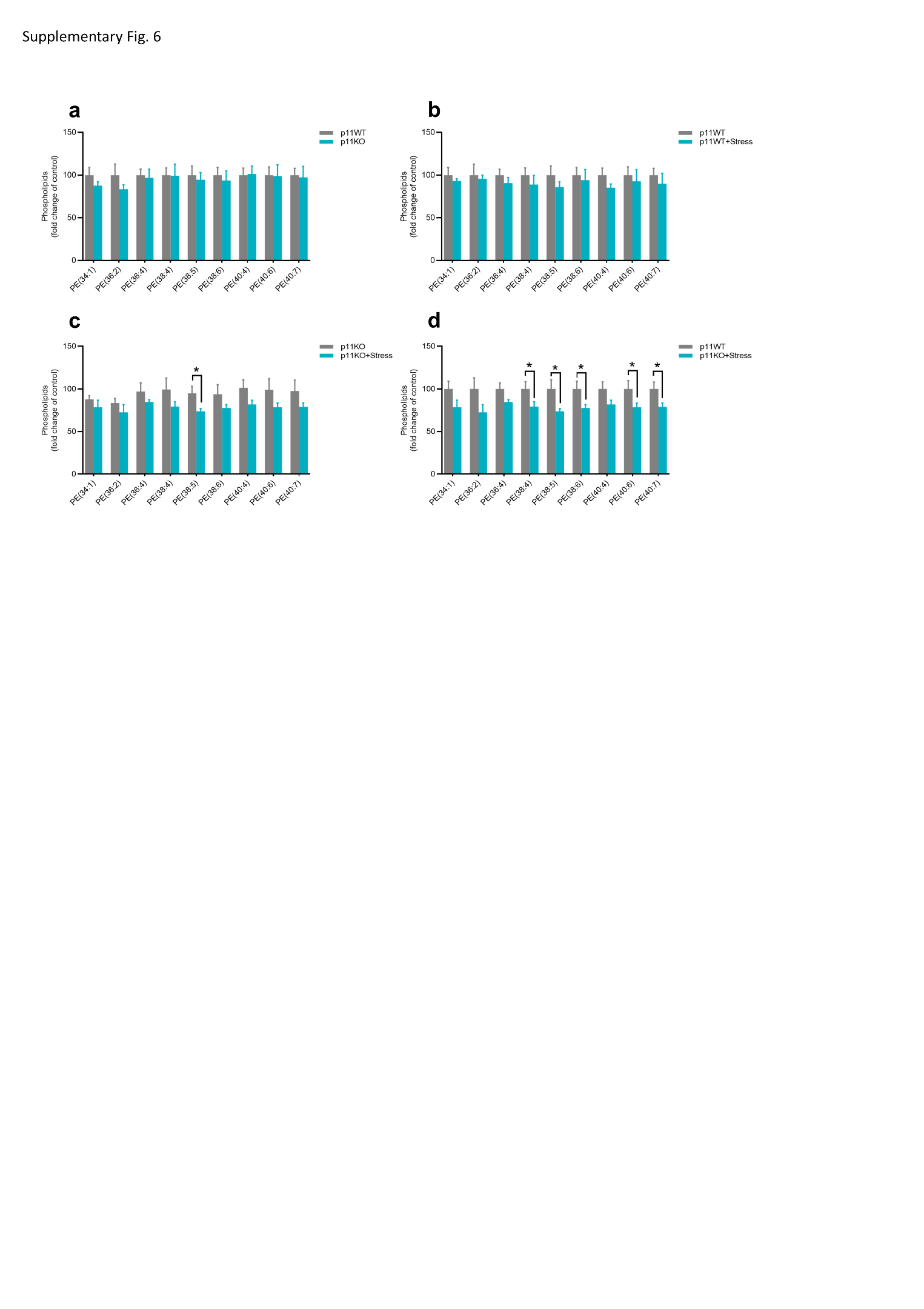


**Supplementary Fig. 7.** Altered levels of phosphatidylethanolamine (PE) species in the nucleus accumbens induced by p11 loss and stress challenge. Bar graph showing the quantification of the peaks of PE(34:1), PE(36:2), PE(36:4), PE(38:4), PE(38:5), PE(38:6), PE(40:4), PE(40:6), and PE(40:7) in the nucleus accumbens of (**a**) p11KO mice compared to p11WT mice, (**b**) p11WT+stress mice compared to p11WT mice, (**c**) p11KO+stress mice compared to p11KO mice, and (**d**) p11KO+stress mice compared to p11WT mice. Values are expressed as means ± S.E.M. (*n* = 5). **p* < 0.05 (Student’s *t* test).


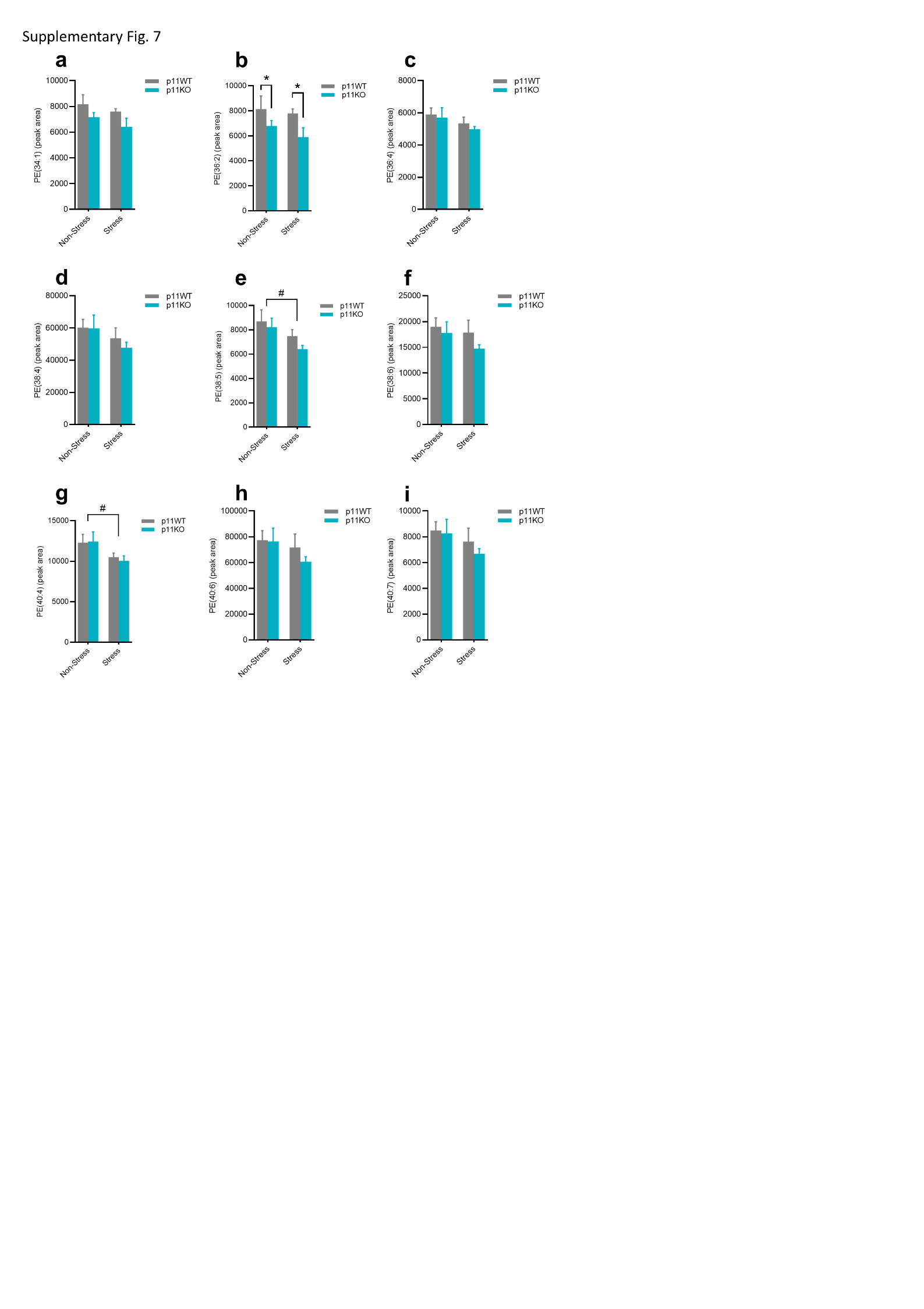


**Supplementary Fig. 8.** p11 loss and stress induce alterations in the levels of phosphatidylethanolamine (PE) species in the nucleus accumbens. Bar graph showing the quantification of the peaks of (**a**) PE(34:1), (**b**) PE(36:2), (**c**) PE(36:4), (**d**) PE(38:4), (**e**) PE(38:5), (**f**) PE(38:6), (**g**) PE(40:4), (**h**) PE(40:6), and (**i**) PE(40:7) in the nucleus accumbens of non-stressed or stressed p11WT or p11KO mice. Values are expressed as means ± S.E.M. (*n* = 5). **p* < 0.05 compared with the p11WT group (i.e., a significant main effect of genotype, two-way ANOVA). #*p* < 0.05 compared with the non-stressed groups (i.e., a significant main effect of stress, two-way ANOVA).


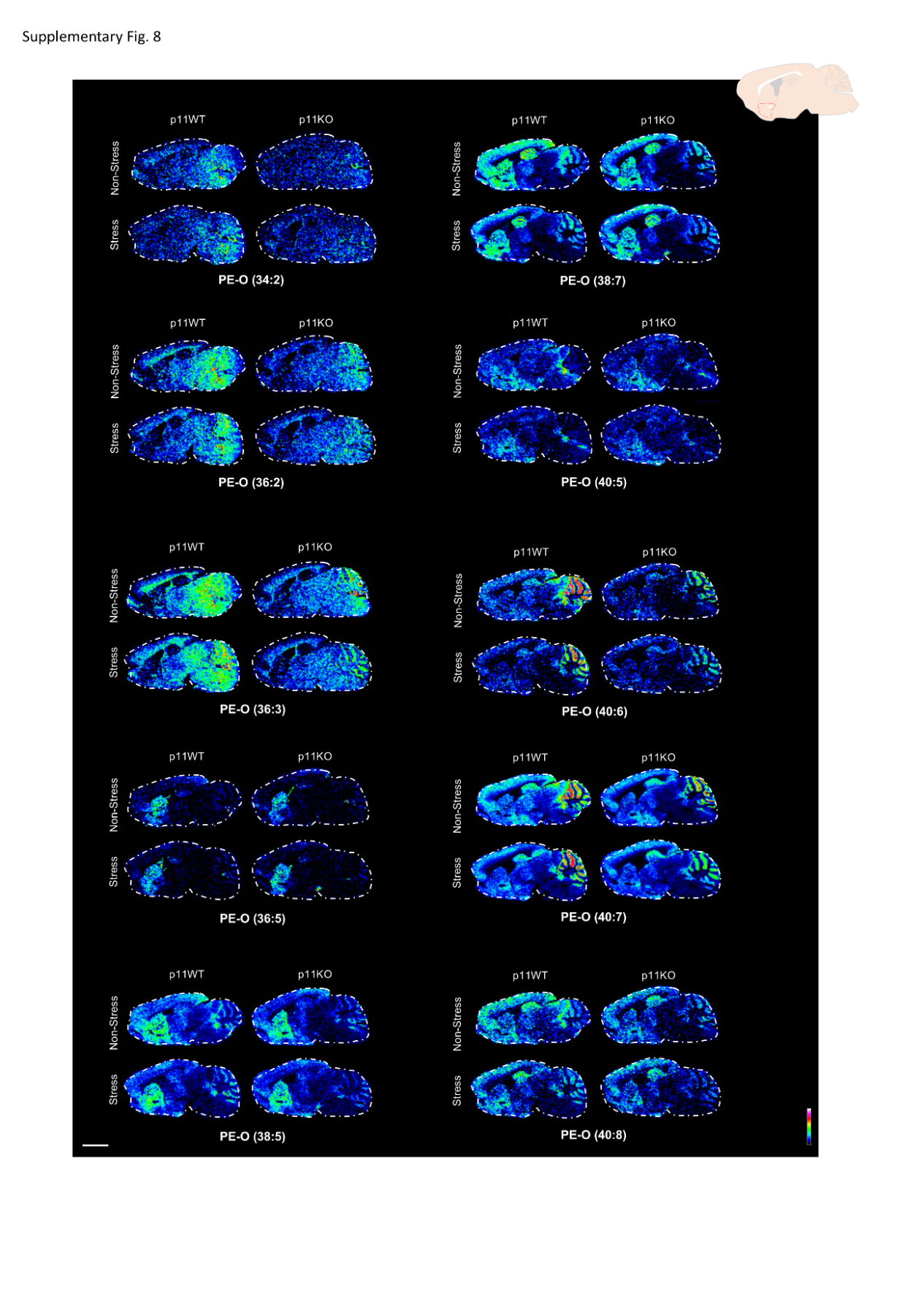


**Supplementary Fig. 9.** Effects of p11 deficiency and stress on ether phosphatidylethanolamine (PE-O) species levels. Representative ion images of PE-O(34:2), PE-O(36:2), PE-O(36:3), PE-O(36:5), PE-O(38:5), PE-O(38:7), PE-O(40:5), PE-O(40:6), PE-O(40:7), and PE-O(40:8) in the nucleus accumbens of stressed or non-stressed p11WT or p11KO mice. MALDI-MSI ion images were presented as RMS normalized and acquired at a lateral resolution of 150 µm. Data are shown using a rainbow scale (representing ion intensity scale) for best visualization.


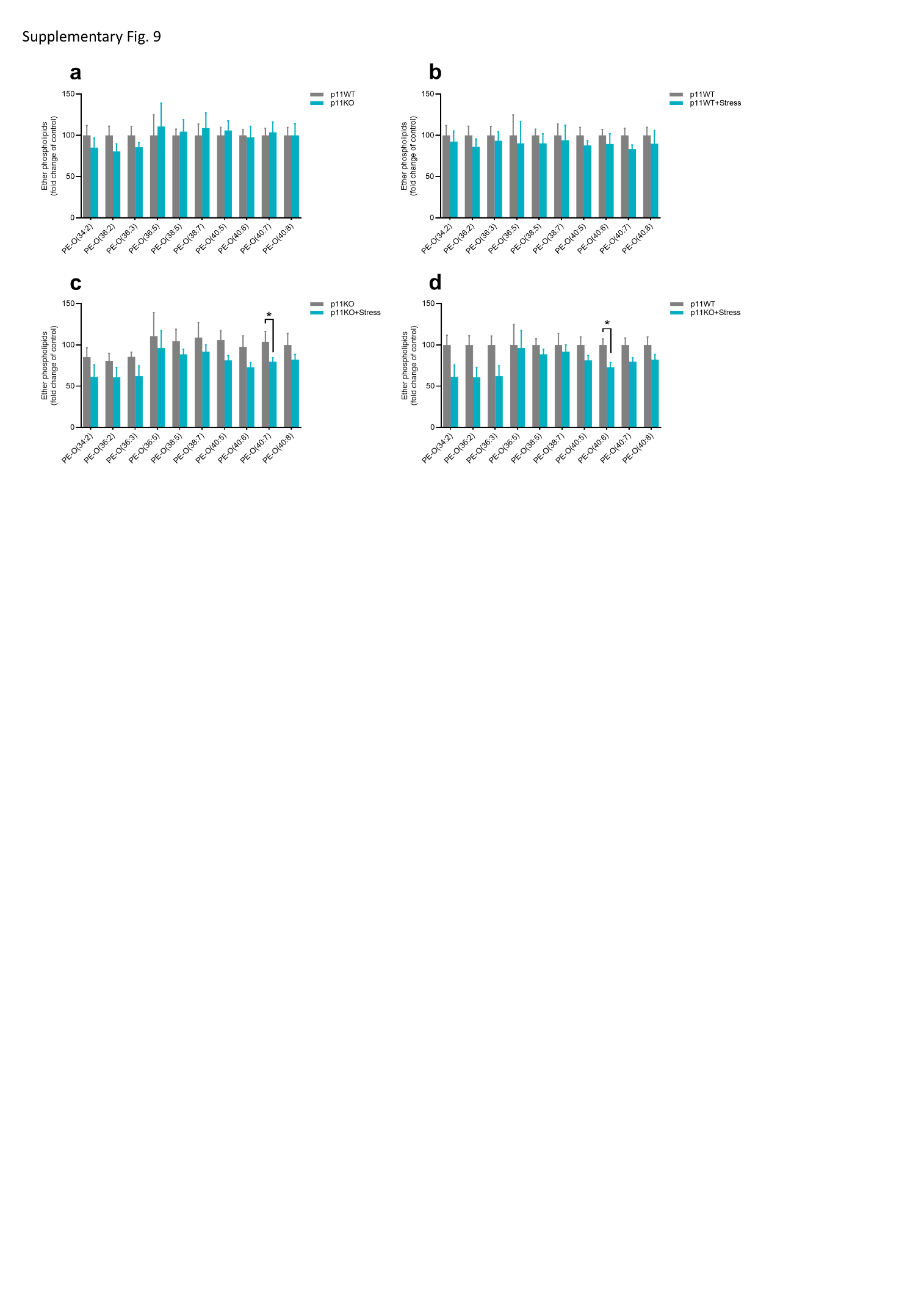


**Supplementary Fig. 10.** p11 loss and stress induce alterations in the levels of ether phosphatidylethanolamine (PE) species in the nucleus accumbens. Bar graph showing the quantification of the peaks of PE-O(34:2), PE-O(36:2), PE-O(36:3), PE-O(36:5), PE-O(38:5), PE-O(38:7), PE-O(40:5), PE-O(40:6), PE-O(40:7), and PE-O(40:8) in the nucleus accumbens of (**a**) p11KO mice compared to p11WT mice, (**b**) p11WT+stress mice compared to p11WT mice, (**c**) p11KO+stress mice compared to p11KO mice, and (**d**) p11KO+stress mice compared to p11WT mice. Values are expressed as means ± S.E.M. (*n* = 5). **p* < 0.05 (Student’s *t* test).


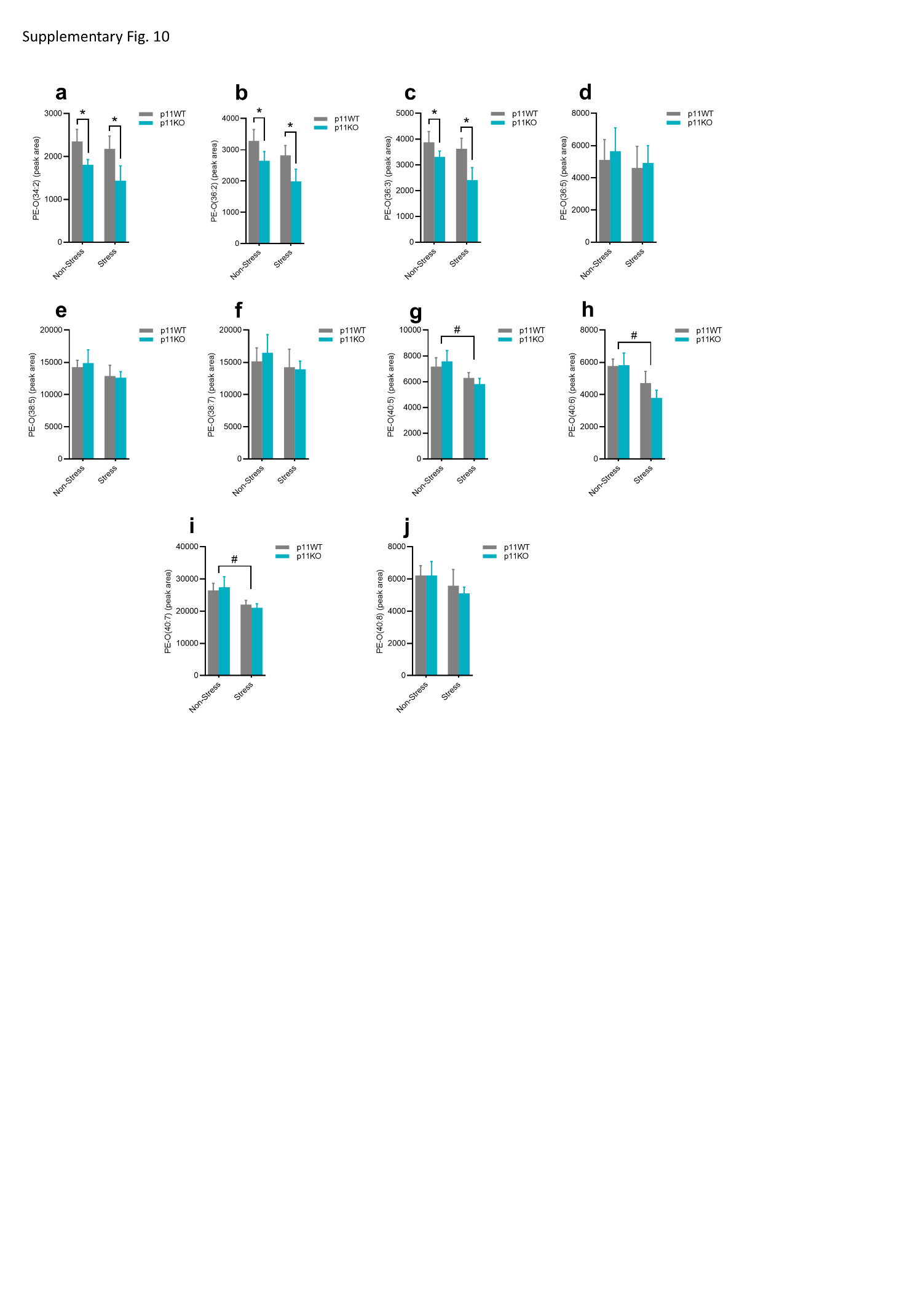


**Supplementary Fig. 11.** Altered levels of ether phosphatidylethanolamine (PE-O) species in the nucleus accumbens elicited by p11 deficiency or stress. Bar graph showing the quantification of the peaks of (**a**) PE-O(34:2), (**b**) PE-O(36:2), (**c**) PE-O(36:3), (**d**) PE-O(36:5), (**e**) PE-O(38:5), (**f**) PE-O(38:7), (**g**) PE-O(40:5), (**h**) PE-O(40:6), (**i**) PE-O(40:7), and (**j**) PE-O(40:8) species in the nucleus accumbens of non-stressed or stressed p11WT or p11KO mice. Values are expressed as means ± S.E.M. (*n* = 5). **p* < 0.05 compared with the p11WT group (i.e., a significant main effect of genotype, two-way ANOVA). #*p* < 0.05 compared with the non-stressed groups (i.e., a significant main effect of stress, two-way ANOVA).


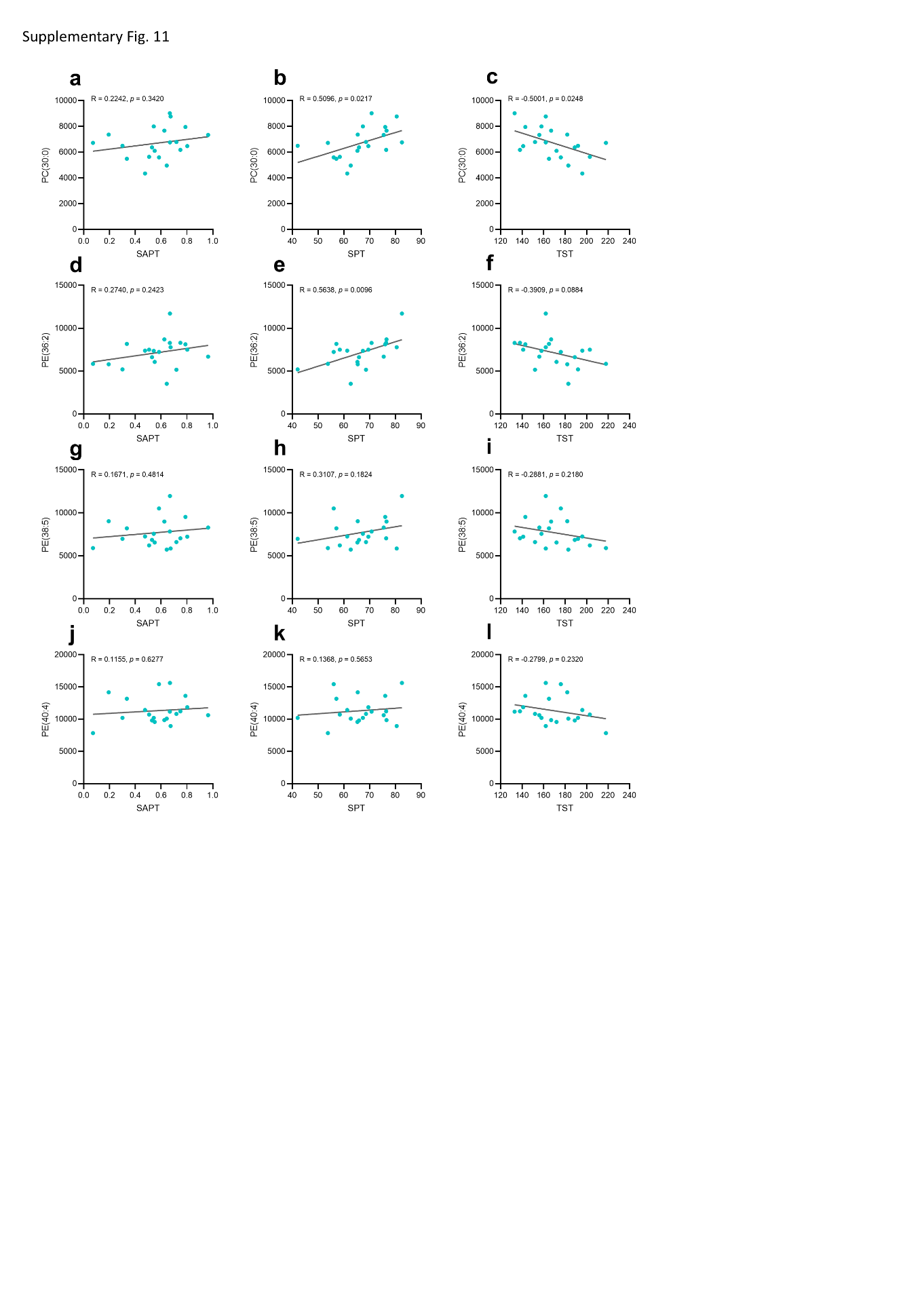


**Supplementary Fig. 12.** Altered levels of phospholipid species in the nucleus accumbens correlate with the depression-like phenotype induced by p11 deficiency and stress. Pearson´s correlation coefficient between PC(30:0), PE(36:2), PE(38:5), and PE(40:4) levels and social affective preference test (**a**, **d**, **g**, and **j**), sucrose preference test (**b**, **e**, **h**, and **k**), and tail suspension test (**c**, **f**, **i**, and **l**).


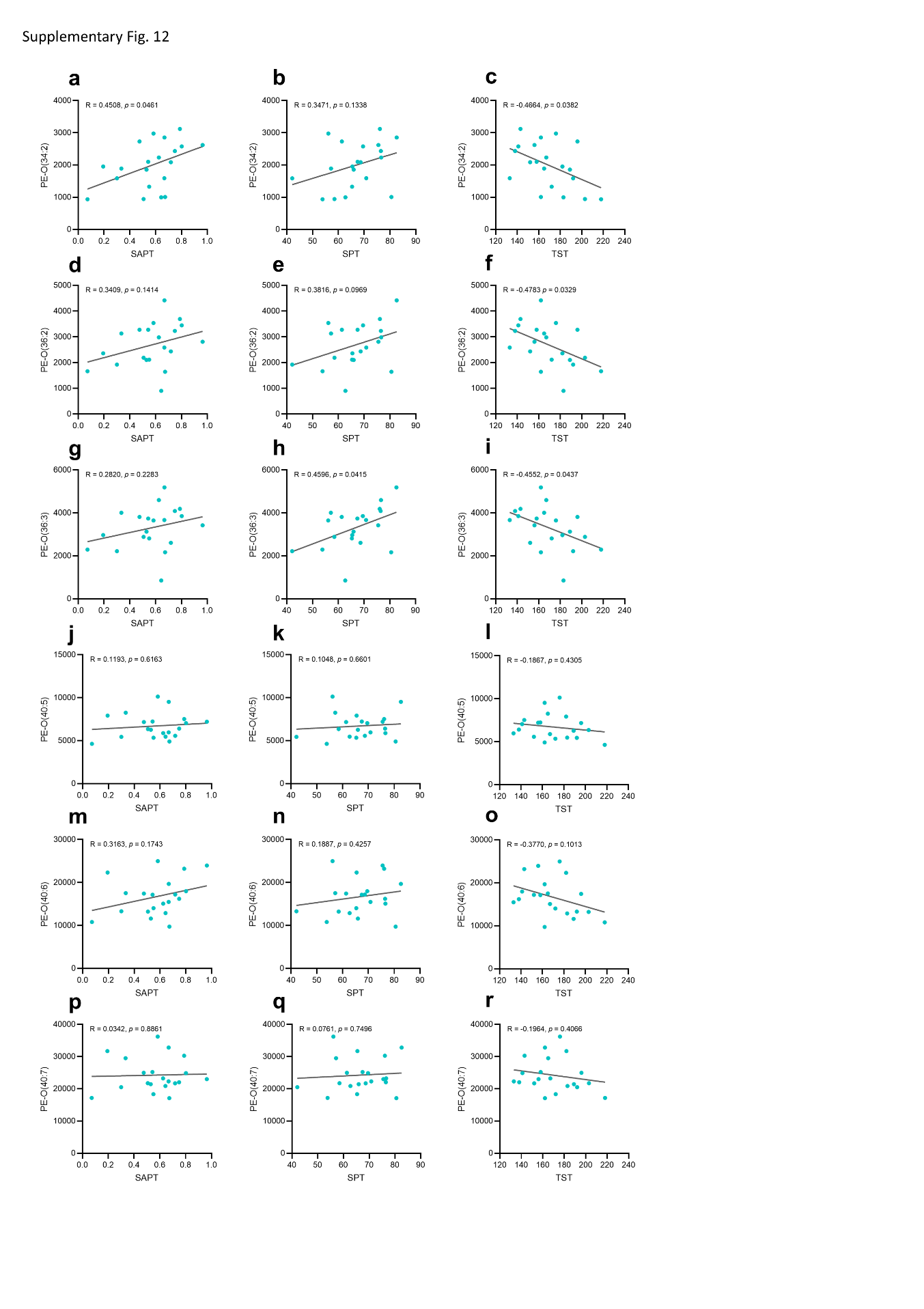


**Supplementary Fig. 13.** Levels of ether phospholipid species in the nucleus accumbens correlate with the depression-like phenotype. Pearson´s correlation coefficient between PE-O(34:2), PE-O(36:2), PE-O(36:3), PE(40:5), PE(40:6), and PE(40:7) levels and social affective preference test (**a**, **d**, **g**, **j**, **m**, and **p**), sucrose preference test (**b**, **e**, **h**, **k**, **n**, and **q**), and tail suspension test (**c**, **f**, **i**, **l**, **o**, and **r**).


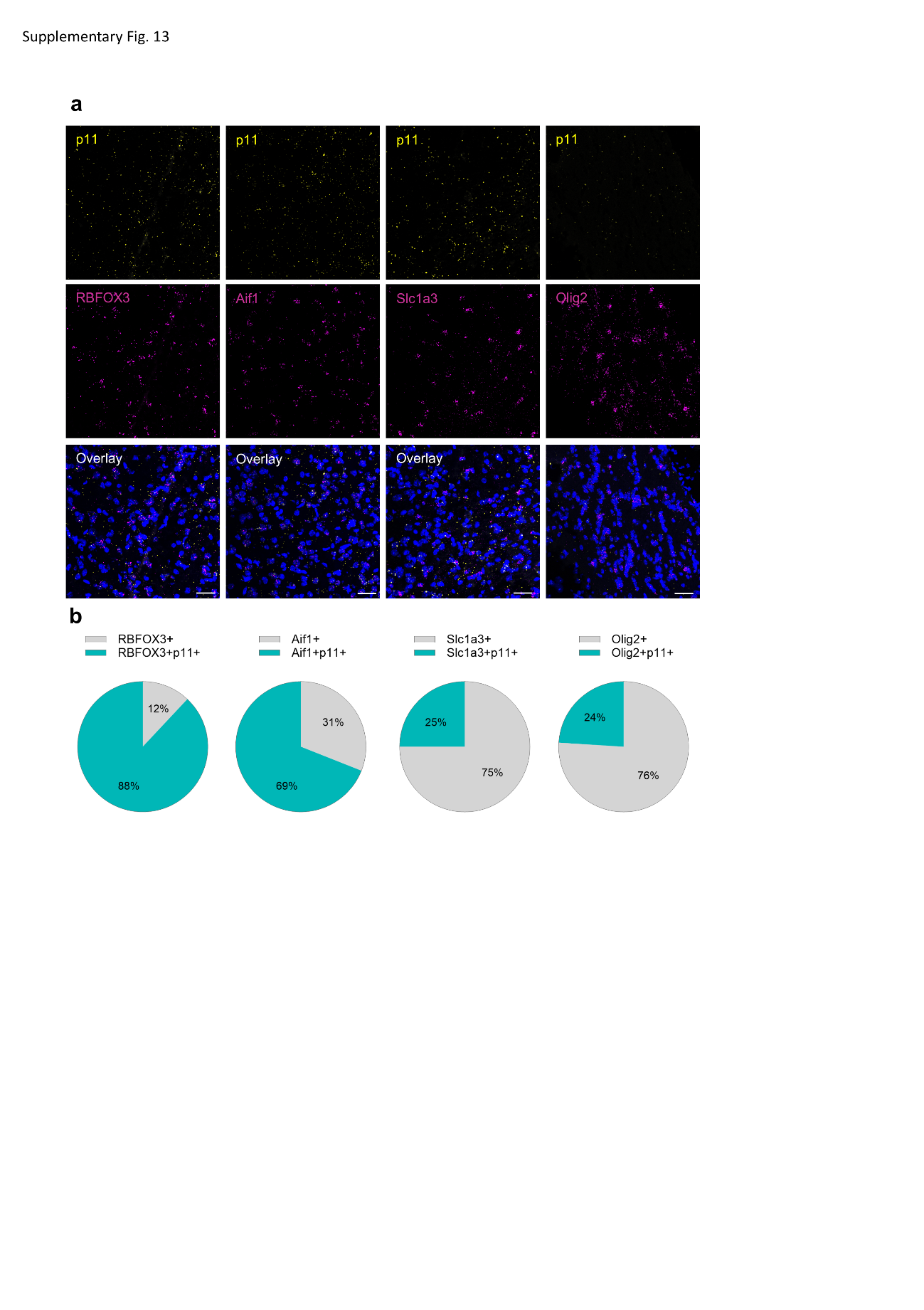


**Supplementary Fig. 14.** p11 is mostly enriched in neuronal cells. (**a**) Fluorescence in situ hybridization (RNAscope) images of p11 (yellow) mRNA levels and RBFOX3 (magenta, neuronal marker), Aif1 (magenta, microglia marker), and Slc1a3 (magenta, astrocyte marker) mRNA in the nucleus accumbens. Representative RNAscope images of p11 (yellow) mRNA levels and Olig2 (magenta, oligodendrocyte marker) in the corpus callosum. Scale bars: 100 µm. (**b**) Pie charts illustrate the percentage of p11⁺ cells co-expressing RBFOX3⁺ (neuronal), Aif1⁺ (microglial), Slc1a3⁺ (astrocytic), or Olig2⁺ (oligodendrocytic) markers.


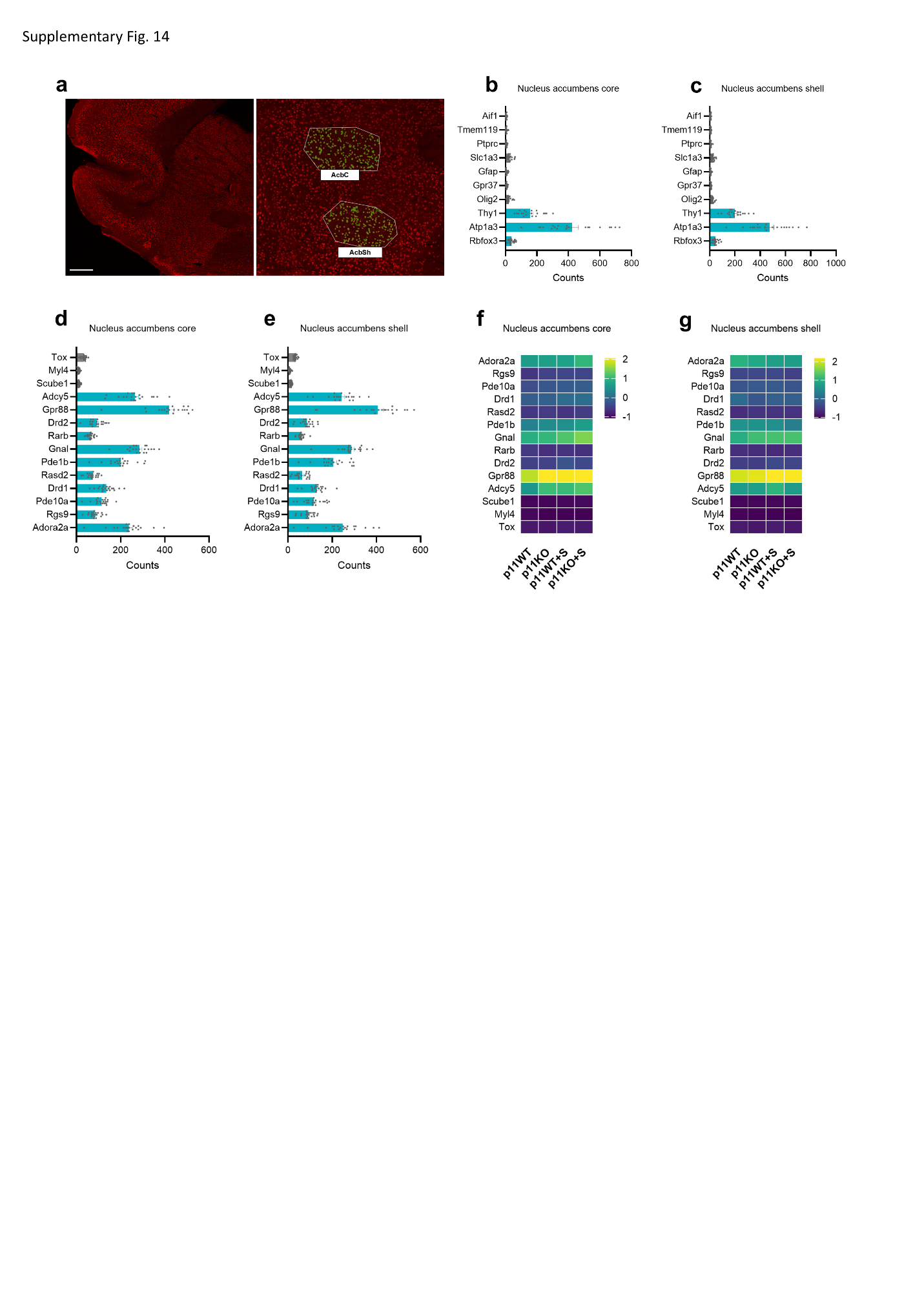


**Supplementary Fig. 15.** Neuron-specific transcriptome signature in the nucleus accumbens of non-stressed and stressed p11WT and p11KO mice. **a** Representative NeuN (red) staining image showing cell segmentation (green) in the nucleus accumbens core and shell. Scale bars: 500 µm. Bar graph showing selected transcript counts of genes enriched in neurons (Rbofox3, Atp1a3, Thy1), oligodendrocytes (Olig2 and Gpr37), astrocytes (Gfap and Slc1a3), and microglia (Ptprc, Tmem119, and Aif1) according to AGEA Fine Structure Search of the Allen brain atlas in the nucleus accumbens core (**b**) and shell (**c**). Bar graph showing high transcript counts of nucleus accumbens-enriched genes (Adora2a, Rgs9, Pde10a, Drd1, Ras2, Pde1b, Gnal, Rarb, Drd1, Gpr88, and Adcy5), but not prefrontal cortex-enriched genes (Scube1, Myl4, and Tox), in the nucleus accumbens core (**d**) and shell (**e**). Heat map of nucleus accumbens-enriched genes and prefrontal cortex-enriched genes in the nucleus accumbens core (**e**) and shell (**f**) of non-stressed or stressed p11WT or p11KO mice. Each square represents the z-score mean of the corresponding target with the value normalized across the groups.


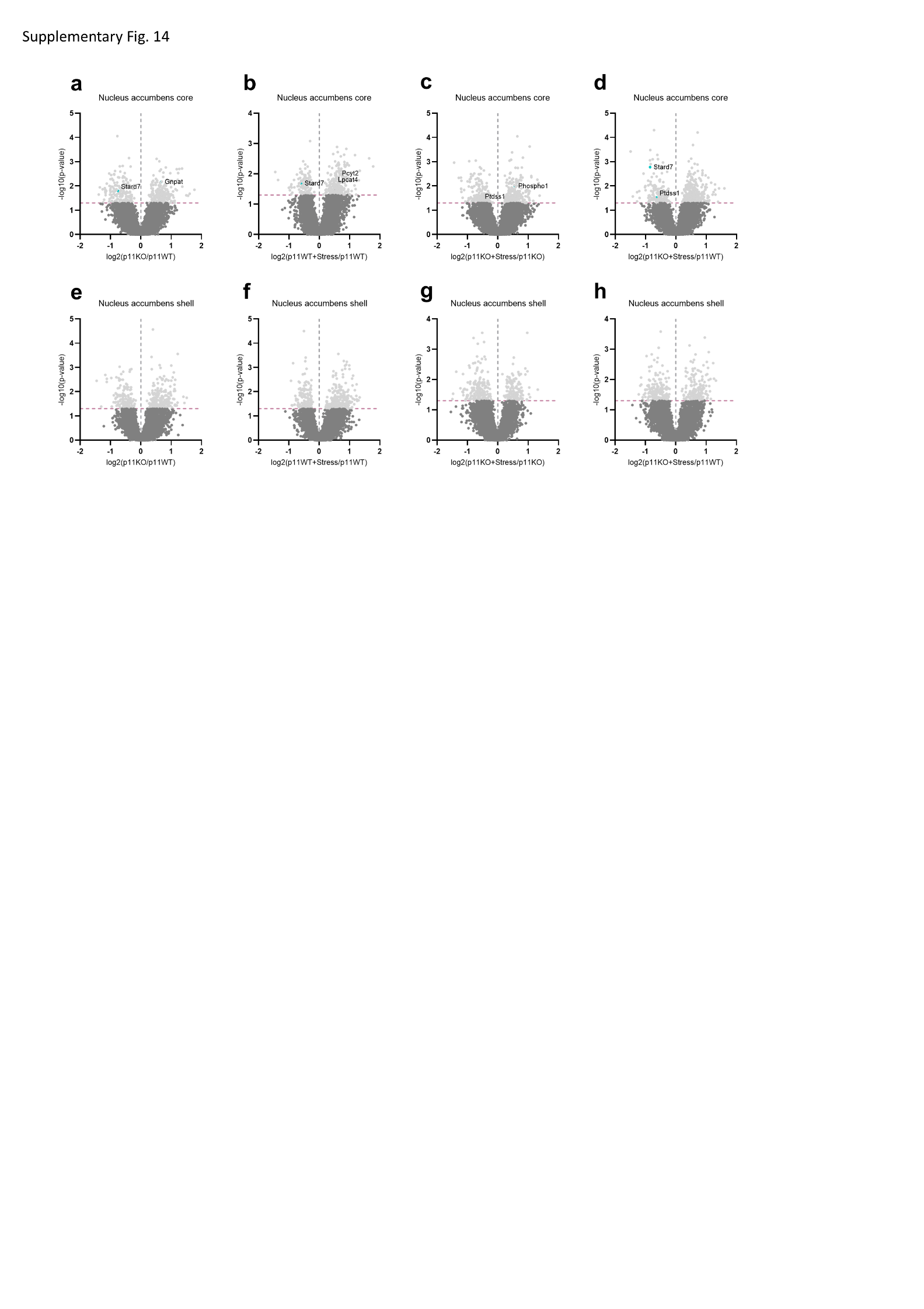


**Supplementary Fig. 16.** Neuron-specific transcriptome signature in the nucleus accumbens of non-stressed and stressed p11WT and p11KO mice. Volcano plots showing the indicated fold differences and -log*10* (non-adjusted *p* value) of all transcript in the nucleus accumbens core and shell of (**a** and **e**) p11KO mice compared to p11WT mice, (**b** and **f**) p11WT+stress mice compared to p11WT mice, (**c** and **g**) p11KO+stress mice compared to p11KO mice, and (**d** and **h**) p11KO+stress mice compared to p11WT mice. The pink dashed line represents FDR (*p*) = 5%. Each dot depicts one transcript.


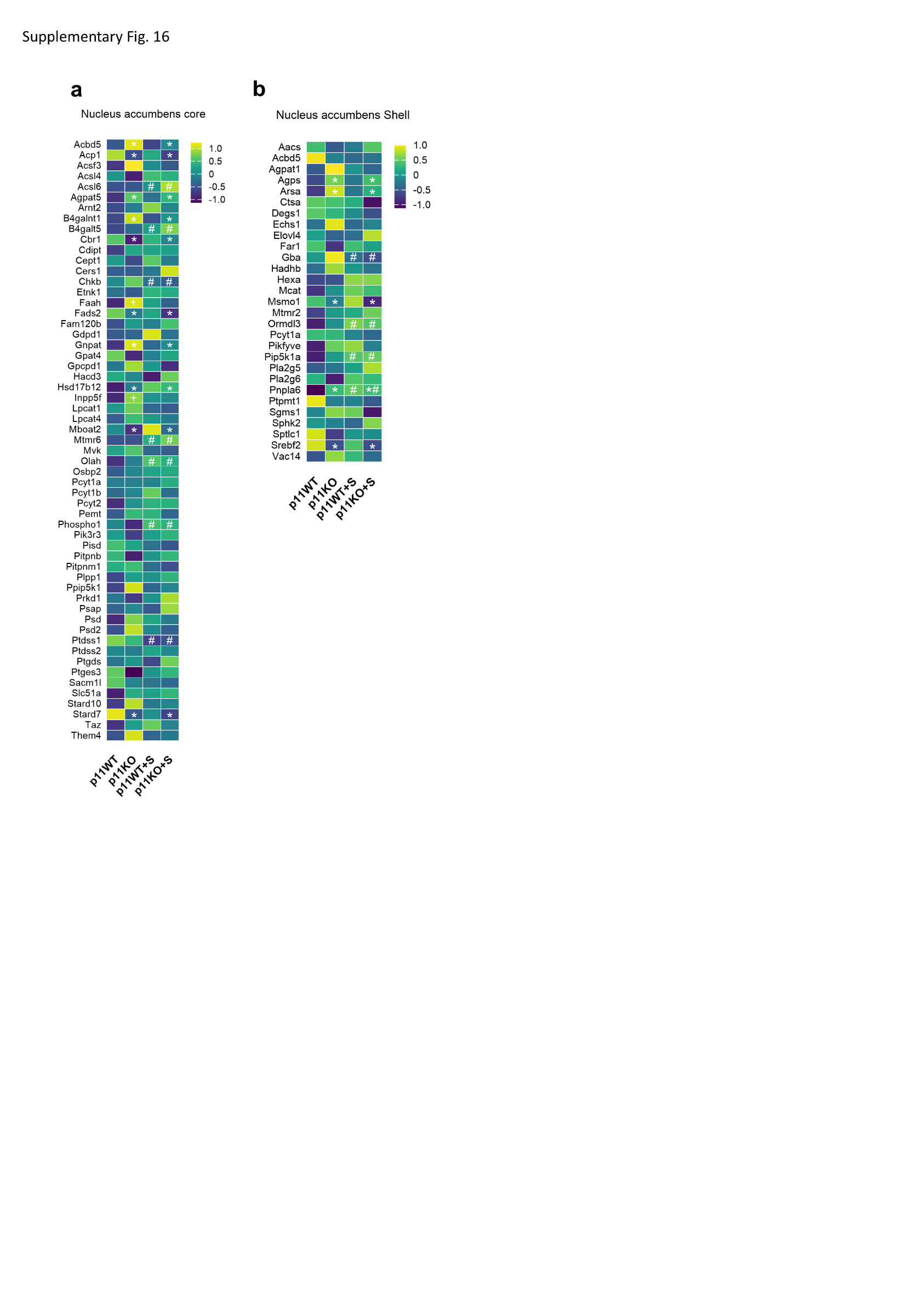


**Supplementary Fig. 17.** Transcriptome signature in the nucleus accumbens core and shell reveals alterations in lipid metabolism-related targets. Heat map of targets involved in PE metabolism in the nucleus accumbens core (**e**) and shell (**f**) of non-stressed or stressed p11WT or p11KO mice. Each square represents the z-score mean of the corresponding protein with the value normalized across the groups (*n* = 5). **p* < 0.05 compared with the p11WT group (i.e., a significant main effect of genotype, two-way ANOVA); #*p* < 0.05 compared with non-stressed mice (i.e., a significant main effect of stress protocol, two-way ANOVA). +*p* < 0.05 compared with the p11WT group (two-way ANOVA followed by Tukey’s post hoc test).


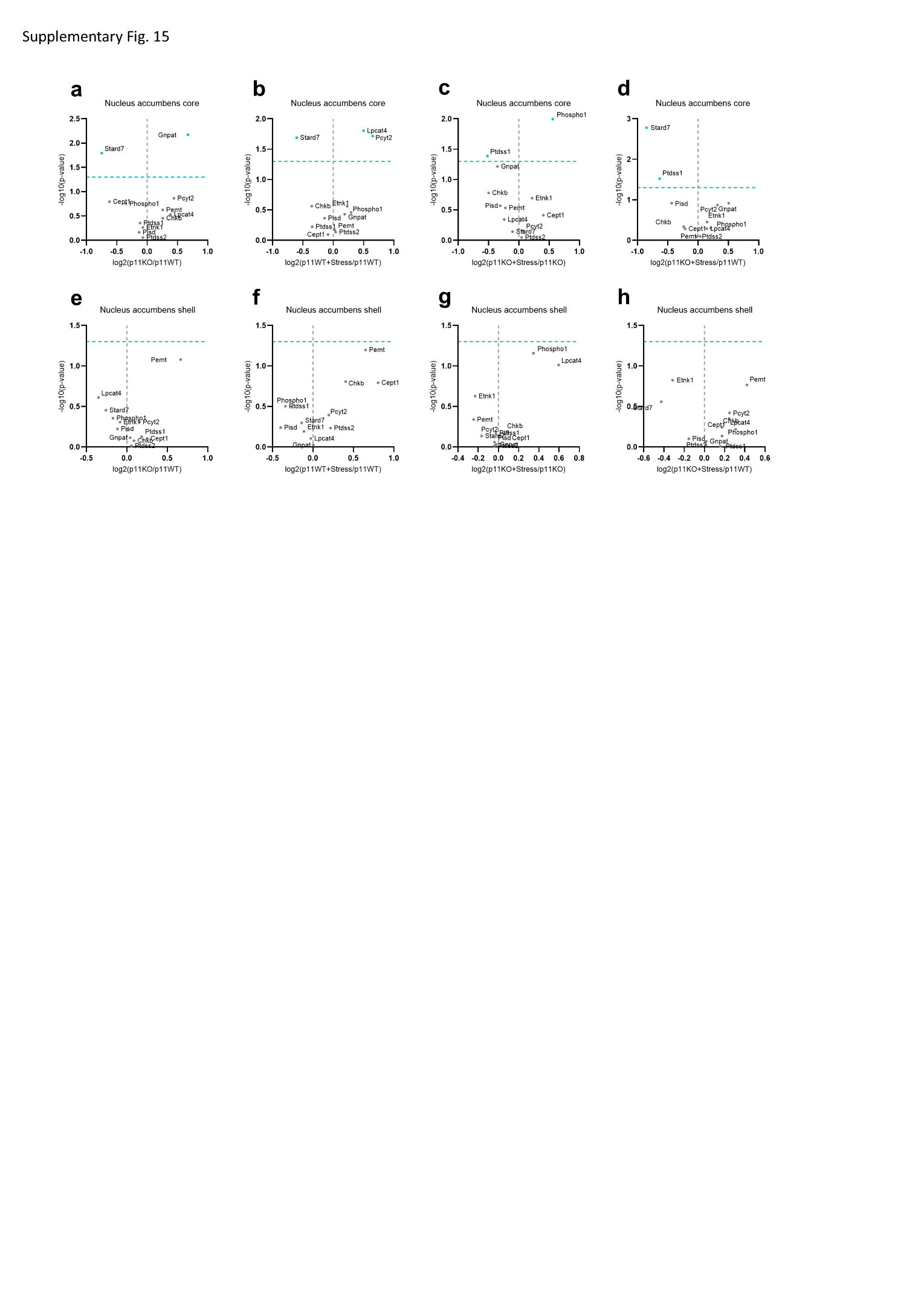


**Supplementary Fig. 18.** Neuron-specific transcriptome signature in the nucleus accumbens reveals alterations in molecular targets related to phosphatidylethanolamine (PE) metabolism. Volcano plots showing the indicated fold differences and -log*10* (non-adjusted *p* value) of PEs-related transcript in the nucleus accumbens core and shell of (**a** and **e**) p11KO mice compared to p11WT mice, (**b** and **f**) p11WT+stress mice compared to p11WT mice, (**c** and **g**) p11KO+stress mice compared to p11KO mice, and (**d** and **h**) p11KO+stress mice compared to p11WT mice. The green dashed line represents FDR (*p*) = 5%. Each dot depicts one transcript.


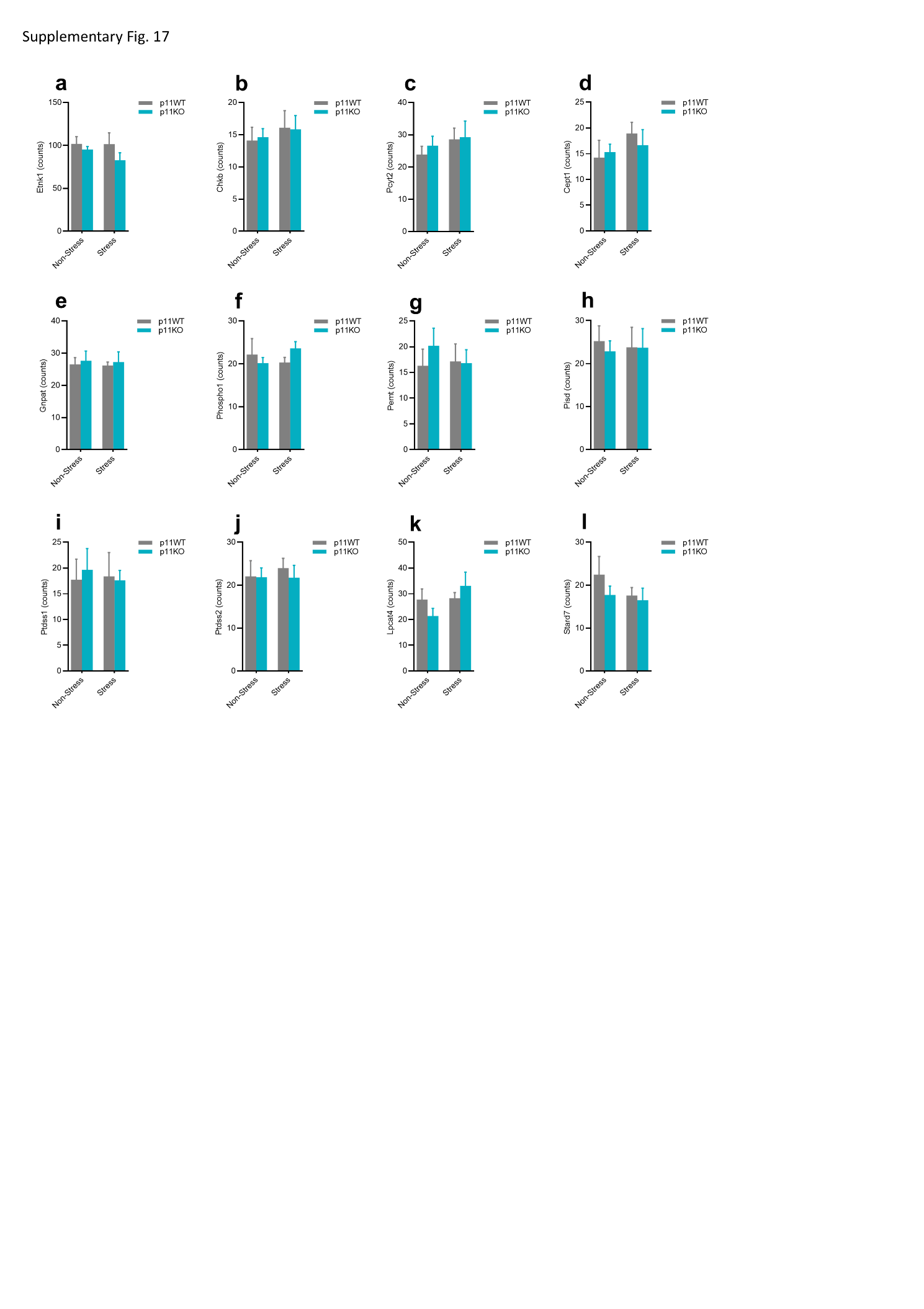


**Supplementary Fig. 19.** Effects of p11 loss and stress on the levels of phosphatidylethanolamine (PE) metabolism-related targets in the nucleus accumbens shell. Bar graph showing the quantification of Etnk1 (**a**), Chkb (**b**), Pcyt2 (**c**), Cept1 (**d**), Gnpat (**e**), Phospho1 (**f**), Pemt (**g**), Pisd (**h**), Ptdss1 (**i**), Ptdss2 (**j**), Lpcat4 (**k**), and Stard7 (**l**). Values are expressed as means ± S.E.M. (*n* = 5, two-way ANOVA).


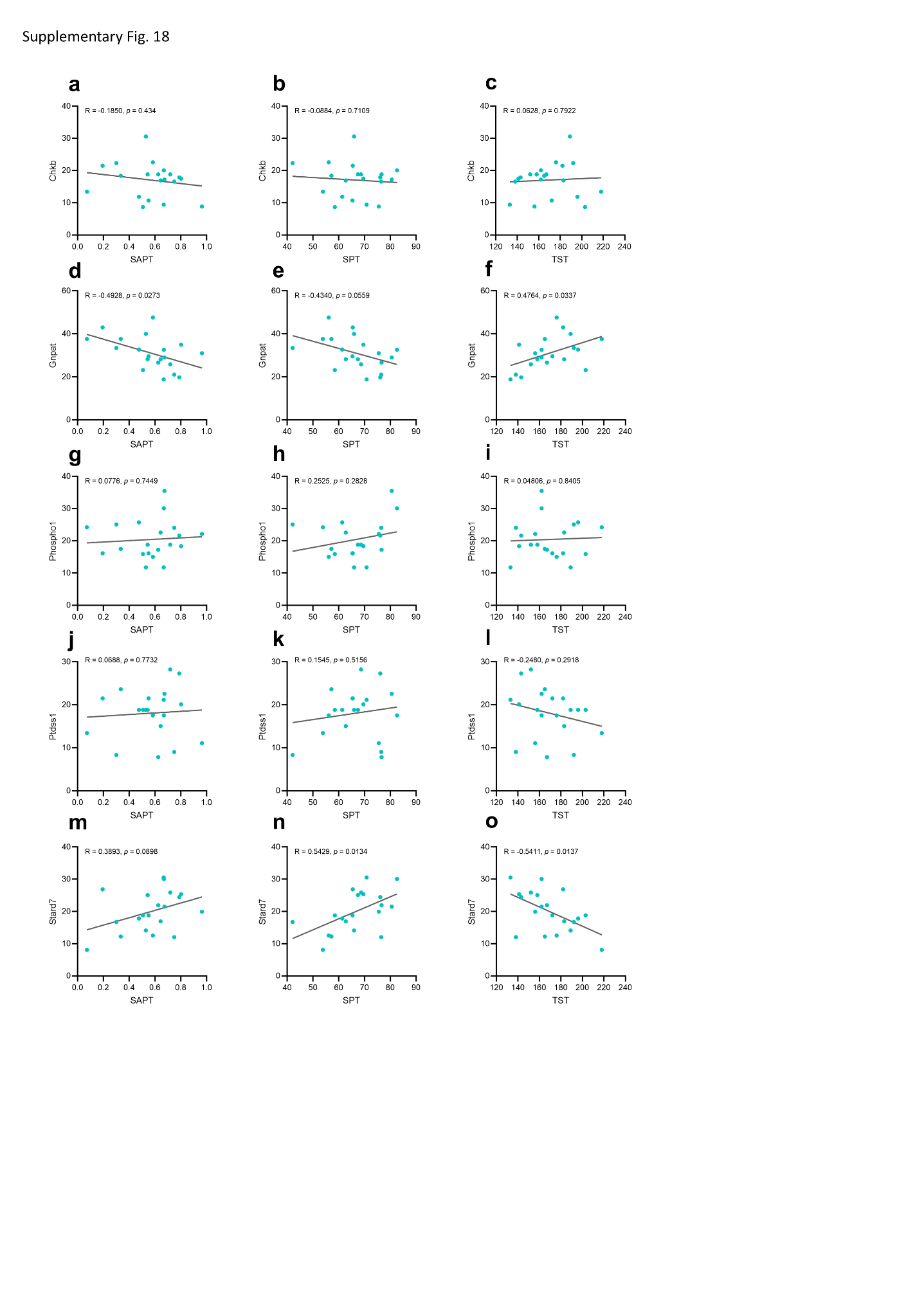


**Supplementary Fig. 20.** Phosphatidylethanolamine synthesis pathway-related targets correlate with stress-induced maladaptive responses. Pearson´s correlation coefficient between Chkb, Gnpat, Phospho1, Ptdss1, and Stard7 levels and social affective preference test (**a**, **d**, **g**, **j**, and **m**, ), sucrose preference test (**b**, **e**, **h**, **k**, and **n**), and tail suspension test (**c**, **f**, **i**, **l**, and **o**).


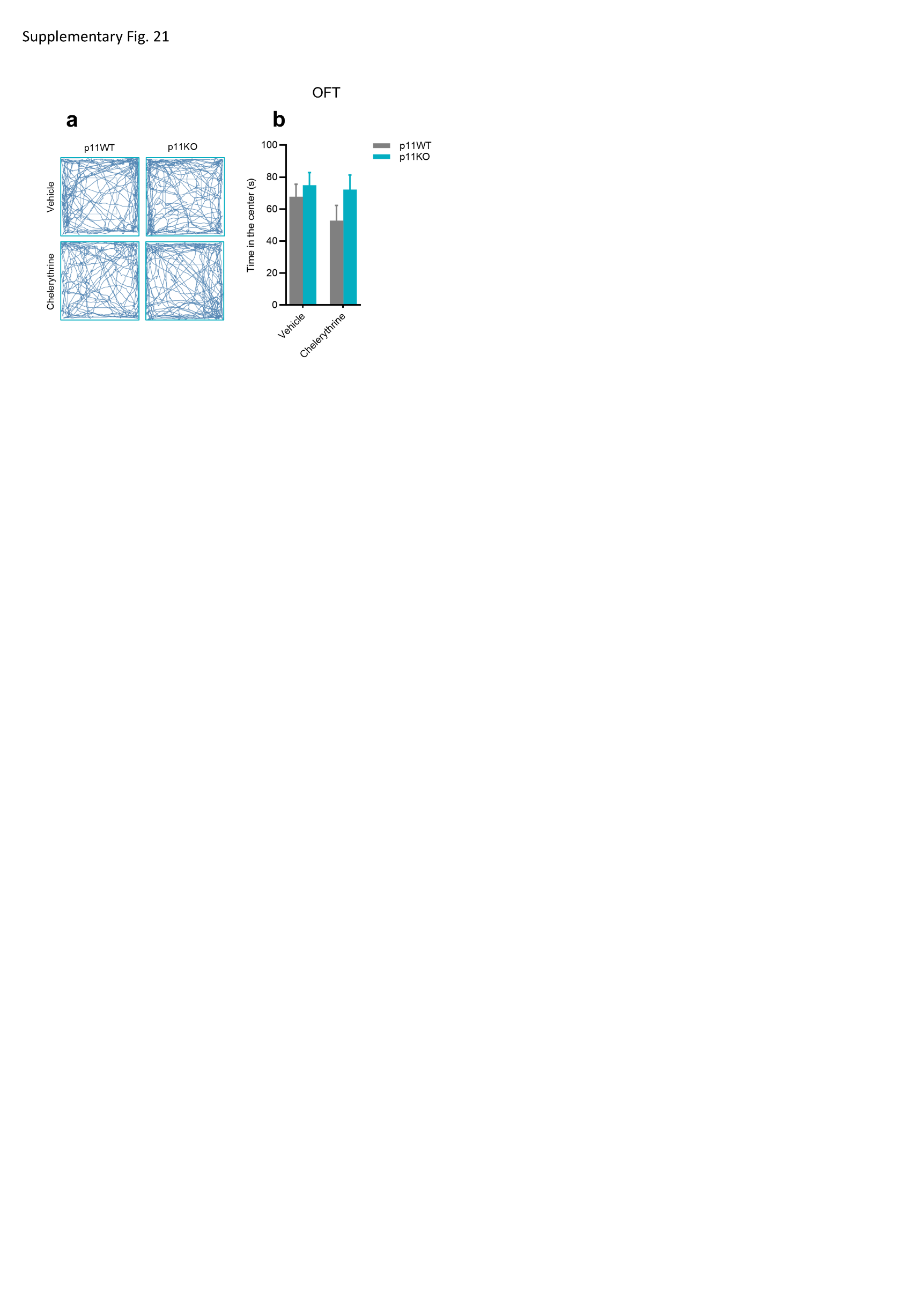


**Supplementary Fig. 21.** Behavioral effects of p11 deficiency and stress in the open-field test. **a** Representative track plots across all the experimental groups. **b** All experimental groups presented a similar distance time spent in the center in the open-field test (OFT). Values are expressed as means ± S.E.M. (*n* = 8). Two-way ANOVA analysis.


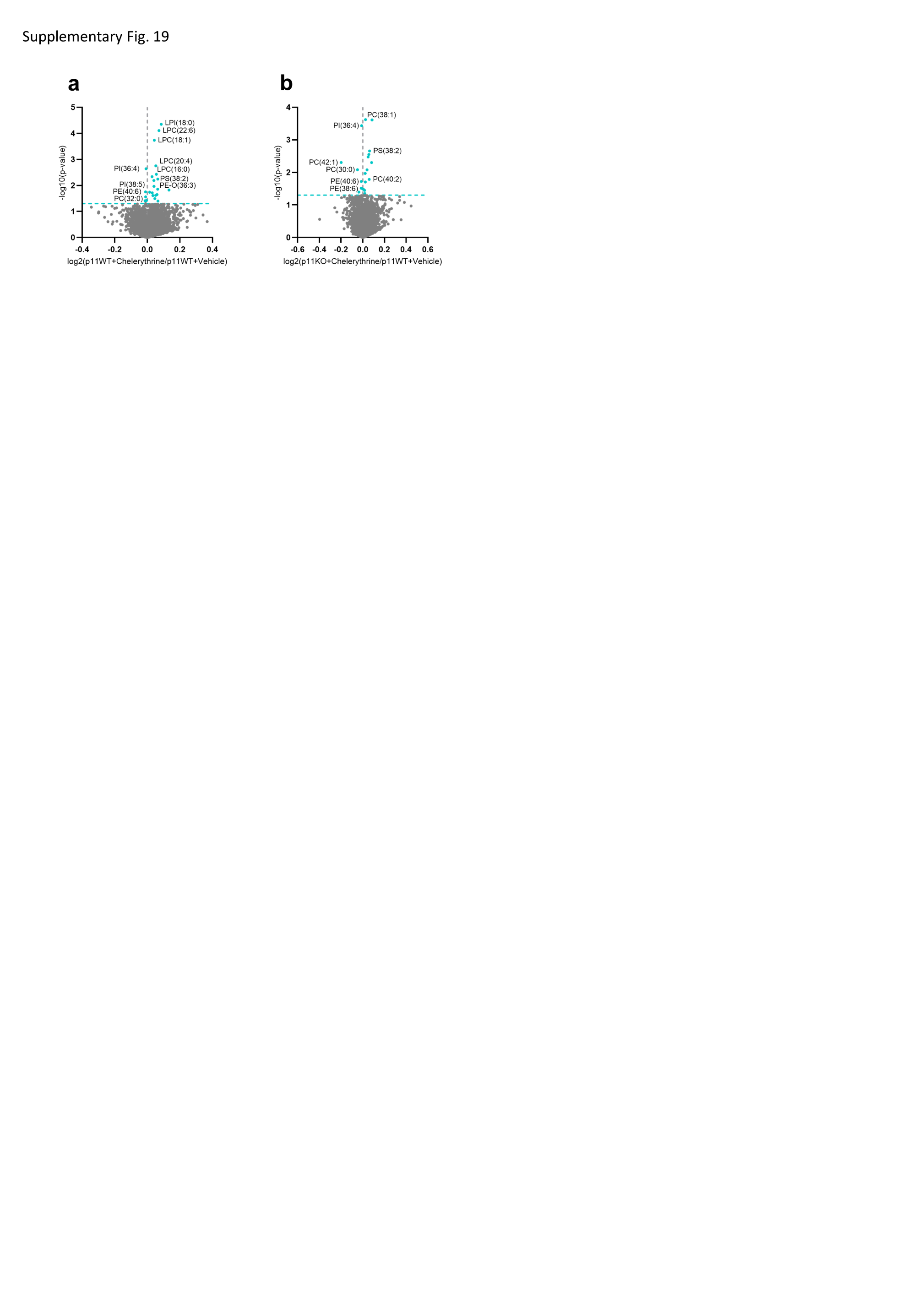


**Supplementary Fig. 22.** Effects of chelerythrine on phospholipid levels in the nucleus accumbens. Volcano plots showing the indicated fold differences and -log*10* (non-adjusted *p* value) of phospholipids, in particular phosphatidylcholine (PC), phosphatidylethanolamine (PE), ether phosphatidylethanolamine (PE-O), phosphatidylinositol (PI), and phosphatidylserine (PS) species in the nucleus accumbens of (**a**) chelerythrine-treated p11WT mice compared to vehicle-treated p11WT mice, (**b**) chelerythrine-treated p11KO mice compared to vehicle-treated p11WT mice. The green dashed line represents FDR (*p*) = 5%. Each dot depicts one lipid.

**
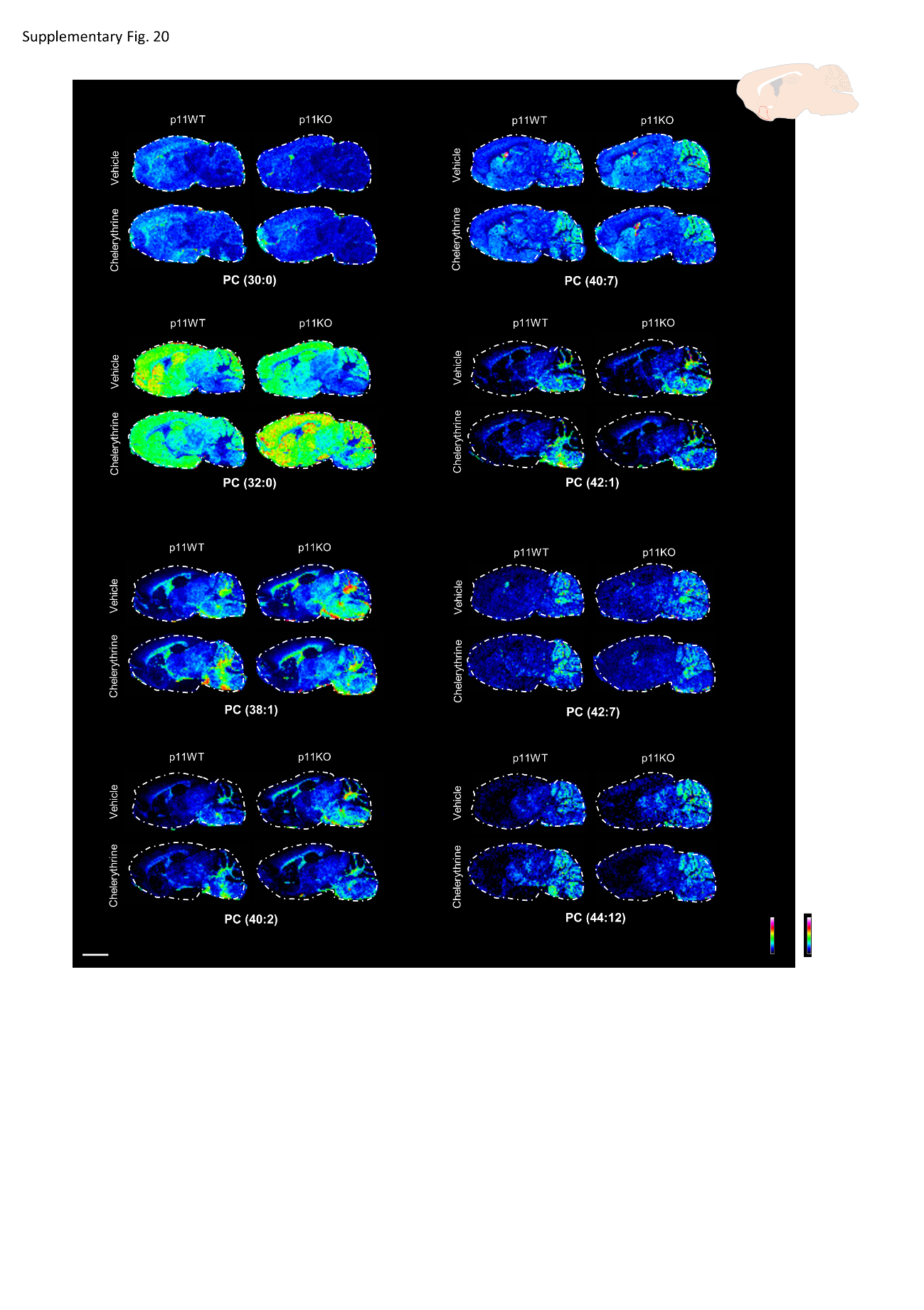
**

**Supplementary Fig. 23.** Effects of chelerythrine on phosphatidylcholine (PC) species levels in the nucleus accumbens. Representative ion images of PC(30:0), PC(32:0), PC(38:1), PC(40:2), PC(40:7), PC(42:1), PC(42:7), and PC(44:12) in the nucleus accumbens of p11WT or p11KO mice. MALDI-MSI ion images were presented as RMS normalized and acquired at a lateral resolution of 150 µm. Data are shown using a rainbow scale (representing ion intensity scale) for best visualization.

**
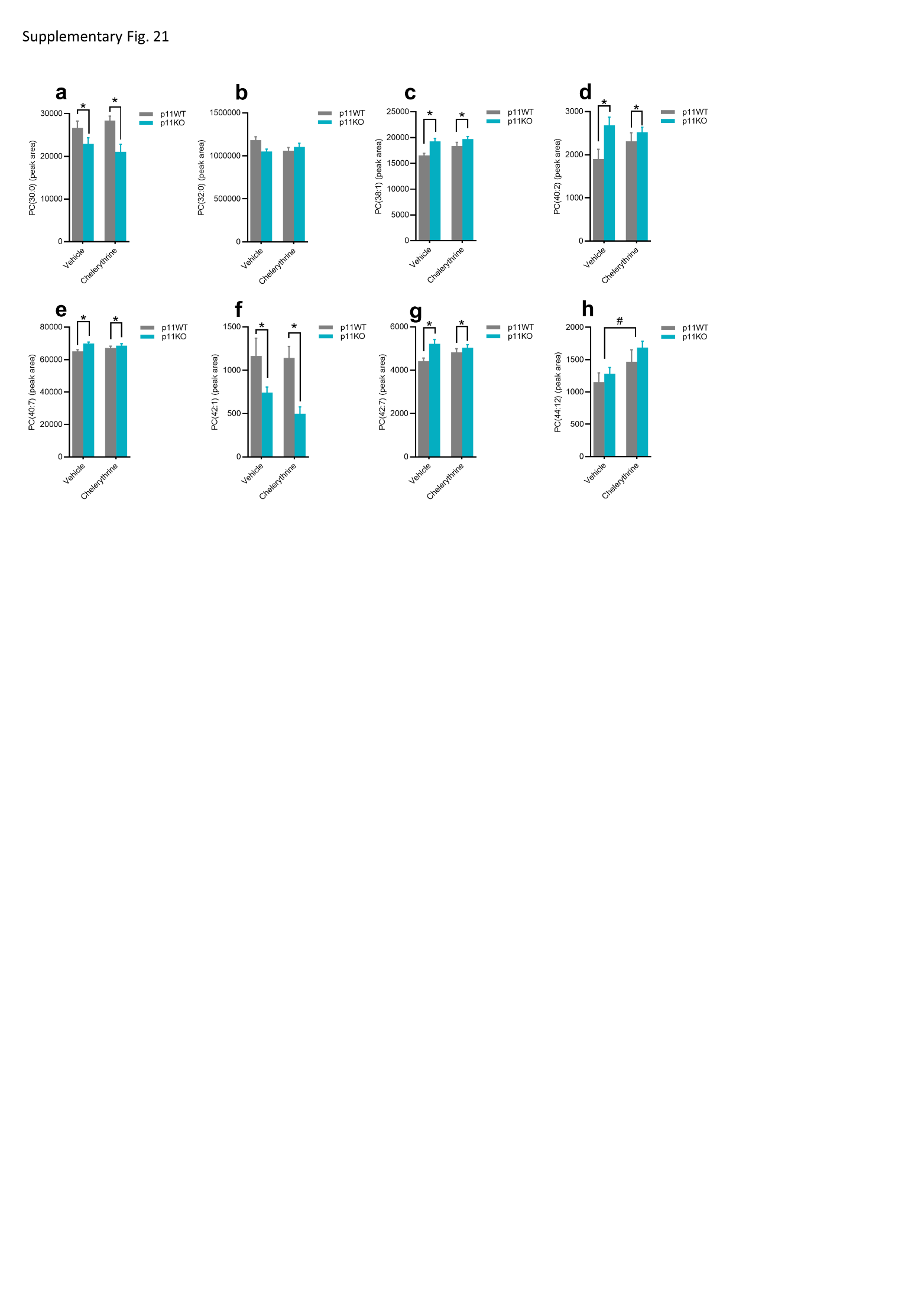
**

**Supplementary Fig. 24.** Altered levels of phosphatidylcholine (PC) species in the nucleus accumbens. Bar graph showing the quantification of the peaks of (**a**) PC(30:0), (**b**) PC(32:0), (**c**) PC(38:1), (**d**) PC(40:2), (**e**) PC(40:7), (**f**) PC(42:1), (**g**) PC(42:7), and (**h**) PC(44:12) in the nucleus accumbens of p11WT or p11KO mice. Values are expressed as means ± S.E.M. (*n* = 8). **p* < 0.05 compared with the p11WT group (i.e., a significant main effect of genotype, two-way ANOVA). #*p* < 0.05 compared with the vehicle-treated groups (i.e., a significant main effect of chelerythrine, two-way ANOVA).

**
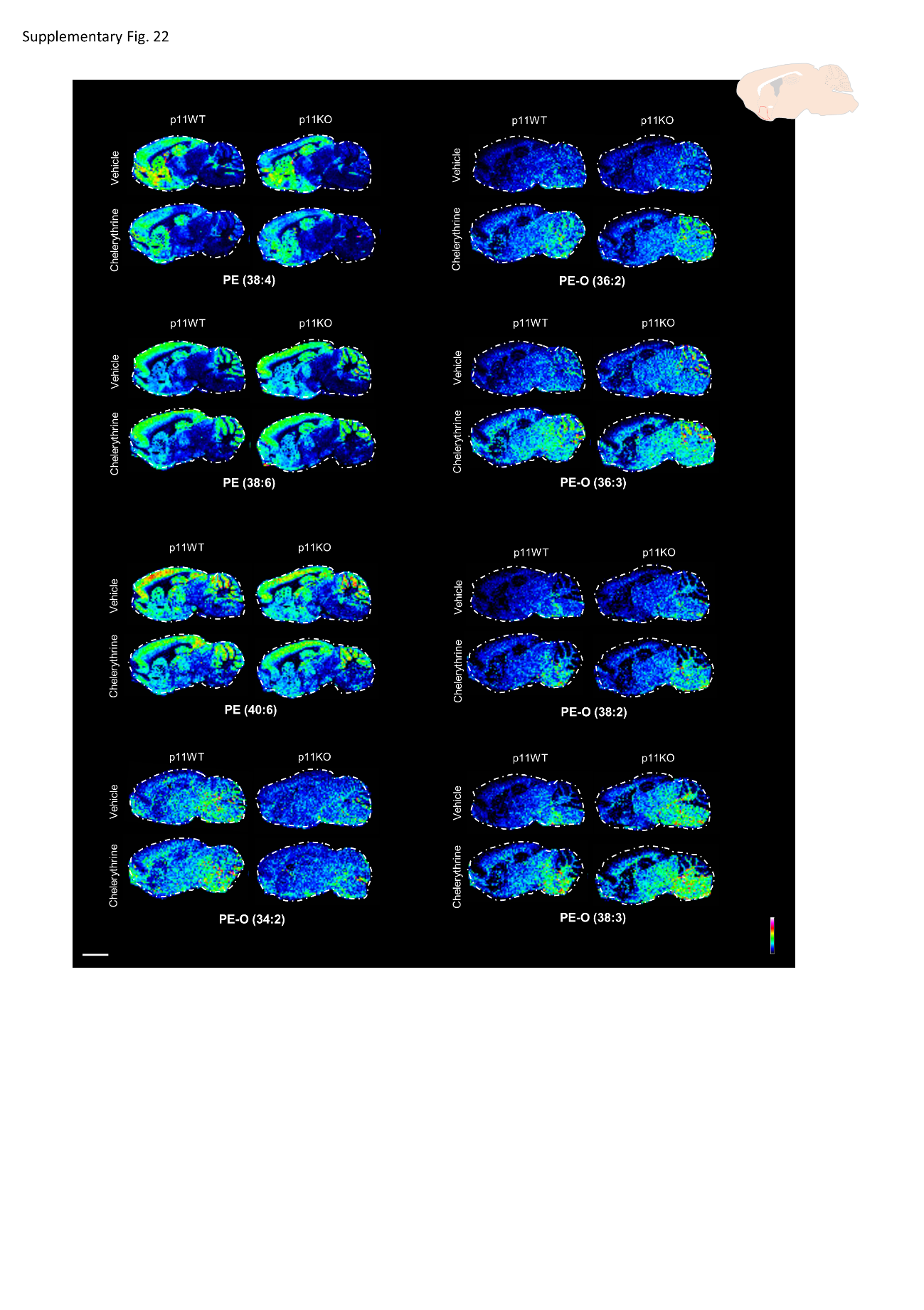
**

**Supplementary Fig. 25.** Effects of chelerythrine on phosphatidylethanolamine (PE) species levels in the nucleus accumbens. Representative ion images of PE(38:4), PE(38:6), PE(40:6), PE-O(34:2), PE-O(36:2), PE-O(36:3), PE-O(38:2), and PE-O(38:3) in the nucleus accumbens of p11WT or p11KO mice. MALDI-MSI ion images were presented as RMS normalized and acquired at a lateral resolution of 150 µm. Data are shown using a rainbow scale (representing ion intensity scale) for best visualization.

**
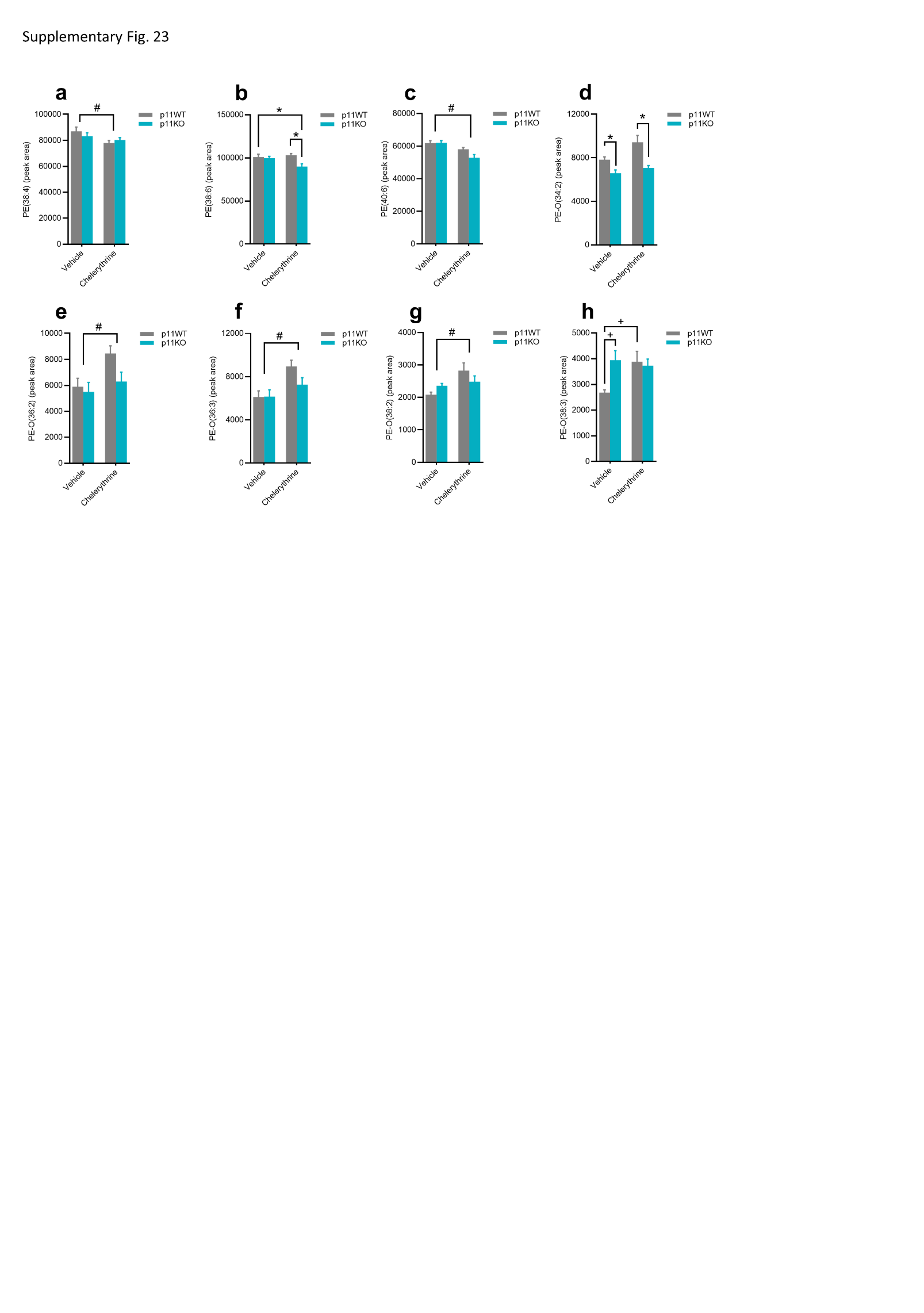
**

**Supplementary Fig. 26.** Altered levels of phosphatidylethanolamine (PE) species in the nucleus accumbens elicited by chelerythrine. Bar graph showing the quantification of the peaks of (**a**) PE(38:4), (**b**) PE(38:6), (**c**) PE(40:6), (**d**) PE-O(34:2), (**e**) PE-O(36:2), (**f**) PE-O(36:3), (**g**) PE-O(38:2), and (**h**) PE-O(38:3) in the nucleus accumbens of p11WT or p11KO mice. Values are expressed as means ± S.E.M. (*n* = 8). **p* < 0.05 compared with the p11WT group (i.e., a significant main effect of genotype, two-way ANOVA). #*p* < 0.05 compared with the vehicle-treated groups (i.e., a significant main effect of chelerythrine, two-way ANOVA), +*p* < 0.05 compared with the p11WT group (two-way ANOVA followed by Tukey’s post hoc test).

**
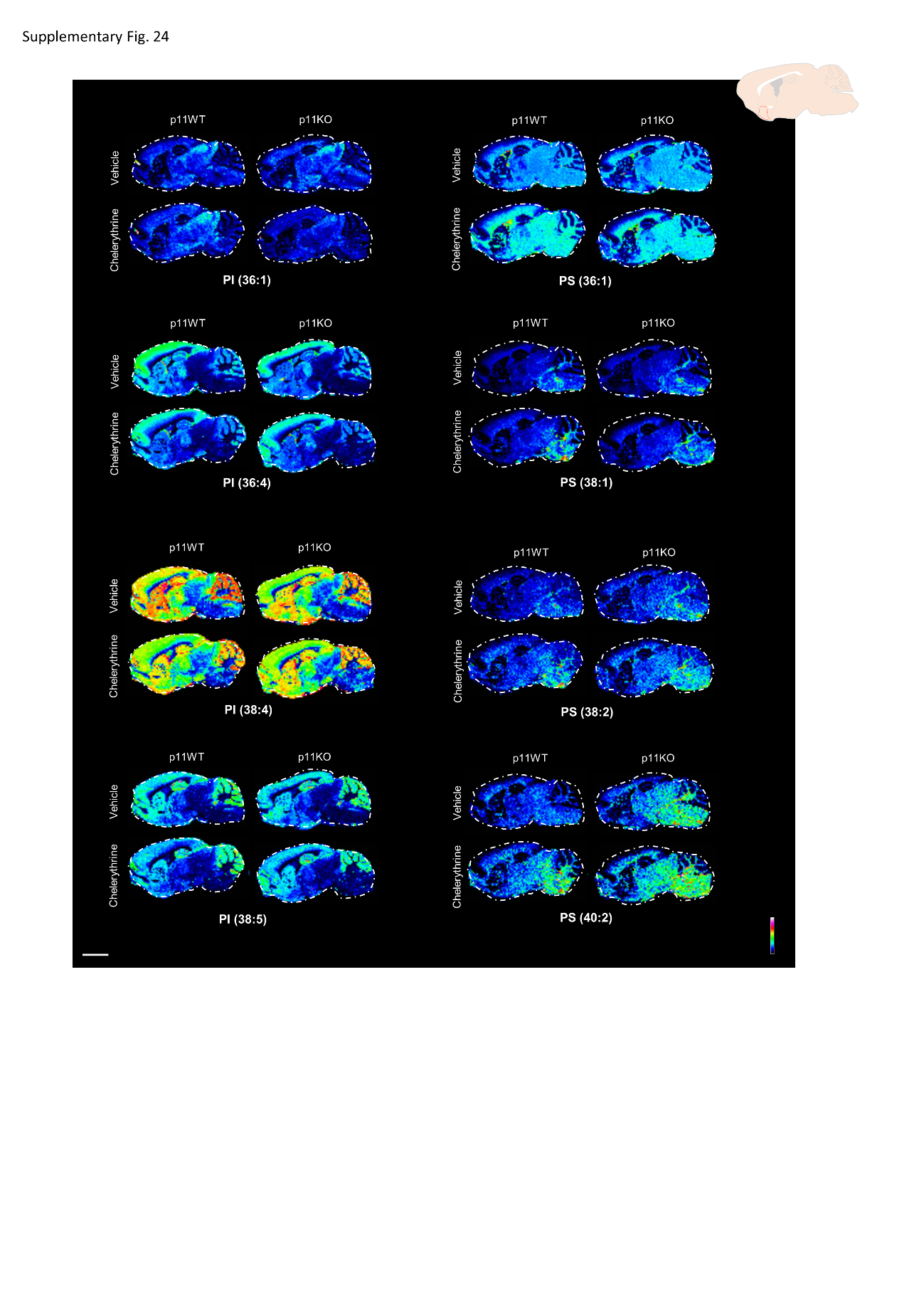
**

**Supplementary Fig. 27.** Effects of chelerythrine on phosphatidylinositol (PI) and phosphatidylserine (PS) species levels in the nucleus accumbens. Representative ion images of PI(36:1), PI(36:2), PI(36:4), PI(38:4), PI(38:5), PS(36:1), PS(38:1), PS(38:2), and PS(40:2) in the nucleus accumbens of p11WT or p11KO mice. MALDI-MSI ion images were presented as RMS normalized and acquired at a lateral resolution of 150 µm. Data are shown using a rainbow scale (representing ion intensity scale) for best visualization.

**
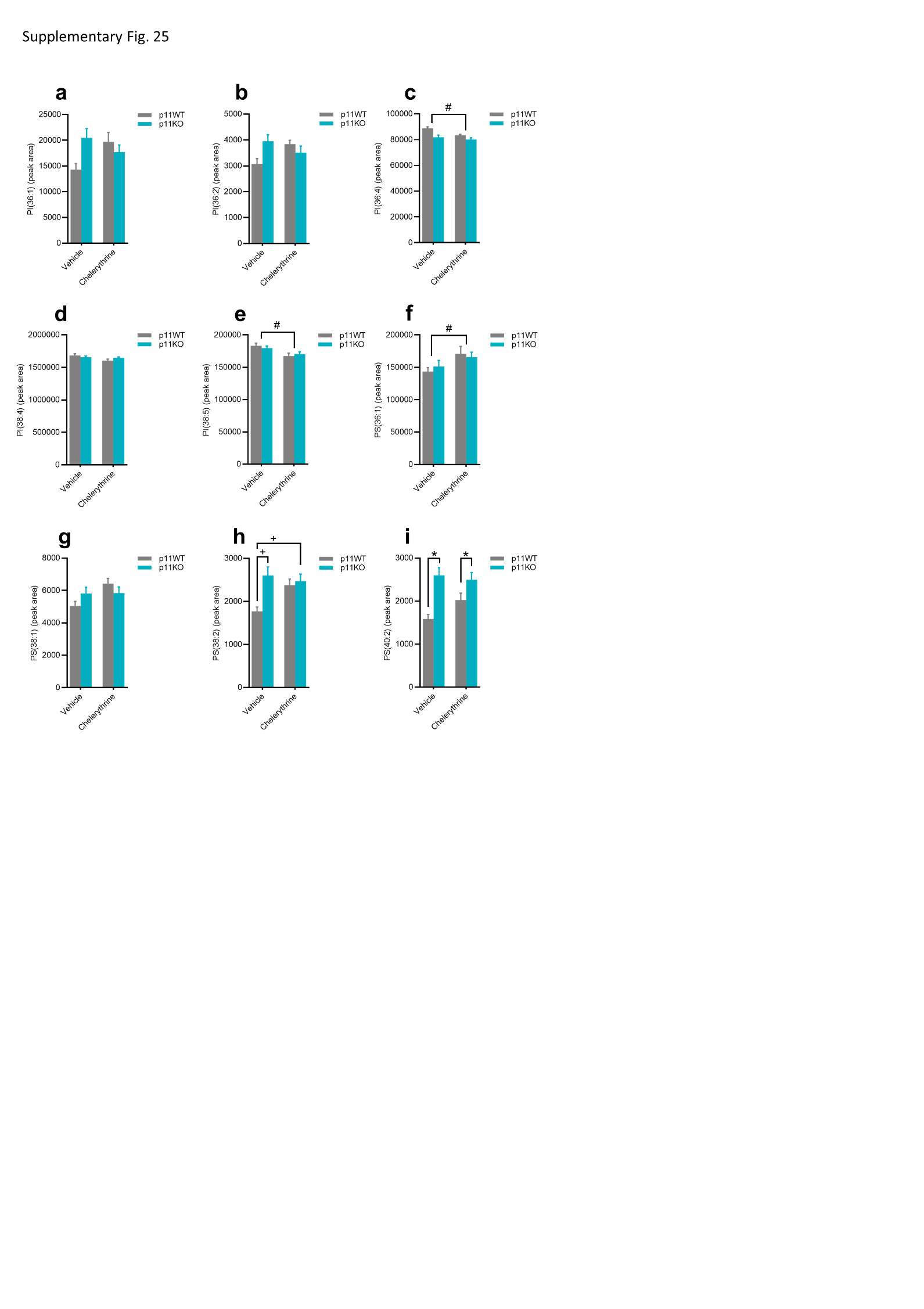
**

**Supplementary Fig. 28.** Altered levels of phosphatidylethanolamine (PE) species in the nucleus accumbens elicited by chelerythrine. Bar graph showing the quantification of the peaks of (**a**) PI(36:1), (**b**) PI(36:2), (**c**) PI(36:4), (**d**) PI(38:4), (**e**) PI(38:5), (**f**) PS(36:1), (**g**) PS(38:1), (**h**) PS(38:2), and (**i**) PS(40:2) in the nucleus accumbens of p11WT or p11KO mice. Values are expressed as means ± S.E.M. (*n* = 8). **p* < 0.05 compared with the p11WT group (i.e., a significant main effect of genotype, two-way ANOVA). #*p* < 0.05 compared with the vehicle-treated groups (i.e., a significant main effect of chelerythrine, two-way ANOVA), +*p* < 0.05 compared with the p11WT group (two-way ANOVA followed by Tukey’s post hoc test).

**
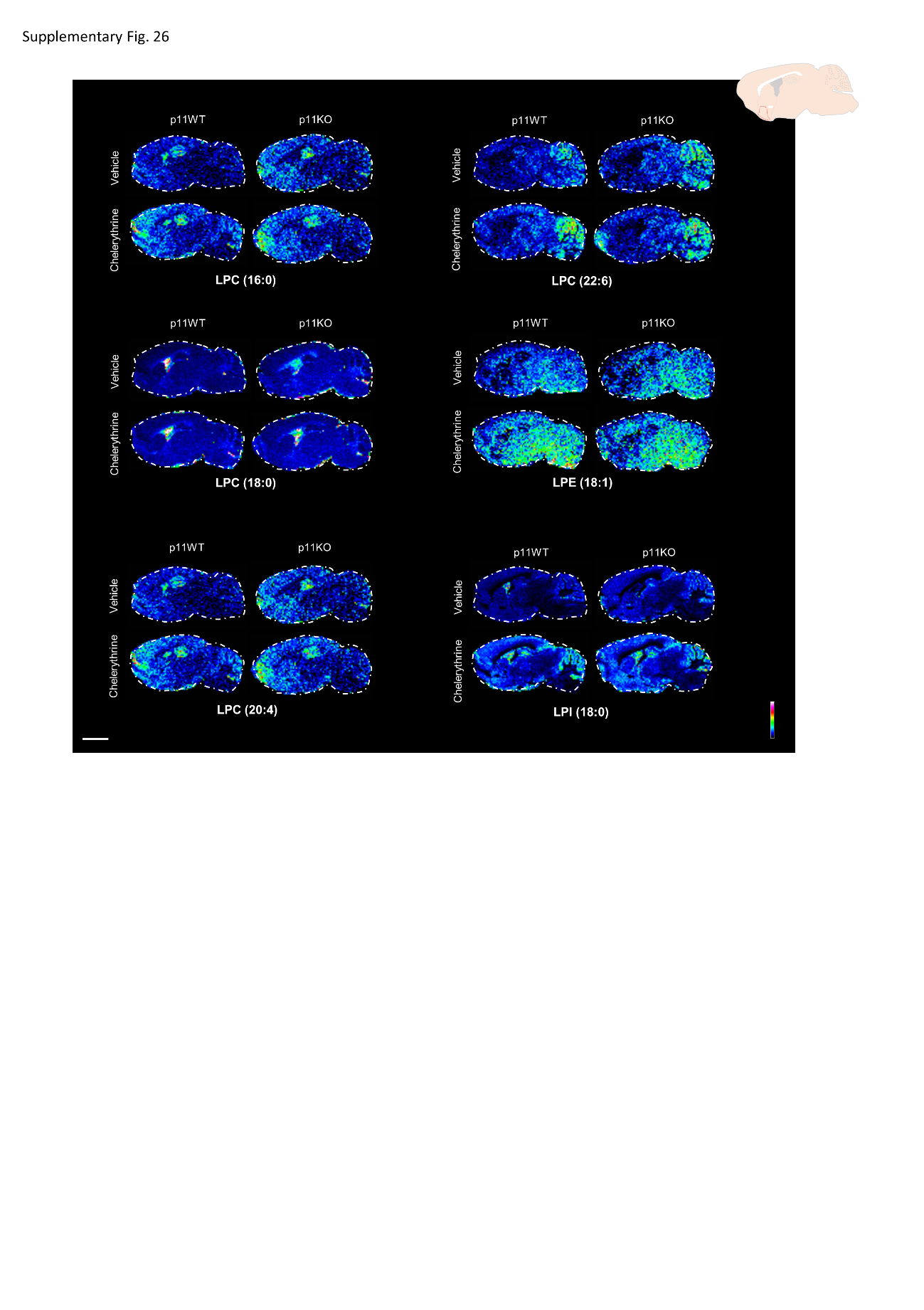
**

**Supplementary Fig. 29.** Effects of chelerythrine on lysophospholipid species levels, including lysophosphatidylcholine (LPC), lysophosphatidylethanolamine (LPE), and lysophosphatidylinositol (LPI), in the nucleus accumbens. Representative ion images of LPC(16:0), LPC(16:1), LPC(18:0), LPC(18:1), LPC(20:4), LPC(22:6), LPE(18:1), and LPI(18:0) in the nucleus accumbens of p11WT or p11KO mice. MALDI-MSI ion images were presented as RMS normalized and acquired at a lateral resolution of 150 µm. Data are shown using a rainbow scale (representing ion intensity scale) for best visualization.

**
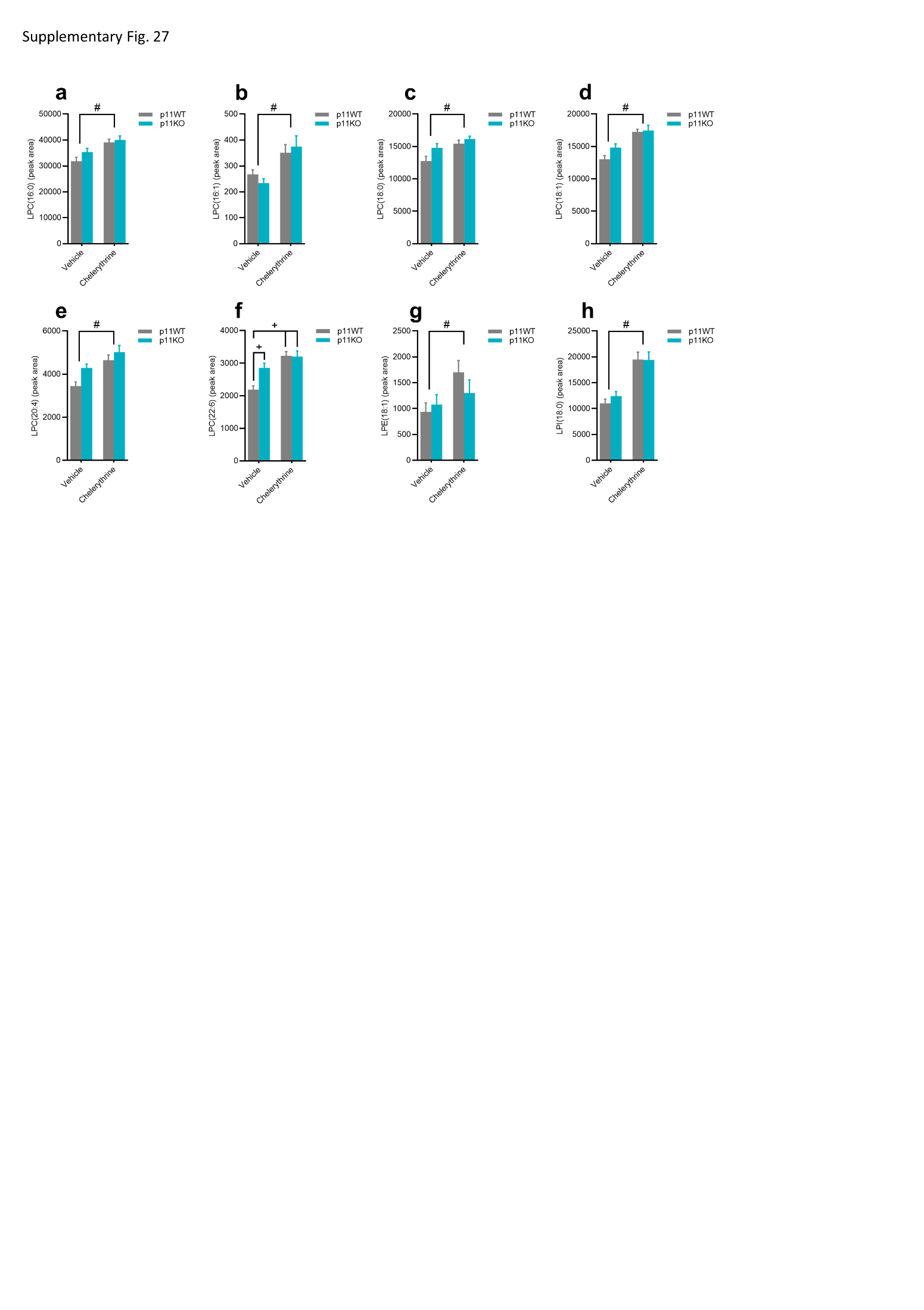
**

**Supplementary Fig. 30.** Chelerythrine induces an imbalance in the levels of lysophospholipid species in the nucleus accumbens elicited by chelerythrine. Bar graph showing the quantification of the peaks of (**a**) LPC(16:0), (**b**) LPC(16:1), (**c**) LPC(18:0), (**d**) LPC(18:1), (**e**) LPC(20:4), (**f**) LPC(22:6), (**g**) LPE(18:1), and (**h**) LPI(18:0) in the nucleus accumbens of p11WT or p11KO mice. Values are expressed as means ± S.E.M. (*n* = 8). #*p* < 0.05 compared with the vehicle-treated groups (i.e., a significant main effect of chelerythrine, two-way ANOVA), +*p* < 0.05 compared with the p11WT group (two-way ANOVA followed by Tukey’s post hoc test).


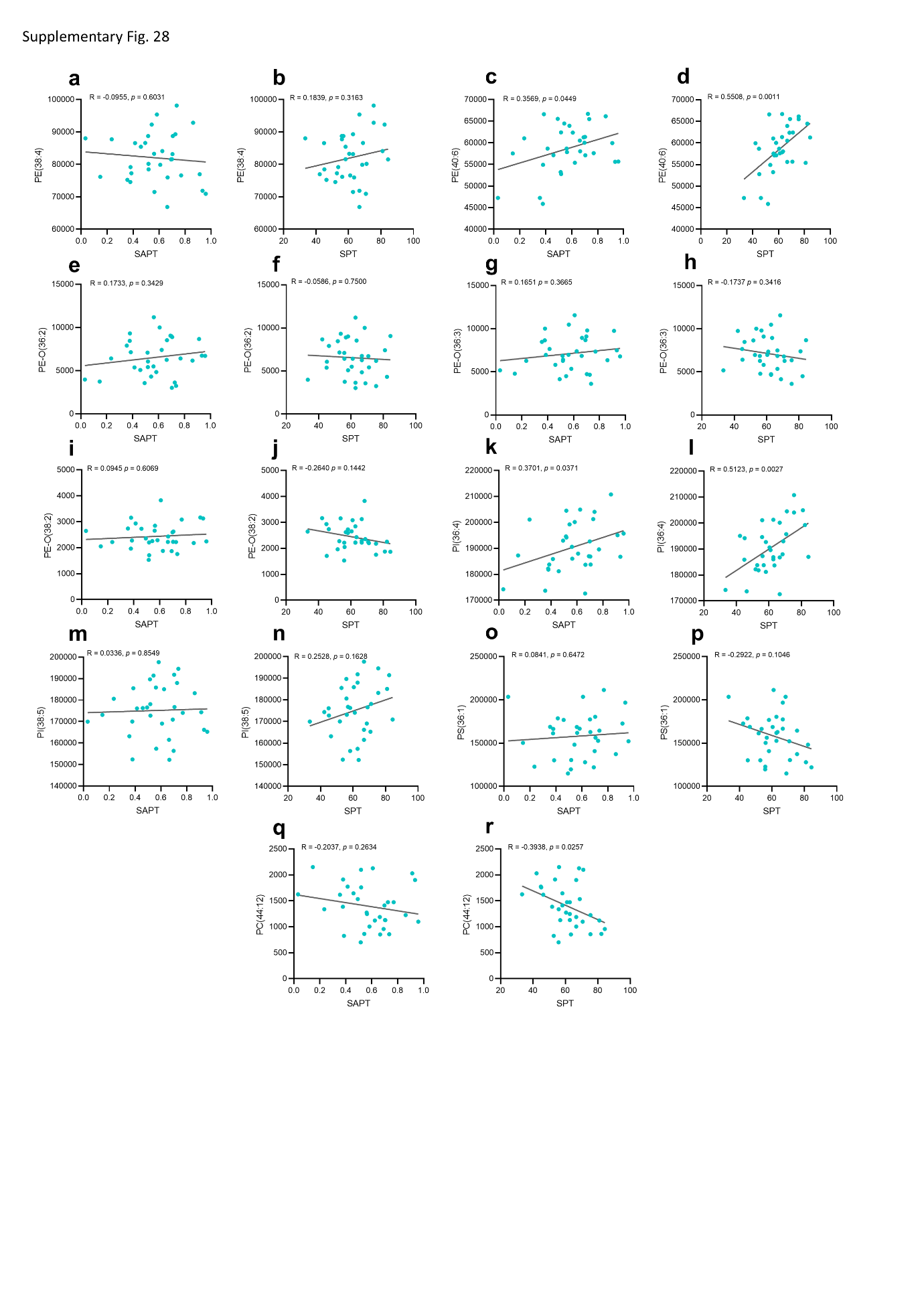


**Supplementary Fig. 31.** Altered levels of phospholipid species in the nucleus accumbens correlate with the depression-like phenotype induced by chelerythrine administration. Pearson´s correlation coefficient between PE(38:4), PE(40:6), PE-O(36:2), PE-O(36:3), PE-O(38:2), PI(36:4), PI(38:5), PS(36:1), and PC(44:12) levels and social affective preference test (**a**, **c**, **e**, **g**, **i**, **k**, **m**, **o**, and **q**) and sucrose preference test (**b**, **d**, **f**, **h**, **j**, **l**, **n**, **p**, and **r**).


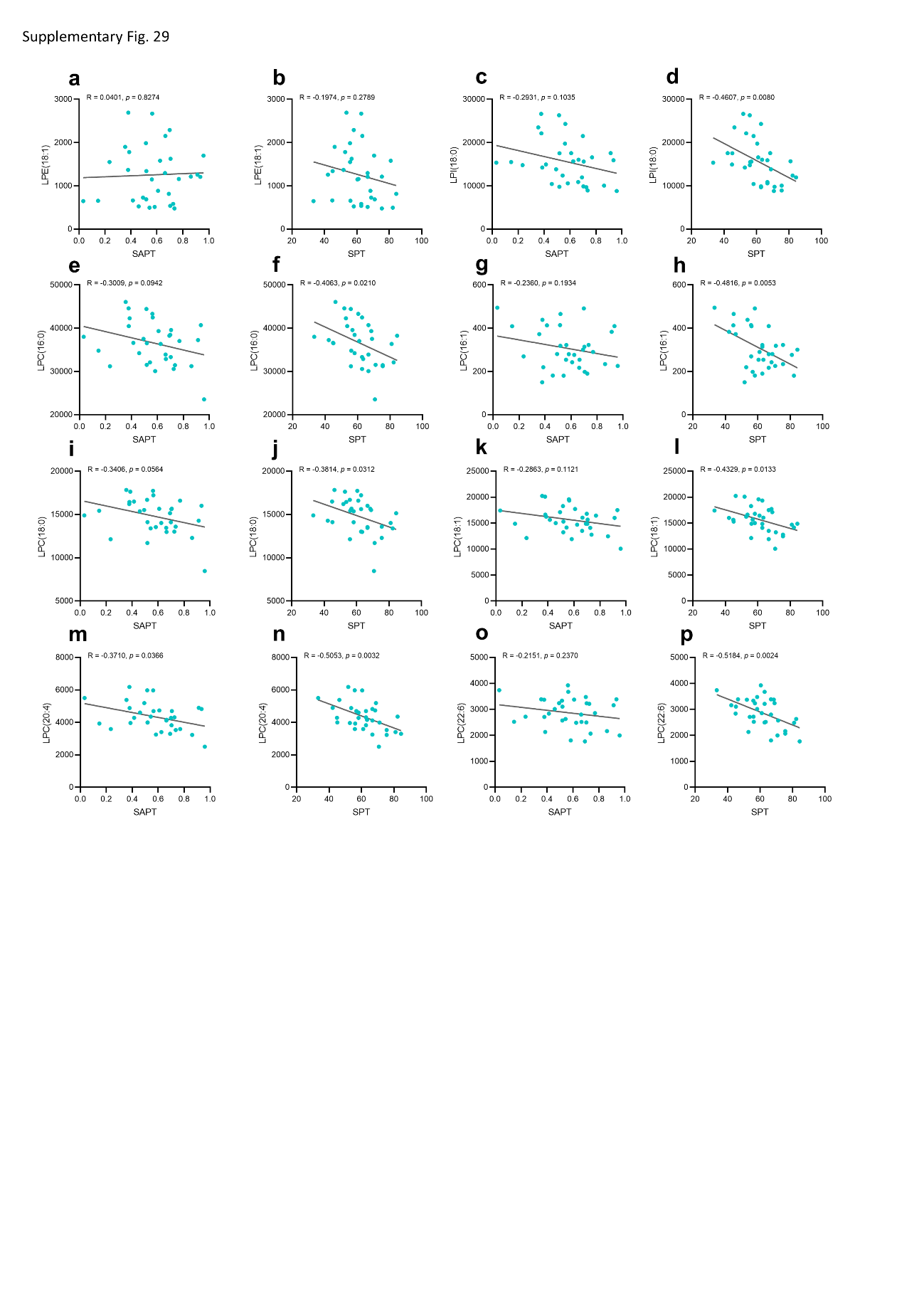


**Supplementary Fig. 32.** Altered levels of lysophospholipids species in the nucleus accumbens correlate with the depression-like phenotype induced by chelerythrine administration. Pearson´s correlation coefficient between LPE(18:1), LPI(18:0), LPC(16:0), LPC(16:1), LPC(18:0), LPC(18:1), LPC(20:4), and LPC(22:6) levels and social affective preference test (**a**, **c**, **e**, **g**, **i**, **k**, **m**, and **o**) and sucrose preference test (**b**, **d**, **f**, **h**, **j**, **l**, **n**, and **p**).


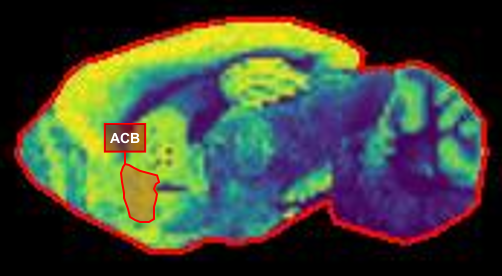


**Supplementary Fig. 33.** Representative annotation of nucleus accumbens.

**Supplementary Table 1.** List of assigned lipid species with high mass accuracy obtained from MALDI-FTICR-MSI experiments.

| **Lipid species assignment** | **Ion type** | **Formula** | **m/z theoretical** | **m/z observed** | **ppm error** |
| --- | --- | --- | --- | --- | --- |
| PE(34:1)** | [M-H]^-^ | C39H76NO8P | 716,523579 | 716,5248 | 1,7 |
| PE(36:2)** | [M-H]^-^ | C41H78NO8P | 742,539229 | 742,54009 | 1,2 |
| PE(36:4)** | [M-H]^-^ | C41H74NO8P | 738,507929 | 738,50865 | 1,0 |
| PE(38:6)** | [M-H]^-^ | C43H74NO8P | 762,507929 | 762,50828 | 0,5 |
| PE(38:5)** | [M-H]^-^ | C43H76NO8P | 764,523579 | 764,52513 | 2,0 |
| PE(38:4)** | [M-H]^-^ | C43H78NO8P | 766,539229 | 766,54007 | 1,1 |
| PE(38:4)** | [M+Nor-H]^-^ | C54H86N3O8P | 934,607977 | 934,60961 | 1,7 |
| PE(40:4)** | [M-H]^-^ | C45H82NO8P | 794,570529 | 794,57231 | 2,2 |
| PE(40:6)** | [M-H]^-^ | C45H78NO8P | 790,539229 | 790,53956 | 0,4 |
| PE(40:6)** | [M+Nor-H]^-^ | C56H86N3O8P | 958,607977 | 958,6112 | 3,4 |
| PE(40:7)* | [M-H]^-^ | C45H76NO8P | 788,523579 | 788,52515 | 2,0 |
| PE-O(34:2)** | [M-H]^-^ | C39H76NO7P | 700,528664 | 700,53 | 1,9 |
| PE-O(36:5)** | [M-H]^-^ | C41H74NO7P | 722,513014 | 722,5145 | 2,1 |
| PE-O(36:3)** | [M-H]^-^ | C41H78NO7P | 726,544314 | 726,54604 | 2,4 |
| PE-O(36:2)** | [M-H]^-^ | C41H80NO7P | 728,559964 | 728,56143 | 2,0 |
| PE-O(38:7)** | [M-H]^-^ | C43H74NO7P | 746,513014 | 746,51405 | 1,4 |
| PE-O(38:5)** | [M-H]^-^ | C43H78NO7P | 750,544314 | 750,54461 | 0,4 |
| PE-O(38:3)** | [M-H]^-^ | C43H82NO7P | 754,575615 | 754,57663 | 1,3 |
| PE-O(38:2)** | [M-H]^-^ | C43H84NO7P | 756,591265 | 756,593211 | 2,6 |
| PE-O(40:8)** | [M-H]^-^ | C45H76NO7P | 772,528664 | 772,52973 | 1,4 |
| PE-O(40:7)** | [M-H]^-^ | C45H78NO7P | 774,544314 | 774,54517 | 1,1 |
| PE-O(40:6)** | [M-H]^-^ | C45H80NO7P | 776,559964 | 776,56065 | 0,9 |
| PE-O(40:5)** | [M-H]^-^ | C45H82NO7P | 778,575615 | 778,57612 | 0,6 |
| PS(38:1)** | [M-H]^-^ | C44H84NO10P | 816,576008 | 816,57751 | 1,8 |
| PS(38:2)** | [M-H]^-^ | C44H82NO10P | 814,560358 | 814,56101 | 0,8 |
| PS(36:1)** | [M-H]^-^ | C42H80NO10P | 788,544708 | 788,5451 | 0,5 |
| PS(40:2)* | [M-H]^-^ | C46H86NO10P | 842,591659 | 842,59133 | -0,4 |
| PI(36:4)** | [M-H]^-^ | C45H79O13P | 857,518553 | 857,51912 | 0,7 |
| PI(36:2)* | [M-H]^-^ | C45H83O13P | 861,549853 | 861,55088 | 1,2 |
| PI(36:1)* | [M-H]^-^ | C45H85O13P | 863,565503 | 863,56683 | 1,5 |
| PI(38:4)** | [M-H]^-^ | C47H83O13P | 885,549853 | 885,55034 | 0,5 |
| PI(38:5)** | [M-H]^-^ | C47H81O13P | 883,534203 | 883,53299 | -1,4 |
| LPE 18:1* | [M-H]^-^ | C23H46NO7P | 478,293913 | 478,295511 | 3,3 |
| LPI 18:0** | [M-H]^-^ | C27H53O12P | 599,320188 | 599,3224 | 3,7 |
| PC 30:0** | [M+H]^+^ | C38H76NO8P | 706,538132 | 706,53929 | 1,6 |
| PC 30:0** | [M+K]^+^ | C38H76NO8PK | 744,494013 | 744,49551 | 2,0 |
| PC 32:0** | [M+K]^+^ | C40H80NO8PK | 772,525313 | 772,52341 | -2,5 |
| PC 32:0** | [M+Na]^+^ | C40H80NO8PNa | 756,551376 | 756,55319 | 2,4 |
| PC 32:0*' | [M+H]^+^ | C40H80NO8P | 734,569432 | 734,57037 | 1,3 |
| PC 32:0** | [M+Nor+H]^+^ | C51H88N3O8P | 902,63818 | 902,63857 | 0,4 |
| PC 32:1** | [M+K]^+^ | C40H78NO8PK | 770,509663 | 770,510661 | 1,3 |
| PC 32:1** | [M+Na]^+^ | C40H78NO8PNa | 754,535726 | 754,537281 | 2,1 |
| PC 32:1** | [M+H]^+^ | C40H78NO8P | 732,553782 | 732,55526 | 2,0 |
| PC 32:1** | [M+Nor+H]^+^ | C51H86N3O8P | 900,62253 | 900,62326 | 0,8 |
| PC(38:1)** | [M+H]^+^ | C46H90NO8P | 816,647682 | 816,64872 | 1,3 |
| PC(38:1)** | [M+K]^+^ | C46H90NO8PK | 854,603564 | 854,60316 | -0,5 |
| PC 40:7** | [M+H]^+^ | C48H82NO8P | 832,585082 | 832,58414 | -1,1 |
| PC 40:7** | [M+Na]^+^ | C48H82NO8PNa | 854,567026 | 854,56728 | 0,3 |
| PC 40:7** | [M+K]^+^ | C48H82NO8PK | 870,540964 | 870,540764 | -0,2 |
| PC 40:2** | [M+H]^+^ | C48H92NO8P | 842,663332 | 842,66312 | -0,3 |
| PC 40:2** | [M+K]^+^ | C48H92NO8PK | 880,619214 | 880,61976 | 0,6 |
| PC 42:1** | [M+H]^+^ | C50H98NO8P | 872,710283 | 872,71112 | 1,0 |
| PC 42:7** | [M+H]^+^ | C50H86NO8P | 860,616382 | 860,61622 | -0,2 |
| PC 42:7** | [M+K]^+^ | C50H86NO8PK | 898,572264 | 898,57271 | 0,5 |
| PC 42:7** | [M+Na]^+^ | C50H86NO8PNa | 882,598326 | 882,60008 | 2,0 |
| PC(44:12)** | [M+H]^+^ | C52H80NO8P | 878,569432 | 878,56961 | 0,2 |
| PC(44:12)** | [M+K]^+^ | C52H80NO8PK | 916,525313 | 916,52541 | 0,1 |
| LPC(16:0)** | [M+H]^+^ | C24H50NO7P | 496,339766 | 496,34093 | 2,3 |
| LPC 16:1* | [M+H]^+^ | C24H48NO7P | 494,324116 | 494,32516 | 2,1 |
| LPC(18:0)** | [M+H]^+^ | C26H54NO7P | 524,371067 | 524,37233 | 2,4 |
| LPC(18:1)* | [M+H]^+^ | C26H52NO7P | 522,355416 | 522,35685 | 2,7 |
| LPC(20:4)* | [M+H]^+^ | C28H50NO7P | 544,339766 | 544,34148 | 3,1 |
| LPC(22:6)* | [M+H]^+^ | C30H50NO7P | 568,339766 | 568,34089 | 2,0 |

A search of the m/z values with a 0.01 m/z mass tolerance in LIPID MAPS was conducted for both negative and positive polarities including all the ion types. Lipid species marked with two asterisks (**) were identified using mass accuracy, MS/MS and/or by comparing the observed distributions of different adducts of the same molecule across the sagittal mouse brain tissue sections. Lipids that are marked with one asterisk (*) were identified based solely on mass accuracy since the peak intensity was too low for MS/MS analysis of the tissue.

**Supplementary Table 1.** Description of statistical data of the Student’s t-test

| ***Parameter*** | ***t (df)*** | ***P value*** | ***Figure*** |  |
| --- | --- | --- | --- | --- |
| *PC(30:0) – p11KO-p11WT* | t _(8)_ = 2.81 | P=0.0228 | Suppl. Fig. 2, 3, and 4 |  |
| *PC(30:0) – p11WT.S-p11WT* | t _(8)_ = 0.67 | P=0.5212 | Suppl. Fig. 2, 3, and 4 |  |
| *PC(30:0) – p11KO.S-p11WT* | t _(8)_ = 3.35 | P=0.0101 | Suppl. Fig. 2, 3, and 4 |  |
| *PC(30:0) – p11KO.S-p11KO* | t _(8)_ = 0.98 | P=0.3536 | Suppl. Fig. 2, 3, and 4 |  |
| *PC(32:1) – p11KO-p11WT* | t _(8)_ = 0.22 | P=0.8249 | Suppl. Fig. 2, 3, and 4 |  |
| *PC(32:1) – p11WT.S-p11WT* | t _(8)_ = 2.66 | P=0.0285 | Suppl. Fig. 2, 3, and 4 |  |
| *PC(32:1) – p11KO.S-p11WT* | t _(8)_ = 0.07 | P=0.9391 | Suppl. Fig. 2, 3, and 4 |  |
| *PC(32:1) – p11KO.S-p11KO* | t _(8)_ = 0.11 | P=0.9211 | Suppl. Fig. 2, 3, and 4 |  |
| *PI(38:5) – p11KO-p11WT* | t _(8)_ = 0.45 | P=0.6582 | Suppl. Fig. 2, 3, and 4 |  |
| *PI(38:5) – p11WT.S-p11WT* | t _(8)_ = 0.74 | P=0.4782 | Suppl. Fig. 2, 3, and 4 |  |
| *PI(38:5) – p11KO.S-p11WT* | t _(8)_ = 2.36 | P=0.0432 | Suppl. Fig. 2, 3, and 4 |  |
| *PI(38:5) – p11KO.S-p11KO* | t _(8)_ = 1.67 | P=0.1327 | Suppl. Fig. 2, 3, and 4 |  |
| *PE(34:1) – p11KO-p11WT* | t _(8)_ = 1.21 | P=0.2589 | Suppl. Fig. 2, 6, and 7 |  |
| *PE(34:1) – p11WT.S-p11WT* | t _(8)_ = 0.72 | P=0.4920 | Suppl. Fig. 2, 6, and 7 |  |
| *PE(34:1) – p11KO.S-p11WT* | t _(8)_ = 1.75 | P=0.1176 | Suppl. Fig. 2, 6, and 7 |  |
| *PE(34:1) – p11KO.S-p11KO* | t _(8)_ = 1.75 | P=0.1176 | Suppl. Fig. 2, 6, and 7 |  |
| *PE(36:2) – p11KO-p11WT* | t _(8)_ = 1.18 | P=0.2708 | Suppl. Fig. 2, 6, and 7 |  |
| *PE(36:2) – p11WT.S-p11WT* | t _(8)_ = 0.31 | P=0.7704 | Suppl. Fig. 2, 6, and 7 |  |
| *PE(36:2) – p11KO.S-p11WT* | t _(8)_ = 1.73 | P=0.1205 | Suppl. Fig. 2, 6, and 7 |  |
| *PE(36:2) – p11KO.S-p11KO* | t _(8)_ = 1.04 | P=0.3265 | Suppl. Fig. 2, 6, and 7 |  |
| *PE(36:4) – p11KO-p11WT* | t _(8)_ = 0.25 | P=0.8036 | Suppl. Fig. 2, 6, and 7 |  |
| *PE(36:4) – p11WT.S-p11WT* | t _(8)_ = 0.97 | P=0.3590 | Suppl. Fig. 2, 6, and 7 |  |
| *PE(36:4) – p11KO.S-p11WT* | t _(8)_ = 2.04 | P=0.0751 | Suppl. Fig. 2, 6, and 7 |  |
| *PE(36:4) – p11KO.S-p11KO* | t _(8)_ = 1.13 | P=0.2880 | Suppl. Fig. 2, 6, and 7 |  |
| *PE(38:4) – p11KO-p11WT* | t _(8)_ = 0.04 | P=0.9676 | Suppl. Fig. 2, 6, and 7 |  |
| *PE(38:4) – p11WT.S-p11WT* | t _(8)_ = 0.81 | P=0.4408 | Suppl. Fig. 2, 6, and 7 |  |
| *PE(38:4) – p11KO.S-p11WT* | t _(8)_ = 2.35 | P=0.0430 | Suppl. Fig. 2, 6, and 7 |  |
| *PE(38:4) – p11KO.S-p11KO* | t _(8)_ = 1.37 | P=0.2063 | Suppl. Fig. 2, 6, and 7 |  |
| *PE(38:5) – p11KO-p11WT* | t _(8)_ = 0.39 | P=0.7024 | Suppl. Fig. 2, 6, and 7 |  |
| *PE(38:5) – p11WT.S-p11WT* | t _(8)_ = 1.12 | P=0.2941 | Suppl. Fig. 2, 6, and 7 |  |
| *PE(38:5) – p11KO.S-p11WT* | t _(8)_ = 2.31 | P=0.0498 | Suppl. Fig. 2, 6, and 7 |  |
| *PE(38:5) – p11KO.S-p11KO* | t _(8)_ = 2.32 | P=0.0495 | Suppl. Fig. 2, 6, and 7 |  |
| *PE(38:6) – p11KO-p11WT* | t _(8)_ = 0.43 | P=0.6759 | Suppl. Fig. 2, 6, and 7 |  |
| *PE(38:6) – p11WT.S-p11WT* | t _(8)_ = 0.38 | P=0.7112 | Suppl. Fig. 2, 6, and 7 |  |
| *PE(38:6) – p11KO.S-p11WT* | t _(8)_ = 2.37 | P=0.0428 | Suppl. Fig. 2, 6, and 7 |  |
| *PE(38:6) – p11KO.S-p11KO* | t _(8)_ = 1.35 | P=0.2118 | Suppl. Fig. 2, 6, and 7 |  |
| *PE(40:4) – p11KO-p11WT* | t _(8)_ = 0.09 | P=0.9283 | Suppl. Fig. 2, 6, and 7 |  |
| *PE(40:4) – p11WT.S-p11WT* | t _(8)_ = 1.57 | P=0.1536 | Suppl. Fig. 2, 6, and 7 |  |
| *PE(40:4) – p11KO.S-p11WT* | t _(8)_ = 1.89 | P=0.0942 | Suppl. Fig. 2, 6, and 7 |  |
| *PE(40:4) – p11KO.S-p11KO* | t _(8)_ = 1.81 | P=0.1090 | Suppl. Fig. 2, 6, and 7 |  |
| *PE(40:6) – p11KO-p11WT* | t _(8)_ = 0.07 | P=0.9448 | Suppl. Fig. 2, 6, and 7 |  |
| *PE(40:6) – p11WT.S-p11WT* | t _(8)_ = 0.43 | P=0.6729 | Suppl. Fig. 2, 6, and 7 |  |
| *PE(40:6) – p11KO.S-p11WT* | t _(8)_ = 2.32 | P=0.0491 | Suppl. Fig. 2, 6, and 7 |  |
| *PE(40:6) – p11KO.S-p11KO* | t _(8)_ = 1.44 | P=0.1869 | Suppl. Fig. 2, 6, and 7 |  |
| *PE(40:7) – p11KO-p11WT* | t _(8)_ = 0.17 | P=0.8686 | Suppl. Fig. 2, 6, and 7 |  |
| *PE(40:7) – p11WT.S-p11WT* | t _(8)_ = 0.69 | P=0.5089 | Suppl. Fig. 2, 6, and 7 |  |
| *PE(40:7) – p11KO.S-p11WT* | t _(8)_ = 2.31 | P=0.0493 | Suppl. Fig. 2, 6, and 7 |  |
| *PE(40:7) – p11KO.S-p11KO* | t _(8)_ = 1.36 | P=0.2099 | Suppl. Fig. 2, 6, and 7 |  |
| *PE-O(34:2) – p11KO-p11WT* | | t _(8)_ = 0.91 | P=0.3931 | Suppl. Fig. 2, 9, and 10 |
| *PE-O(34:2) – p11WT.S-p11WT* | t _(8)_ = 0.42 | P=0.6789 | Suppl. Fig. 2, 9, and 10 |  |
| *PE-O(34:2) – p11KO.S-p11WT* | t _(8)_ = 2.05 | P=0.0738 | Suppl. Fig. 2, 9, and 10 |  |
| *PE-O(34:2) – p11KO.S-p11KO* | t _(8)_ = 1.28 | P=0.2339 | Suppl. Fig. 2, 9, and 10 |  |
| *PE-O(36:2) – p11KO-p11WT* | t _(8)_ = 1.35 | P=0.2132 | Suppl. Fig. 2, 9, and 10 |  |
| *PE-O(36:2) – p11WT.S-p11WT* | t _(8)_ = 0.95 | P=0.3681 | Suppl. Fig. 2, 9, and 10 |  |
| *PE-O(36:2) – p11KO.S-p11WT* | t _(8)_ = 2.42 | P=0.0415 | Suppl. Fig. 2, 9, and 10 |  |
| *PE-O(36:2) – p11KO.S-p11KO* | t _(8)_ = 1.35 | P=0.2112 | Suppl. Fig. 2, 9, and 10 |  |
| *PE-O(36:3) – p11KO-p11WT* | t _(8)_ = 1.19 | P=0.2678 | Suppl. Fig. 1, 8, and 9 |  |
| *PE-O(36:3) – p11WT.S-p11WT* | t _(8)_ = 0.42 | P=0.6803 | Suppl. Fig. 2, 9, and 10 |  |
| *PE-O(36:3) – p11KO.S-p11WT* | t _(8)_ = 2.29 | P=0.0510 | Suppl. Fig. 2, 9, and 10 |  |
| *PE-O(36:3) – p11KO.S-p11KO* | t _(8)_ = 2.10 | P=0.0679 | Suppl. Fig. 2, 9, and 10 |  |
| *PE-O(38:5) – p11KO-p11WT* | t _(8)_ = 0.27 | P=0.7918 | Suppl. Fig. 2, 9, and 10 |  |
| *PE-O(38:5) – p11WT.S-p11WT* | t _(8)_ = 0.70 | P=0.5021 | Suppl. Fig. 2, 9, and 10 |  |
| *PE-O(38:5) – p11KO.S-p11WT* | t _(8)_ = 1.16 | P=0.2792 | Suppl. Fig. 2, 9, and 10 |  |
| *PE-O(38:5) – p11KO.S-p11KO* | t _(8)_ = 1.01 | P=0.3420 | Suppl. Fig. 2, 9, and 10 |  |
| *PE-O(38:7) – p11KO-p11WT* | t _(8)_ = 0.38 | P=0.7127 | Suppl. Fig. 2, 9, and 10 |  |
| *PE-O(38:7) – p11WT.S-p11WT* | t _(8)_ = 0.38 | P=0.7127 | Suppl. Fig. 2, 9, and 10 |  |
| *PE-O(38:7) – p11KO.S-p11WT* | t _(8)_ = 0.50 | P=0.6278 | Suppl. Fig. 2, 9, and 10 |  |
| *PE-O(38:7) – p11KO.S-p11KO* | t _(8)_ = 0.83 | P=0.4282 | Suppl. Fig. 2, 9, and 10 |  |
| *PE-O(40:5) – p11KO-p11WT* | t _(8)_ = 0.38 | P=0.7068 | Suppl. Fig. 2, 9, and 10 |  |
| *PE-O(40:5) – p11WT.S-p11WT* | t _(8)_ = 1.07 | P=0.3135 | Suppl. Fig. 2, 9, and 10 |  |
| *PE-O(40:5) – p11KO.S-p11WT* | t _(8)_ = 1.63 | P=0.1401 | Suppl. Fig. 2, 9, and 10 |  |
| *PE-O(40:5) – p11KO.S-p11KO* | t _(8)_ = 1.88 | P=0.0961 | Suppl. Fig. 2, 9, and 10 |  |
| *PE-O(40:6) – p11KO-p11WT* | t _(8)_ = 0.15 | P=0.8782 | Suppl. Fig. 2, 9, and 10 |  |
| *PE-O(40:6) – p11WT.S-p11WT* | t _(8)_ = 0.73 | P=0.4838 | Suppl. Fig. 2, 9, and 10 |  |
| *PE-O(40:6) – p11KO.S-p11WT* | t _(8)_ = 2.91 | P=0.0194 | Suppl. Fig. 2, 9, and 10 |  |
| *PE-O(40:6) – p11KO.S-p11KO* | t _(8)_ = 1.68 | P=0.1311 | Suppl. Fig. 2, 9, and 10 |  |
| *PE-O(40:7) – p11KO-p11WT* | t _(8)_ = 0.24 | P=0.8091 | Suppl. Fig. 2, 9, and 10 |  |
| *PE-O(40:7) – p11WT.S-p11WT* | t _(8)_ = 1.72 | P=0.1238 | Suppl. Fig. 2, 9, and 10 |  |
| *PE-O(40:7) – p11KO.S-p11WT* | t _(8)_ = 2.13 | P=0.0653 | Suppl. Fig. 2, 9, and 10 |  |
| *PE-O(40:7) – p11KO.S-p11KO* | t _(8)_ = 2.81 | P=0.0363 | Suppl. Fig. 2, 9, and 10 |  |
| *PE-O(40:8) – p11KO-p11WT* | t _(8)_ = 0.01 | P=0.9911 | Suppl. Fig. 2, 9, and 10 |  |
| *PE-O(40:8) – p11WT.S-p11WT* | t _(8)_ = 0.54 | P=0.6037 | Suppl. Fig. 2, 9, and 10 |  |
| *PE-O(40:8) – p11KO.S-p11WT* | t _(8)_ = 1.53 | P=0.1630 | Suppl. Fig. 2, 9, and 10 |  |
| *PE-O(40:8) – p11KO.S-p11KO* | t _(8)_ = 1.16 | P=0.2784 | Suppl. Fig. 2, 9, and 10 |  |

Student’s t test.

| **Supplementary Table 2.** Description of statistical data of the two-way ANOVA | | | | | | | | |
| --- | --- | --- | --- | --- | --- | --- | --- | --- |
|  | **Stress** | | **Genotype** | | **Stress x Genotype** | | |  |
| ***Parameter*** | ***F (df)*** | ***P value*** | ***F (df)*** | ***P value*** | ***F (df)*** | ***P value*** | ***Figure*** | |
| *SAPT: time interacting* | F _(1, 28)_ = 1.03 | P=0.31 | F _(1, 28)_ = 4.75 | P<0.05 | F _(1, 28)_ = 0.20 | P=0.65 | Fig. 1 | |
| *SAPT: social preference index* | F _(1, 28)_ = 0.05 | P=0.81 | F _(1, 28)_ = 21.5 | P<0.01 | F _(1, 28)_ = 0.35 | P=0.55 | Fig. 1 | |
| *SPT: sucrose preference* | F _(1, 28)_ = 1.38 | P=0.24 | F _(1, 28)_ = 50.7 | P<0.01 | F _(1, 28)_ = 0.61 | P=0.44 | Fig. 1 | |
| *SPT: total intake* | F _(1, 28)_ = 0.79 | P=0.37 | F _(1, 28)_ = 0.21 | P=0.64 | F _(1, 28)_ = 1.07 | P=0.30 | Fig. 1 | |
| *TST: Immobility time* | F _(1, 28)_ = 3.06 | P=0.09 | F _(1, 28)_ = 85.5 | P<0.01 | F _(1, 28)_ = 5.76 | P<0.01 | Fig. 1 | |
| *OFT: Distance traveled* | F _(1, 28)_ = 0.11 | P=0.73 | F _(1, 28)_ = 0.18 | P=0.66 | F _(1, 28)_ = 0.75 | P=0.39 | Fig. 1 | |
| *OFT: Velocity* | F _(1, 28)_ = 0.13 | P=0.71 | F _(1, 28)_ = 0.03 | P=0.85 | F _(1, 28)_ = 0.04 | P=0.83 | Fig. 1 | |
| *OFT: Time in the center* | F _(1, 28)_ = 0.07 | P=0.92 | F _(1, 28)_ = 0.72 | P=0.40 | F _(1, 28)_ = 0.29 | P=0.59 | Suppl. Fig. 1 | |
| *PC(30.1)* | F _(1, 16)_ = 1.33 | P=0.26 | F _(1, 16)_ = 14.1 | P<0.01 | F _(1, 16)_ = 0.02 | P=0.87 | Fig. 2, Suppl. Fig. 5 | |
| *PC(32.1)* | F _(1, 16)_ = 2.79 | P=0.11 | F _(1, 16)_ = 2.39 | P=0.14 | F _(1, 16)_ = 1.88 | P=0.18 | Fig. 2, Suppl. Fig. 5 | |
| *PI(38.5)* | F _(1, 16)_ = 3.78 | P=0.07 | F _(1, 28)_ = 0.49 | P=0.48 | F _(1, 28)_ = 0.09 | P=0.92 | Fig. 2, Suppl. Fig. 5 | |
| *PE(34.1)* | F _(1, 16)_ = 1.44 | P=0.24 | F _(1, 16)_ = 4.12 | P=0.05 | F _(1, 16)_ = 0.02 | P=0.86 | Fig. 2, Suppl. Fig. 8 | |
| *PE(36.2)* | F _(1, 16)_ = 0.76 | P=0.39 | F _(1, 16)_ = 5.38 | P<0.05 | F _(1, 16)_ = 0.15 | P=0.69 | Fig. 2, Suppl. Fig. 8 | |
| *PE(36.4)* | F _(1, 16)_ = 2.24 | P=0.15 | F _(1, 16)_ = 0.41 | P=0.53 | F _(1, 16)_ = 0.03 | P=0.84 | Fig. 2, Suppl. Fig. 8 | |
| *PE(38.4)* | F _(1, 16)_ = 2.43 | P=0.13 | F _(1, 16)_ = 0.27 | P=0.61 | F _(1, 16)_ = 0.20 | P=0.65 | Fig. 2, Suppl. Fig. 8 | |
| *PE(38.5)* | F _(1, 16)_ = 5.12 | P<0.05 | F _(1, 16)_ = 1.31 | P=0.26 | F _(1, 16)_ = 0.19 | P=0.66 | Fig. 2, Suppl. Fig. 8 | |
| *PE(38.6)* | F _(1, 16)_ = 1.28 | P=0.27 | F _(1, 16)_ = 1.36 | P=0.26 | F _(1, 16)_ = 0.27 | P=0.60 | Fig. 2, Suppl. Fig. 8 | |
| *PE(40.4)* | F _(1, 16)_ = 5.73 | P<0.05 | F _(1, 16)_ = 0.03 | P=0.86 | F _(1, 16)_ = 0.11 | P=0.73 | Fig. 2, Suppl. Fig. 8 | |
| *PE(40.6)* | F _(1, 16)_ = 1.61 | P=0.22 | F _(1, 16)_ = 0.51 | P=0.48 | F _(1, 16)_ = 0.36 | P=0.55 | Fig. 2, Suppl. Fig. 8 | |
| *PE(40.7)* | F _(1, 16)_ = 2.06 | P=0.17 | F _(1, 16)_ = 0.46 | P=0.51 | F _(1, 16)_ = 0.18 | P=0.67 | Fig. 2, Suppl. Fig. 8 | |
| *PE-O(34.2)* | F _(1, 16)_ = 0.97 | P=0.33 | F _(1, 16)_ = 5.43 | P<0.05 | F _(1, 16)_ = 0.12 | P=0.73 | Fig. 2, Suppl. Fig. 11 | |
| *PE-O(36.2)* | F _(1, 16)_ = 2.67 | P=0.12 | F _(1, 16)_ = 4.58 | P<0.05 | F _(1, 16)_ = 0.08 | P=0.77 | Fig. 2, Suppl. Fig. 11 | |
| *PE-O(36.3)* | F _(1, 16)_ = 2.11 | P=0.16 | F _(1, 16)_ = 5.04 | P<0.05 | F _(1, 16)_ = 0.67 | P=0.42 | Fig. 2, Suppl. Fig. 11 | |
| *PE-O(36.5)* | F _(1, 16)_ = 0.22 | P=0.64 | F _(1, 16)_ = 0.11 | P=0.74 | F _(1, 16)_ = 0.02 | P=0.92 | Fig. 2, Suppl. Fig. 11 | |
| *PE-O(38.5)* | F _(1, 16)_ = 1.49 | P=0.23 | F _(1, 16)_ = 0.01 | P=0.91 | F _(1, 16)_ = 0.12 | P=0.72 | Fig. 2, Suppl. Fig. 11 | |
| *PE-O(38.7)* | F _(1, 16)_ = 0.55 | P=0.46 | F _(1, 16)_ = 0.04 | P=0.82 | F _(1, 16)_ = 0.08 | P=0.77 | Fig. 2, Suppl. Fig. 11 | |
| *PE-O(40.5)* | F _(1, 16)_ = 4.53 | P<0.05 | F _(1, 16)_ = 0.02 | P=0.96 | F _(1, 16)_ = 0.51 | P=0.48 | Fig. 2, Suppl. Fig. 11 | |
| *PE-O(40.6)* | F _(1, 16)_ = 6.41 | P<0.05 | F _(1, 16)_ = 0.52 | P=0.48 | F _(1, 16)_ = 0.65 | P=0.43 | Fig. 2, Suppl. Fig. 11 | |
| *PE-O(40.7)* | F _(1, 16)_ = 6.10 | P<0.05 | F _(1, 16)_ = 0.01 | P=0.98 | F _(1, 16)_ = 0.20 | P=0.65 | Fig. 2, Suppl. Fig. 11 | |
| *PE-O(40.8)* | F _(1, 16)_ = 1.32 | P=0.26 | F _(1, 16)_ = 0.09 | P=0.76 | F _(1, 16)_ = 0.09 | P=0.75 | Fig. 2, Suppl. Fig. 11 | |
| *Etnk1 – AcbC* | F _(1, 16)_ = 3.33 | P=0.08 | F _(1, 16)_ = 0.41 | P=0.52 | F _(1, 16)_ = 0.03 | P=0.86 | Fig. 3 | |
| *Chkb – AcbC* | F _(1, 16)_ = 5.61 | P<0.05 | F _(1, 16)_ = 0.36 | P=0.55 | F _(1, 16)_ = 0.02 | P=0.87 | Fig. 3 | |
| *Pcyt2 – AcbC* | F _(1, 16)_ = 3.25 | P=0.08 | F _(1, 16)_ = 1.15 | P=0.29 | F _(1, 16)_ = 1.23 | P=0.28 | Fig. 3 | |
| *Cept1 – AcbC* | F _(1, 16)_ = 1.14 | P=0.22 | F _(1, 16)_ = 3.14 | P=0.09 | F _(1, 16)_ = 0.01 | P=0.96 | Fig. 3 | |
| *Gnpat – AcbC* | F _(1, 16)_ = 2.01 | P=0.17 | F _(1, 16)_ = 14.53 | P<0.01 | F _(1, 16)_ = 4.06 | P=0.06 | Fig. 3 | |
| *Pemt – AcbC* | F _(1, 16)_ = 0.01 | P=0.95 | F _(1, 16)_ = 0.03 | P=0.86 | F _(1, 16)_ = 3.65 | P=0.07 | Fig. 3 | |
| *Ptdss1 – AcbC* | F _(1, 16)_ = 10.6 | P<0.01 | F _(1, 16)_ = 0.08 | P=0.77 | F _(1, 16)_ = 0.40 | P=0.53 | Fig. 3 | |
| *Ptdss2 – AcbC* | F _(1, 16)_ = 0.16 | P=0.68 | F _(1, 16)_ = 0.17 | P=0.67 | F _(1, 16)_ = 0.02 | P=0.86 | Fig. 3 | |
| *Phospho1– AcbC* | F _(1, 16)_ = 6.21 | P<0.05 | F _(1, 16)_ = 2.02 | P=0.17 | F _(1, 16)_ = 0.49 | P=0.49 | Fig. 3 | |
| *Pisd – AcbC* | F _(1, 16)_ = 2.12 | P=0.16 | F _(1, 16)_ = 1.17 | P=0.29 | F _(1, 16)_ = 0.13 | P=0.71 | Fig. 3 | |
| *Lpcat4 – AcbC* | F _(1, 16)_ = 0.01 | P=0.92 | F _(1, 16)_ = 0.48 | P=0.49 | F _(1, 16)_ = 2.05 | P=0.17 | Fig. 3 | |
| *Stard7 – AcbC* | F _(1, 16)_ = 3.81 | P=0.06 | F _(1, 16)_ = 12.2 | P<0.01 | F _(1, 16)_ = 1.88 | P=0.18 | Fig. 3 | |
| *Etnk1 – AcbSh* | F _(1, 16)_ = 0.48 | P=0.49 | F _(1, 16)_ = 1.93 | P=0.18 | F _(1, 16)_ = 0.43 | P=0.52 | Fig. 3, Suppl. Fig. 19 | |
| *Chkb – AcbSh* | F _(1, 16)_ = 0.59 | P=0.45 | F _(1, 16)_ = 0.01 | P=0.95 | F _(1, 16)_ = 0.03 | P=0.85 | Fig. 3, Suppl. Fig. 19 | |
| *Pcyt2 – AcbSh* | F _(1, 16)_ = 1.01 | P=0.32 | F _(1, 16)_ = 0.22 | P=0.63 | F _(1, 16)_ = 0.08 | P=0.77 | Fig. 3, Suppl. Fig. 19 | |
| *Cept1 – AcbSh* | F _(1, 16)_ = 1.33 | P=0.26 | F _(1, 16)_ = 0.05 | P=0.82 | F _(1, 16)_ = 0.40 | P=0.53 | Fig. 3, Suppl. Fig. 19 | |
| *Gnpat – AcbSh* | F _(1, 16)_ = 0.02 | P=0.88 | F _(1, 16)_ = 0.19 | P=0.66 | F _(1, 16)_ = 0.01 | P=0.99 | Fig. 3, Suppl. Fig. 19 | |
| *Pemt – AcbSh* | F _(1, 16)_ = 0.15 | P=0.69 | F _(1, 16)_ = 0.31 | P=0.58 | F _(1, 16)_ = 0.43 | P=0.51 | Fig. 3, Suppl. Fig. 19 | |
| *Ptdss1 – AcbSh* | F _(1, 16)_ = 0.03 | P=0.85 | F _(1, 16)_ = 0.02 | P=0.87 | F _(1, 16)_ = 0.12 | P=0.73 | Fig. 3, Suppl. Fig. 19 | |
| *Ptdss2 – AcbSh* | F _(1, 16)_ = 0.09 | P=0.75 | F _(1, 16)_ = 0.18 | P=0.67 | F _(1, 16)_ = 0.13 | P=0.71 | Fig. 3, Suppl. Fig. 19 | |
| *Phospho1– AcbSh* | F _(1, 16)_ = 0.07 | P=0.78 | F _(1, 16)_ = 0.11 | P=0.74 | F _(1, 16)_ = 3.06 | P=0.09 | Fig. 3, Suppl. Fig. 19 | |
| *Pisd – AcbSh* | F _(1, 16)_ = 0.01 | P=0.94 | F _(1, 16)_ = 0.10 | P=0.75 | F _(1, 16)_ = 0.08 | P=0.77 | Fig. 3, Suppl. Fig. 19 | |
| *Lpcat4 – AcbSh* | F _(1, 16)_ = 2.58 | P=0.12 | F _(1, 16)_ = 0.03 | P=0.84 | F _(1, 16)_ = 2.18 | P=0.15 | Fig. 3, Suppl. Fig. 19 | |
| *Stard7 – AcbSh* | F _(1, 16)_ = 1.12 | P=0.30 | F _(1, 16)_ = 1.02 | P=0.32 | F _(1, 16)_ = 0.40 | P=0.53 | Fig. 3, Suppl. Fig. 19 | |
|  | **Treatment** | | **Genotype** | | **Treatment x Genotype** | |  | |
| ***Parameter*** | ***F (df)*** | ***P value*** | ***F (df)*** | ***P value*** | ***F (df)*** | ***P value*** | ***Figure*** | |
| *SAPT: time interacting* | F _(1, 28)_ = 9.46 | P<0.01 | F _(1, 28)_ = 31.1 | P<0.01 | F _(1, 28)_ = 2.35 | P=0.13 | Fig. 5 | |
| *SAPT: social preference index* | F _(1, 28)_ = 2.26 | P=0.14 | F _(1, 28)_ = 18.2 | P<0.01 | F _(1, 28)_ = 3.34 | P=0.07 | Fig. 5 | |
| *SPT: sucrose preference* | F _(1, 28)_ = 29.4 | P<0.01 | F _(1, 28)_ = 26.2 | P<0.01 | F _(1, 28)_ = 0.75 | P=0.39 | Fig. 5 | |
| *SPT: total intake* | F _(1, 28)_ = 0.89 | P=0.35 | F _(1, 28)_ = 0.02 | P=0.95 | F _(1, 28)_ = 0.81 | P=0.37 | Fig. 5 | |
| *TST: Immobility time* | F _(1, 28)_ = 0.94 | P=0.33 | F _(1, 28)_ = 14.1 | P<0.01 | F _(1, 28)_ = 0.09 | P=0.76 | Fig. 5 | |
| *OFT: Distance traveled* | F _(1, 28)_ = 0.02 | P=0.87 | F _(1, 28)_ = 0.26 | P=0.61 | F _(1, 28)_ = 0.21 | P=0.64 | Fig. 5 | |
| *OFT: Velocity* | F _(1, 28)_ = 0.53 | P=0.46 | F _(1, 28)_ = 0.07 | P=0.78 | F _(1, 28)_ = 0.53 | P=0.47 | Fig. 5 | |
| *OFT: Time in the center* | F _(1, 28)_ = 1.05 | P=0.31 | F _(1, 28)_ = 2.40 | P=0.13 | F _(1, 28)_ = 0.51 | P=0.47 | Suppl. Fig. 21 | |
| *PC(30.1)* | F _(1, 28)_ = 0.02 | P=0.95 | F _(1, 28)_ = 14.2 | P<0.01 | F _(1, 28)_ = 1.52 | P=0.22 | Fig. 5, Suppl. Fig. 24 | |
| *PC(32.0)* | F _(1, 28)_ = 0.84 | P=0.36 | F _(1, 28)_ = 1.38 | P=0.24 | F _(1, 28)_ = 5.46 | P<0.05 | Fig. 5, Suppl. Fig. 24 | |
| *PC(40.7)* | F _(1, 28)_ = 0.12 | P=0.72 | F _(1, 28)_ = 9.13 | P<0.01 | F _(1, 28)_ = 2.58 | P=0.11 | Fig. 5, Suppl. Fig. 24 | |
| *PC(38.1)* | F _(1, 28)_ = 4.14 | P=0.05 | F _(1, 28)_ = 13.2 | P<0.01 | F _(1, 28)_ = 1.46 | P=0.23 | Fig. 5, Suppl. Fig. 24 | |
| *PC(40.2)* | F _(1, 28)_ = 0.44 | P=0.13 | F _(1, 28)_ = 7.18 | P<0.01 | F _(1, 28)_ = 2.40 | P=0.13 | Fig. 5, Suppl. Fig. 24 | |
| *PC(42.7)* | F _(1, 28)_ = 0.53 | P=0.47 | F _(1, 28)_ = 9.59 | P<0.01 | F _(1, 28)_ = 3.32 | P=0.07 | Fig. 5, Suppl. Fig. 24 | |
| *PC(42.1)* | F _(1, 28)_ = 1.01 | P=0.32 | F _(1, 28)_ = 16.3 | P<0.01 | F _(1, 28)_ = 0.70 | P=0.40 | Fig. 5, Suppl. Fig. 24 | |
| *PC(44.12)* | F _(1, 28)_ = 6.98 | P<0.05 | F _(1, 28)_ = 1.65 | P=0.21 | F _(1, 28)_ = 0.10 | P=0.74 | Fig. 5, Suppl. Fig. 24 | |
| *PE-O(34.2)* | F _(1, 28)_ = 7.10 | P<0.05 | F _(1, 28)_ = 21.6 | P<0.01 | F _(1, 28)_ = 2.05 | P=0.16 | Fig. 5, Suppl. Fig. 26 | |
| *PE-O(36.3)* | F _(1, 28)_ = 10.5 | P<0.01 | F _(1, 28)_ = 1.81 | P=0.18 | F _(1, 28)_ = 1.98 | P=0.16 | Fig. 5, Suppl. Fig. 26 | |
| *PE-O(36.3)* | F _(1, 28)_ = 6.16 | P<0.05 | F _(1, 28)_ = 3.59 | P=0.06 | F _(1, 28)_ = 1.71 | P=0.20 | Fig. 5, Suppl. Fig. 26 | |
| *PE-O(38.3)* | F _(1, 28)_ = 2.59 | P=0.11 | F _(1, 28)_ = 3.26 | P=0.08 | F _(1, 28)_ = 5.41 | P<0.05 | Fig. 5, Suppl. Fig. 26 | |
| *PE-O(38.2)* | F _(1, 28)_ = 7.48 | P<0.05 | F _(1, 28)_ = 0.04 | P=0.82 | F _(1, 28)_ = 3.82 | P=0.06 | Fig. 5, Suppl. Fig. 26 | |
| *PE(38.6)* | F _(1, 28)_ = 1.94 | P=0.17 | F _(1, 28)_ = 7.27 | P<0.05 | F _(1, 28)_ = 4.73 | P<0.05 | Fig. 5, Suppl. Fig. 26 | |
| *PE(38.4)* | F _(1, 28)_ = 5.68 | P<0.05 | F _(1, 28)_ = 0.08 | P=0.77 | F _(1, 28)_ = 1.46 | P=0.23 | Fig. 5, Suppl. Fig. 26 | |
| *PE(40.6)* | F _(1, 28)_ = 18.9 | P<0.01 | F _(1, 28)_ = 2.91 | P=0.09 | F _(1, 28)_ = 3.28 | P=0.08 | Fig. 5, Suppl. Fig. 26 | |
| *PS(36.1)* | F _(1, 28)_ = 5.83 | P<0.05 | F _(1, 28)_ = 0.02 | P=0.88 | F _(1, 28)_ = 0.55 | P=0.46 | Fig. 5, Suppl. Fig. 28 | |
| *PS(38.2)* | F _(1, 28)_ = 2.30 | P=0.14 | F _(1, 28)_ = 8.57 | P<0.01 | F _(1, 28)_ = 5.42 | P<0.05 | Fig. 5, Suppl. Fig. 28 | |
| *PS(38.1)* | F _(1, 28)_ = 4.09 | P=0.05 | F _(1, 28)_ = 0.06 | P=0.79 | F _(1, 28)_ = 3.77 | P=0.06 | Fig. 5, Suppl. Fig. 28 | |
| *PS(40.2)* | F _(1, 28)_ = 1.18 | P=0.28 | F _(1, 28)_ = 23.1 | P<0.01 | F _(1, 28)_ = 3.04 | P=0.09 | Fig. 5, Suppl. Fig. 28 | |
| *PI(36.4)* | F _(1, 28)_ = 8.63 | P<0.05 | F _(1, 28)_ = 12.6 | P<0.01 | F _(1, 28)_ = 1.79 | P=0.19 | Fig. 5, Suppl. Fig. 28 | |
| *PI(36.1)* | F _(1, 28)_ = 0.69 | P=0.42 | F _(1, 28)_ = 1.73 | P=0.19 | F _(1, 28)_ = 6.67 | P<0.05 | Fig. 5, Suppl. Fig. 28 | |
| *PI(36.2)* | F _(1, 28)_ = 0.53 | P=0.46 | F _(1, 28)_ = 1.55 | P=0.22 | F _(1, 28)_ = 7.50 | P<0.05 | Fig. 5, Suppl. Fig. 28 | |
| *PI(38.4)* | F _(1, 28)_ = 4.06 | P=0.05 | F _(1, 28)_ = 0.05 | P=0.81 | F _(1, 28)_ = 2.63 | P=0.11 | Fig. 5, Suppl. Fig. 28 | |
| *PI(38.5)* | F _(1, 28)_ = 10.9 | P<0.01 | F _(1, 28)_ = 0.06 | P=0.93 | F _(1, 28)_ = 0.76 | P=0.38 | Fig. 5, Suppl. Fig. 28 | |
| *LPE(18.1)* | F _(1, 28)_ = 5.31 | P<0.05 | F _(1, 28)_ = 0.35 | P=0.55 | F _(1, 28)_ = 1.59 | P=0.21 | Fig. 5, Suppl. Fig. 30 | |
| *LPI(18.0)* | F _(1, 28)_ = 41.2 | P<0.01 | F _(1, 28)_ = 0.31 | P=0.58 | F _(1, 28)_ = 0.35 | P=0.55 | Fig. 5, Suppl. Fig. 30 | |
| *LPC(16.0)* | F _(1, 28)_ = 17.2 | P<0.01 | F _(1, 28)_ = 2.41 | P=0.13 | F _(1, 28)_ = 0.76 | P=0.38 | Fig. 5, Suppl. Fig. 30 | |
| *LPC(16.1)* | F _(1, 28)_ = 15.4 | P<0.01 | F _(1, 28)_ = 0.02 | P=0.86 | F _(1, 28)_ = 1.02 | P=0.32 | Fig. 5, Suppl. Fig. 30 | |
| *LPC(18.0)* | F _(1, 28)_ = 11.4 | P<0.01 | F _(1, 28)_ = 5.32 | P<0.05 | F _(1, 28)_ = 1.25 | P=0.27 | Fig. 5, Suppl. Fig. 30 | |
| *LPC(18.1)* | F _(1, 28)_ = 33.4 | P<0.01 | F _(1, 28)_ = 2.85 | P=0.10 | F _(1, 28)_ = 1.78 | P=0.19 | Fig. 5, Suppl. Fig. 30 | |
| *LPC(20.4)* | F _(1, 28)_ = 16.2 | P<0.01 | F _(1, 28)_ = 6.42 | P<0.05 | F _(1, 28)_ = 0.96 | P=0.33 | Fig. 5, Suppl. Fig. 30 | |
| *LPC(22.6)* | F _(1, 28)_ = 23.1 | P<0.01 | F _(1, 28)_ = 5.08 | P<0.05 | F _(1, 28)_ = 5.82 | P<0.05 | Fig. 5, Suppl. Fig. 30 | |

Two-way ANOVA.

**Supplementary Table 3.** Description of statistical data of the Kruskal–Wallis test

| ***Parameter*** | ***Statistic*** | ***P value*** | ***Figure*** |
| --- | --- | --- | --- |
| *Gnpat* | 139.9 | P<0.01 | Figure 4 |
| *Phospho1* | 66.92 | P<0.01 | Figure 4 |

Kruskal–Wallis test.
